# Identification of a diagnosis-selective neurobiological substrate for bipolar disorder, major depressive disorder, and schizophrenia: a meta-analysis of 57,717 subjects

**DOI:** 10.1017/S0033291726103511

**Published:** 2026-02-24

**Authors:** Donato Liloia, Paola Rocca, Claudio Brasso, Masaru Tanaka, Jordi Manuello, Annachiara Crocetta, Sergio Duca, Tommaso Costa, Franco Cauda

**Affiliations:** 1 Functional Neuroimaging and Complex Neural Systems (FOCUS) Laboratory, Department of Psychology, University of Turin, Turin, Italy; 2 Translational Neuroimaging & Brain Connectivity Group, GCS-fMRI Koelliker Hospital, Turin, Italy; 3 Department of Neuroscience “Rita Levi Montalcini”, University of Turin, Turin, Italy; 4 Neuroscience Institute of Turin (NIT), University of Turin, Turin, Italy; 5 HUN-REN-SZTE Neuroscience Research Group, Hungarian Research Network,University of Szeged, Danube Neuroscience Research Laboratory, Szeged, Hungary; 6 Neuroimaging & Data Science Group,GCS-fMRI Koelliker Hospital, Turin, Italy; 7 Department of Social and Human Science, University of Valle D’Aosta, Aosta, Italy; 8 Computational Neuroimaging & Complex Systems Group, GCS-fMRI Koelliker Hospital, Turin, Italy

**Keywords:** activation likelihood estimation, anterior cingulate, Bayes factor, psychiatric disorders, quantitative synthesis, reverse inference, structural MRI

## Abstract

**Background:**

Neuroimaging studies have consistently revealed neuroanatomical abnormalities in individuals with bipolar disorder (BD), major depressive disorder (MDD), and schizophrenia (SZ). However, it remains unknown whether and to what extent disorder-selective gray matter variations occur in these prominent psychiatric disorders. This study conducted a meta-analysis of 25 years of published voxel-based morphometry (VBM) research to assess the presence of selective and robust neuroanatomical substrates of gray matter variation in BD, MDD, and SZ.

**Methods:**

Peer-reviewed experiments encompassing subjects with target disorders were systematically searched in the MEDLINE database. Additionally, peer-reviewed data on 30 other psychiatric disorders and 65 neurological diseases were obtained from the BrainMap database. Experiments reporting whole-brain group comparisons between patients and healthy controls were included if they identified significant reductions in gray matter morphometry.

**Results:**

The data were analyzed using the Bayes fACtor mOdeliNg algorithm. A total of 1,021 VBM experiments were included, comprising 29,540 patients and 28,177 healthy controls. Primary analyses of psychiatric data revealed strong evidence of gray matter reduction in the right middle temporal gyrus for BD and the posterior dorsal anterior cingulate cortex for SZ (*P* ≥ 95% selectivity). The robustness of these findings was confirmed using the fail-safe method tailored to the neuroimaging meta-analytic environment. No selective findings were observed in additional analyses that included neurological diseases.

**Conclusions:**

Taken together, these findings offer a framework that underscores the significance of diagnosis-selective neural substrates in psychopathology, a new perspective that could inform distinct pathophysiological processes and assist in diagnosis and treatment.

## Introduction

Bipolar disorder (BD), major depressive disorder (MDD), and schizophrenia (SZ) collectively represent the second leading cause of disability worldwide and are associated with increased mortality from both natural causes and suicide (Global Burden of Disease, 2021). These complex and heterogeneous mental disorders are characterized by combinations of symptoms involving perceptions, emotions, thoughts, and behaviors. Current diagnostic criteria, which rely on partially overlapping symptom profiles, may result in diagnostic inaccuracies and suboptimal treatment outcomes (Richards et al., 2022). Over the past three decades, research in the field of brain mapping has sought to elucidate the pathophysiological mechanisms of these disorders to advance diagnostic, preventive, and therapeutic strategies. Evidence from structural magnetic resonance imaging (sMRI) technologies has highlighted consistent regional neuroanatomical variations across these clinical entities (Chen et al., 2022; Gray, Müller, Eickhoff, & Fox, 2020; Liloia et al., 2021). While the current corpus of sMRI findings has deepened our understanding of the neural phenotype of BD, MDD, and SZ, the anticipated clinical applicability of structural neuroimaging remains unrealized (Abi-Dargham et al., 2023; Stein et al., 2022).

One reason for this translational gap may lie in the tendency of mental illnesses to exhibit gray matter variations within a defined set of brain regions. Independent studies have identified abnormalities in hub nodes of the human connectome across distinct diagnostic entities (Cauda et al., 2019; Crossley et al., 2014; Hettwer et al., 2022; Kaczkurkin et al., 2019; Romer et al., 2021; Vanasse et al., 2021; Writing Committee for the Attention-Deficit/Hyperactivity Disorder et al., 2021), raising the question of whether gray matter variations represent a disorder-specific neurobiological phenotype or reflect a transdiagnostic marker of psychiatric disorders (Bourque et al., 2024; de Lange et al., 2019; Segal et al., 2024). For example, the meta-analysis by Goodkind et al. (2015), encompassing 15,892 participants across six psychiatric diagnoses (i.e. MDD, BD, SZ, addiction, obsessive-compulsive disorder, and anxiety), identified a common cluster of gray matter reductions in the dorsal anterior cingulate cortex (dACC), anterior insula (AI), ventromedial and dorsomedial prefrontal cortex, thalamus, amygdala, hippocampus, superior temporal gyrus, and parietal operculum. Wise et al. (2017) further confirmed a shared neuroanatomical reduction in the dACC, AI, and the dorsomedial prefrontal cortex in individuals with MDD and BD. Research conducted by Chang et al. (2018) also reported that BD, MDD, and SZ patients presented gray matter volume decreases in 87.9% of the total regional volume with significant variations in the dACC, subgenual ACC, posterior cingulate cortex (PCC), temporal pole, insula, parahippocampus, angular gyri, cuneus, orbital frontal, and dorsal lateral prefrontal cortices. In more recent years, a similar trend has emerged from multisite mega-analyses conducted by the Enhancing Neuro Imaging Genetics Through Meta Analysis (ENIGMA) consortium. Specifically, Okada et al. (2023) found a consistent reduction in hippocampal volume in individuals with BD and SZ, whereas Opel et al. (2020) demonstrated shared cortical abnormalities in BD, MDD, SZ, and obsessive-compulsive disorder, especially in the fusiform gyrus, hippocampus, and AI.

The potential existence and regional distribution of disorder-selective neuroanatomical variations that are associated with psychiatric conditions remain elusive. Tackling this deficiency in knowledge is important for multiple factors. First, uncovering disorder-selective neuroanatomical signatures could significantly support current endeavors in identifying robust neural biomarkers for differential diagnoses or measuring treatment outcomes in psychiatric disorders. Second, prioritizing research on targeted neural populations can facilitate the elucidation of the etiological mechanisms underlying clear-cut diagnostic categories, paving the way for targeted interventions, such as noninvasive brain stimulation. Third, addressing this translational challenge may refine current psychiatric nosology and ultimately inform future classifications and clinical assessments.

A secondary-level examination of the neuroanatomical variations tied to psychiatric disorders across the entire brain may be essential for answering this question. Specifically, 25 years of voxel-based morphometry (VBM) analysis (Ashburner & Friston, 2000) of sMRI data in clinical research, along with the establishment of accessible and largely automated neuroimaging data repositories such as BrainMap (Vanasse et al., 2018), provide an unparalleled resource for the systematic exploration of potential diagnosis-selective neural profiles for prominent psychiatric disorders. In the present study, we conducted a meta-analytic investigation of structural brain abnormalities across the whole brain, focusing on regional gray matter reductions derived from published VBM experiments using a traditional case–control design and analyzing 98 different diagnostic categories (33 psychiatric disorders and 65 neurological diseases). To achieve this goal, we introduce the Bayes fACtor mOdeliNg (BACON) approach (Costa et al., 2021), which integrates data from both mega- and meta-analyses to enable a quantitative, voxelwise characterization of diagnosis-selective neuroanatomical phenotypes in patients with BD, MDD, and SZ. Unlike conventional neuroimaging approaches using disorder-to-alteration mapping (Liloia, Costa, Cauda, & Manuello, 2024), BACON provides an alteration-to-disorder framework, quantifying the posterior probability that an observed structural variation is selectively associated with a target diagnosis relative to variations reported across other clinically defined categories.

## Methods and materials

### Data search

Adhering to PRISMA (Page et al., 2021) and neuroimaging meta-analysis (Müller et al., 2018) guidelines, we identified independent VBM experiments contrasting gray matter in healthy controls with patients diagnosed with BD, MDD, and SZ. Additionally, VBM experiments involving comparisons of healthy controls with other psychiatric disorders or neurological diseases stored in the BrainMap database (Vanasse et al., 2018) were included. Only coordinates reporting gray matter volume/concentration decreases (i.e. healthy controls > disorders of interest) in a standardized stereotactic space were selected. To increase spatial accuracy, analyses were conducted in Talairach space with Montreal Neurological Institute (MNI) coordinates converted to Talairach space using the icbm2tal algorithm (Laird et al., 2010). A detailed description of eligibility criteria and search methodology is provided in the eMethods section in the Supplement. Since the study utilized published peer-reviewed data, institutional review board approval and patient consent were not required.

### Database construction

For each experiment, coordinates of gray matter variations derived exclusively from whole-brain analyses were extracted. Coordinates based on region-of-interest or small-volume correction VBM analyses were excluded to avoid artificial biases favoring specific brain regions (Müller et al., 2018). Using the CBMAT toolbox (Manuello et al., 2023), gray matter coordinates located in the cerebellum or outside the gray matter mask were excluded, as the cerebellum is frequently omitted in MRI scans, potentially introducing unreliability (Van Overwalle et al., 2024). This exclusion also mitigates spurious clusters of selective variation in white matter structures.

**Figure 1.**
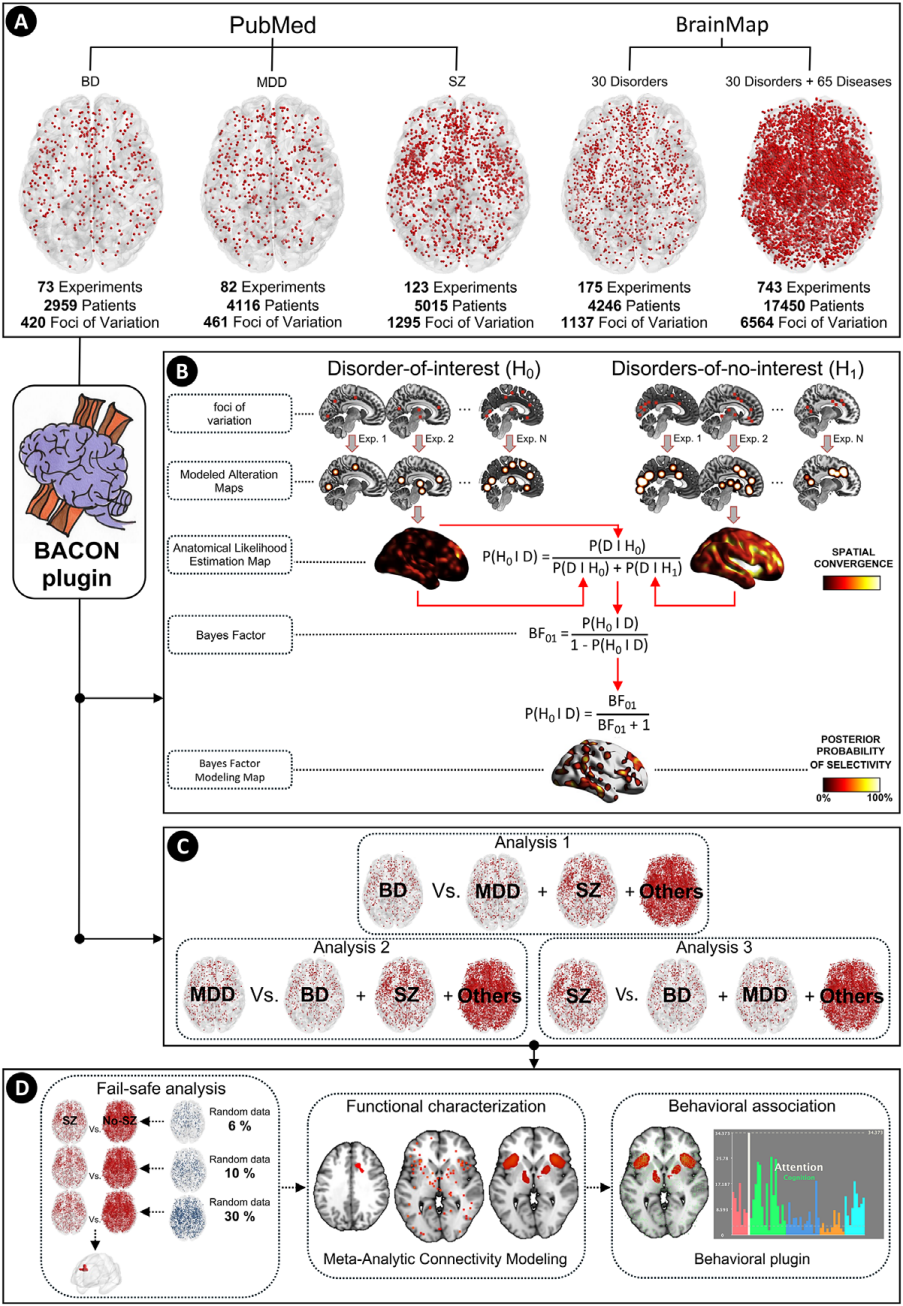
Overview of the analytical procedures. (A) A total of 8,740 coordinates of significant gray matter variation from 29,540 patients were extracted from 1,021 published voxel-based morphometry experiments. (B) Graphical representation of the data analytic pipeline, from the variation coordinates to the selectivity whole-brain map. The final statistical parametric map, which represents the values of the selective probability of the disorder of interest, is obtained with the Bayes factor computation implemented in the Bayes fACtor mOdeliNg plugin. (C) Data groupings for estimating the selectivity of the gray matter landscape in BD (analysis 1), MDD (analysis 2), and SZ (analysis 3) patients. (D) Robustness and supplementary functional and behavioral analyses of the Bayes fACtor mOdeliNg results. *Note:* BACON = Bayes fACtor mOdeliNg; BD = bipolar disorder; BF = Bayes factor; EXP. = experiment; MDD = major depressive disorder; SZ = schizophrenia.

These steps allowed us to define five data groupings essential for subsequent analyses: (1) the BD dataset, composed of coordinates describing gray matter variations in patients with BD (versus healthy control subjects); (2) the MDD dataset, with coordinates of gray matter variations in patients with MDD (versus healthy control); (3) the SZ dataset, with coordinates of gray matter variations in patients with SZ (versus healthy control); (4) the BrainMap psychiatric dataset, composed of coordinates describing gray matter variations in patients with other psychiatric disorders (versus healthy control subjects), sourced from the BrainMap database; and (5) the BrainMap psychiatric and neurological dataset, composed of coordinates describing gray matter variations in patients with other psychiatric disorders and neurological diseases (versus healthy control subjects) sourced from the BrainMap database. Further details of the database construction are provided in Figure 1A.

### Bayes fACtor mOdeliNg

We applied the BACON algorithm (Costa et al., 2021) to estimate posterior probabilities for gray matter variations selectively associated with BD, MDD, and SZ. This Bayesian-based approach evaluates the likelihood that observed neuroanatomical variations correspond to a disorder of interest.

We first conducted two meta-analyses via the anatomical likelihood estimation (ALE) method (Eickhoff et al., 2012) for each disorder of interest: the first based on the coordinates of the disorder under investigation (e.g. the SZ dataset), and the second based on the coordinates of everything but the disorder under investigation coordinates (e.g. the merging of the BD, MDD, and BrainMap psychiatric datasets) (Figure 1B). The ALE algorithm, as implemented in the GingerALE software (v.3.0.2), was employed. The ALE method models the coordinates from each experiment as a series of three-dimensional Gaussian distributions of probabilities centered on the peaks of variations reported by the included experiments (Eickhoff et al., 2016). This permits the generation of a modeled alteration map for each experiment included in the meta-analysis. The size of the Gaussian kernel varies between the modeled alteration maps, taking into account the sample size originally used in each experiment. The union of all modeled alteration maps produces whole-brain voxelwise ALE scores, which quantify the degree of overlap among reported results at specific brain locations (Eickhoff et al., 2012) (Figure 1B). A comprehensive statistical explanation of the method can be found in the eMethods section of the Supplement.

Second, we utilized the BACON algorithm as implemented in the MANGO software (v.4.1). BACON combines Bayes factor (Kass & Raftery, 1995) analysis with ALE-derived maps to determine the likelihood that observed neuroanatomical variations in a particular voxel are selectively associated with the disorder of interest rather than with other conditions (e.g. SZ versus no-SZ). This approach allowed us to evaluate two competing hypotheses in a whole-brain voxelwise manner: one positing that the variation is most likely related to the disorder under investigation and another suggesting that it is also associated with other conditions. In the absence of knowledge about the prior probabilities of these hypotheses, they are treated as equally likely, a choice supported by previous validation studies (Cauda et al., 2020; Costa et al., 2021; Liloia et al., 2023). This assumption ensures that the computed Bayes factor directly reflects the relative strength of evidence for the disorder of interest compared with alternative clinical conditions. Thus, BACON computes posterior probabilities to quantify the likelihood that the gray matter variation observed in a voxel is selectively associated with the disorder under investigation, representing the probability *P (disorder of interest | variation).* A detailed statistical explanation is provided in the eMethods section of the Supplement, along with a visual illustration in Figure 1B.

### Main analyses (psychiatric disorders)

We conducted three primary analyses (Figure 1C) to identify clusters showing diagnosis-selective structural variation for BD, MDD, and SZ relative to other psychiatric disorders in the BrainMap database. Results were thresholded at *P (disorder of interest | variation*) 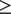 0.95 (i.e. a posterior probability of diagnosis-selectivity ≥ 0.95 for the target disorder) with a minimum cluster size of 500 mm^3^. This posterior-probability threshold is interpreted on the Bayes factor scale as ‘strong evidence’, following the classification of evidence strength by Kass and Raftery (1995).

### Additional analyses (psychiatric disorders and neurological diseases)

Three secondary analyses were also conducted for BD, MDD, and SZ patients, specifically combining psychiatric disorders and neurological diseases within the non-interest dataset. Consistent with the primary analyses, results were thresholded at *P (disorder of interest | variation)* 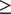 0.95.

### Robustness analyses

Meta-analytic findings may be influenced by the file-drawer problem, a form of publication bias where experiments with null or contradictory results remain unpublished. To address this issue, we assessed the robustness of our findings via the fail-safe technique adapted for the neuroimaging meta-analytic environment (Acar, Seurinck, Eickhoff, & Moerkerke, 2018). Based on a recent simulation study (Samartsidis et al., 2020), estimating a 6% rate of missing experiments in the BrainMap database, we retested our analyses by introducing an equivalent percentage of noise (i.e. random simulated coordinates of variation) to our datasets of no interest (Figure 1D). Robustness was further evaluated by increasing noise levels up to 30%, in line with recent recommendations (Gray et al., 2020). Details are provided in the eMethods in the Supplement.

### Functional and behavioral analyses

Supplementary post hoc analyses were conducted to further characterize and interpret the robust meta-analytic cluster identified via BACON. These analyses encompassed task-based coactivation characterization via meta-analytic connectivity modeling (MACM) (Laird et al., 2013) and observer-independent brain-to-behavior association via the Behavioral plugin (Lancaster et al., 2012) (Figure 1D). The analyses were performed using the BrainMap functional database (Fox & Lancaster, 2002; Laird et al., 2009), which focuses on functional MRI experiments of healthy participants involved in normal mapping task-based experiments (8,377 eligible experiments). MACM was determined via ALE-based methods with a cluster-level familywise error correction threshold of *P* < 0.05 and a cluster-forming threshold of *P* < 0.001 (Eickhoff et al., 2016). A Bonferroni-corrected threshold at *P* < 0.05 was adopted to designate statistically significant behavioral associations (Lancaster et al., 2012). A detailed description of these analyses can be found in the eMethods section in the Supplement.

## Results

**Figure 2.**
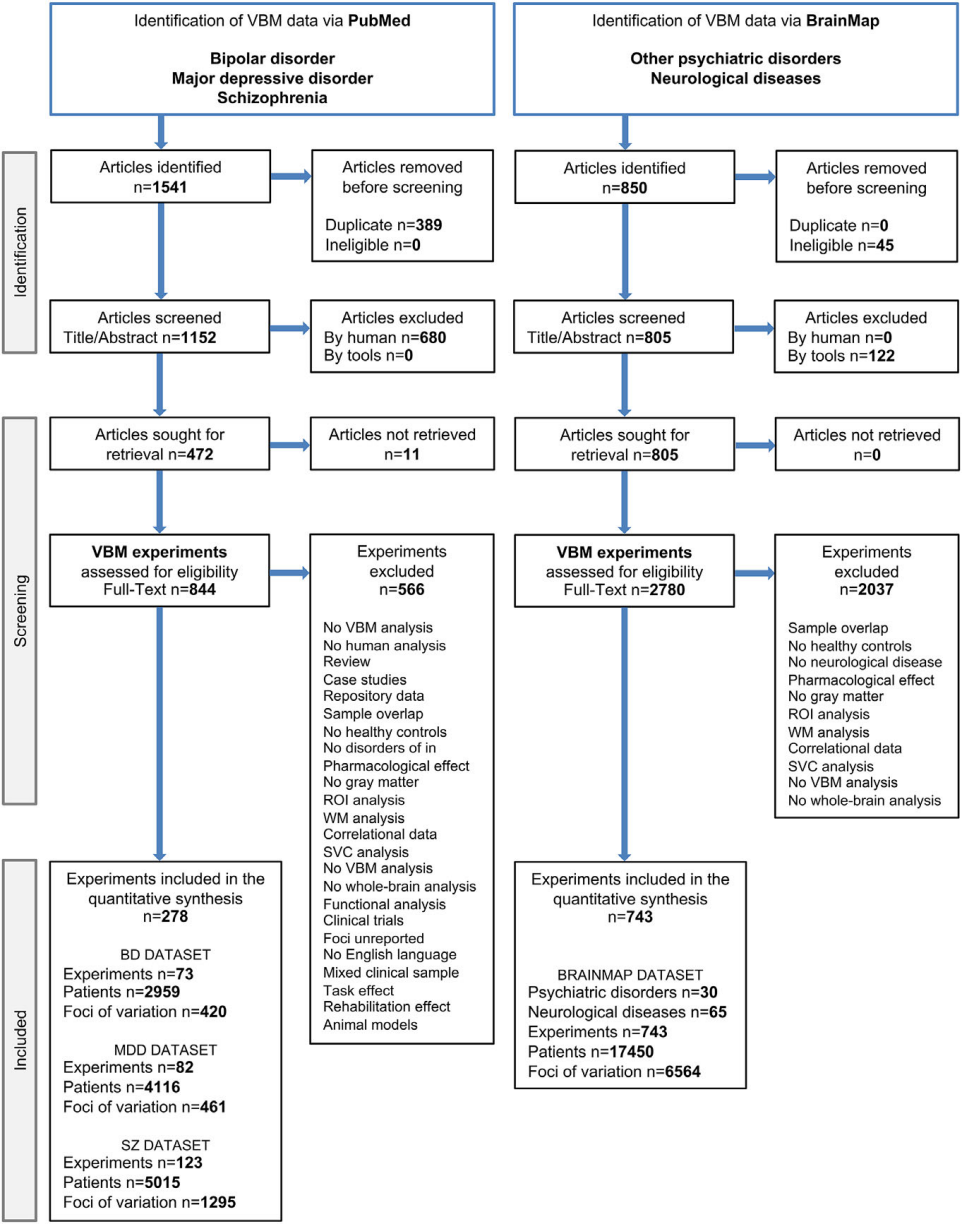
Overview of literature selection and coding (PRISMA flowchart). *Note:* BD = bipolar disorder; MDD = major depressive disorder; N = number of; ROI = region-of-interest; SZ = schizophrenia disorder; SVC = small volume correction; WM = white matter; VBM = voxel-based morphometry.

A total of 1,021 individual neuroimaging experiments, comprising 29,540 patients and 28,177 healthy controls, were included in the study (Figure 2). The included diagnostic groups were BD (73 VBM experiments; Supplementary Table S1), MDD (82 VBM experiments; Supplementary Table S2), SZ (123 VBM experiments; Supplementary Table S3), other psychiatric disorders from the BrainMap database (30 different disorders and 175 VBM experiments; Supplementary Table S4), and other psychiatric disorders combined with neurological diseases from the BrainMap database (95 diagnostic categories and 743 VBM experiments; Supplementary Table S5). Further details of these groups are provided in Figure 1A, Supplementary Tables S6 and S7.

### Selective variation profile of bipolar disorder

**Figure 3.**
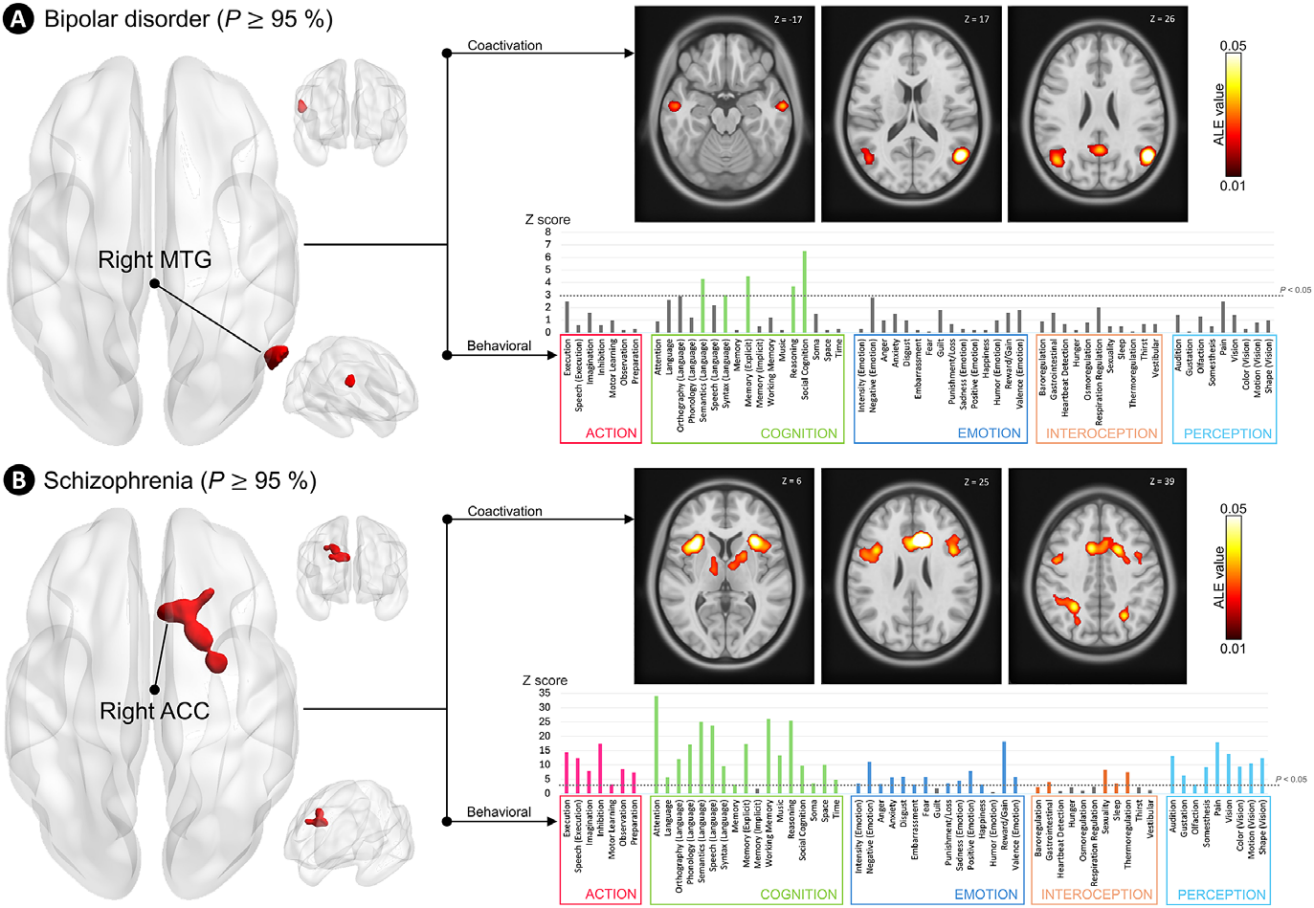
Robust clusters of selective gray matter variation in bipolar disorder and schizophrenia and their network-level functional and behavioral characterizations. (A) Brain cluster of selective gray matter variation in bipolar disorder derived from Bayes fACtor mOdeliNg analysis of psychiatric disorders data thresholded at *P* (*bipolar disorder | variation*) 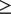 0.95 (95%) and remaining stable over 30% of injected random experiments via fail-safe analysis. On the right side, the meta-analytic coactivation map (MACM) shows areas coactivated with the cluster of variation in healthy participant task-based activation experiments in the BrainMap database, as well as its statistically linked physiological mental processes derived from the behavioral analysis of the BrainMap database. (B) Brain cluster of selective gray matter variation in schizophrenia derived from Bayes fACtor mOdeliNg analysis of psychiatric disorders data thresholded at *P* (*schizophrenia | variation*) 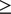 0.95 (95%) and remaining stable over 30% of injected random experiments via fail-safe analysis. On the right side, the MACM shows areas coactivated with the cluster of variation in healthy participant task-based activation experiments in the BrainMap database, as well as its statistically linked physiological mental processes derived from the behavioral analysis of the BrainMap database. The Bayes fACtor mOdeliNg results are visualized as hemispheric surfaces (three-dimensional view). MACM results are visualized as axial slices (two-dimensional cortical and subcortical views). Templates are in neurological convention. *Note:* ACC = posterior dorsal anterior cingulate cortex; ALE = activation likelihood estimation; MTG = middle temporal gyrus.

The primary BACON analysis using psychiatric data (Supplementary Tables S4 and S6) revealed three clusters of variation at *P* 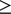 0.95. Specifically, we observed gray matter selectivity for BD patients in the right middle frontal gyrus, right middle temporal gyrus (MTG), and left inferior parietal lobule (Table 1A). However, fail-safe analysis revealed that only the cluster in the right MTG was robust (Figure 3A), withstanding simulated data injection by 30% of experiments, whereas the others were negated with just 6% of injected simulated coordinates, which is consistent with the estimated proportion of missing experiments in the BrainMap database (Samartsidis et al., 2020).

**Table 1.** Clusters of selective gray matter variation in bipolar disorder (A), major depressive disorder (B), and schizophrenia (C) derived from Bayes fACtor mOdeliNg analysis of psychiatric-only data and thresholded at *P* (*disorder-of-interest | variation*) 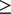 0.95 (95%)

Cluster ID	Brain region (Local maxima)	Talairach x y z	Cluster size mm^3^	BACON value		Robustness (Fail-safe %)
				Maximum	Minimum	
BD						
1	Right middle frontal gyrus (BA 6)	22 12 62	816	0.98896	0.95007	< 6%
**2**	**Right middle temporal gyrus (BA 39)** ^a^	**56 –64 24**	**648**	**0.99779**	**0.95004**	**> 30%**
3	Left inferior parietal lobule (BA 40)	−52 –34 44	604	0.99132	0.95013	< 6%
MDD						
	No clusters found					
SZ						
**1**	**Right anterior cingulate cortex (BA 32)** ^a^	**20 10 36**	**1812**	**0.99487**	**0.95003**	**> 30%**
2	Right postcentral gyrus (BA 2)	61 –22 37	915	0.97531	0.95002	< 6%

Abbreviations: BA = Brodmann area; BACON = Bayes fACtor mOdeliNg.

a Robust clusters of variation selectivity, remaining stable over 30% of injected simulated experiments.

To determine whether this selective cluster is normally part of a coherent functional circuit, we examined its pattern of task-dependent coactivation. MACM analysis revealed significant coactivation with the left middle and superior temporal gyri (Brodmann areas, BAs 39), as well as the bilateral PCC (BAs 31), and the inferior temporal gyri (BAs 21) (Figure 3A and Supplementary Table S8). Thus, we used the Behavioral plugin to evaluate the corresponding physiological mental processes statistically associated with the network of coactivation. This resulted in the identification of six subdomains, which reflected the cognitive functioning domain (i.e. social cognition, explicit memory, language semantics, reasoning, and language syntax). Further details are provided in Figure 3A and Supplementary Table S9.

In contrast, the additional BACON analysis of psychiatric and neurological conditions did not reveal any selective gray matter clusters for BD at *P* 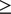 0.95.

### Selective variation profile of major depressive disorder

The primary BACON analysis and additional analysis revealed no selective gray matter clusters for MDD at thresholds of *P* 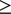 0.95.

### Selective variation profile of schizophrenia

The primary BACON analysis revealed two clusters of variation at *P* 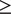 0.95. Specifically, we observed gray matter selectivity for SZ patients in the right posterior dACC (also known as caudal ACC) (Table 1C). Fail-safe analysis confirmed the robustness of the right posterior dACC cluster, which remained stable across 30% of simulated coordinates. However, the other cluster was negated with 6% of simulated data injections (Figure 3B).

MACM demonstrated significant coactivation of the right posterior dACC with several regions, including the left ACC, bilateral middle frontal gyri (BAs 6), AI (BA 13), inferior frontal gyri (BAs 9 and 44), precuneus (BA 7), fusiform gyri (BAs 19 and 37), putamen, globus pallidum, and thalamus (Figure 3B and Supplementary Table S10). The corresponding physiological mental processes statistically associated with the right posterior dACC network of coactivation were 49, which reflected all functioning domains of the BrainMap database (i.e. action, cognition, emotion, interoception, and perception). Details can be found in Figure 3B and Supplementary Table S11.

The additional BACON analysis of psychiatric and neurological conditions did not identify any selective gray matter clusters for SZ at *P* 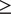 0.95.

## Discussion

Leveraging 25 years of peer-reviewed VBM clinical research, this study yielded several important findings. We provide strong evidence of robust selectivity in the neuroanatomical phenotypes associated with BD and SZ, compared to other psychiatric conditions. Furthermore, we reveal that selective variation appears to accumulate in well-delineated high-order areas, which display extensive coactivation across large-scale networks and are consistently engaged in cognitive domains impaired in these disorders.

Building on the foundational work of Goodkind et al. (2015), extensive brain mapping research has identified a shared neuroanatomical substrate across mental illnesses (Hettwer et al., 2022; Kaczkurkin et al., 2019; Okada et al., 2023; Opel et al., 2020; Romer et al., 2021; Wise et al., 2017). In this study, we advanced this line of inquiry by adopting a data-driven, hypothesis-free approach to examine whether BD, MDD, and SZ are distinguished by selective structural brain phenotypes. Our primary analyses identified localized multimodal regions in the right hemisphere for both BD and SZ. However, other regions considered neuroanatomical markers of these disorders (i.e. amygdala, basal ganglia, dorsal and subgenual ACC, fusiform gyrus, hippocampus, inferior frontal gyrus, insula, prefrontal cortex, temporal pole, and thalamus) (Chen et al., 2022; Liloia et al., 2021) exhibited no significant selectivity, indicating their shared involvement across multiple psychiatric categories. These findings expand prior transdiagnostic research by integrating whole-brain cross-disorder similarity in morphometry-derived phenotypes with disorder-selective loci previously uncharacterized.

We found robust clusters of selective variation in the right MTG and posterior dACC for BD and SZ, respectively. These findings are particularly significant given prior VBM meta-analyses (Chen et al., 2022; Liloia et al., 2021; Wise et al., 2017; Zhu et al., 2022) that have consistently reported morphometric aberrations in these regions across primary investigations. This reinforces their potential role in current neurobiological models of BD and SZ. The MTG has been repeatedly mentioned in neuroimaging research of BD, showing cortical thickness and functional reductions (Hibar et al., 2018; Kuang et al., 2022; Long et al., 2022), along with disruptions in both long- and short-range connectivity (Liu et al., 2022; Syan et al., 2017; Wang et al., 2016). In line with our observer-independent behavioral analyses, this area has been shown to support key functions aberrant in the disorder, such as social cognition, reasoning, semantic processing, and memory (Keramatian, Torres, & Yatham, 2021). Therefore, these findings offer a new perspective on the importance of this region, which may provide critical insights into core cognitive impairments in BD. Future multidisciplinary diagnostic research integrating the right MTG is warranted.

Similarly, the selective involvement of the right posterior dACC represents a notable finding for translational SZ research. The ACC has been widely recognized as a key morpho-functional hub in SZ (Baiano et al., 2007; Vitolo et al., 2017; Wang et al., 2015) and identified as a putative epicenter of neural tissue loss (Shafiei et al., 2020). However, its extensive involvement across various mental illnesses (Cauda et al., 2019; Chang et al., 2018; Goodkind et al., 2015; Sha, Wager, Mechelli, & He, 2019; Wise et al., 2017) renders our findings unique. Importantly, the dACC cluster identified in our study does not overlap with the previously reported gray matter loss in the ACC observed across psychiatric categories in transdiagnostic studies such as Goodkind et al. (2015). Instead, our findings suggest that the more caudal portions of the dACC may have a central role in SZ, in contrast to the dorsal anterior and subgenual regions commonly implicated across psychiatric disorders (Chang et al., 2018; Goodkind et al., 2015; Wise et al., 2017). Our results are consistent with the ‘schizophrenia neural common network’ described via coordinate network mapping (CNM) (Makhlouf et al., 2025), a multimodal approach incorporating a network-level design to address inter-subject heterogeneity in VBM studies. Similarly, they align with a recent mega-analysis from the ENIGMA consortium, which identified low structural heterogeneity in cortical folding of the right caudal ACC across a multi-site cohort of 5,626 subjects with SZ (Omlor et al., 2025). Although our BACON-derived cluster was more sensitive than the CNM-defined and ROI-based cortical findings, this concordance emphasizes the value of integrating diverse neuroimaging methodologies to enhance our understanding of the neurobiological foundations of SZ, facilitating the development of targeted interventions.

No clusters of variation were found in MDD. This result is consistent with the lack of spatial consistency in gray matter reduction identified by recent VBM meta-analyses (Gray et al., 2020; Müller et al., 2017), which cannot be attributed to insufficient statistical power. Importantly, multisite mega-analysis studies from the ENIGMA consortium (Cheon et al., 2022; Hibar et al., 2018, 2016; Schmaal et al., 2017, 2020; van Erp et al., 2016, 2018) further suggest an ‘affective-psychotic severity brain continuum’, with widespread cortical and subcortical abnormalities in SZ, intermediate involvement in BD, and more localized, less pronounced variations in MDD. In parallel, differences in average clinical burden across these disorders, including greater functional impairment and a higher burden of severe psychotic symptoms in SZ, intermediate levels in BD, and psychotic features in MDD largely confined to specific subtypes (Aminoff et al., 2022; Bowie et al., 2010; Jääskeläinen et al., 2018; Ohayon & Schatzberg, 2002), may plausibly be associated with more spatially consistent neuroanatomical alterations in SZ and, to a lesser extent, BD relative to MDD. Furthering this narrative, MDD represents a heterogeneous disorder that, according to the DSM-5 or ICD-11 criteria, may be diagnosed with more than 200 different combinations of signs and symptoms pooling together, within the same nosographic category. These patients can exhibit completely different sets of psychopathological manifestations (Zimmerman et al., 2015). This phenotypic heterogeneity corresponds to a marked diversity in genetic and environmental risk factors, which, interacting with each other in a multitude of different ways, could underlie the observed variability at the neural level (Müller et al., 2017; Nguyen et al., 2022; Zhang et al., 2023).

The additional analysis of BD, MDD, and SZ data against 65 neurological diseases revealed that regional reductions identified via the VBM technique exhibit a distributed, nonselective pattern crossing psychiatric and neurological boundaries. This finding corroborates previous studies (Cauda et al., 2019, 2018; Crossley et al., 2014; de Lange et al., 2019; Vanasse et al., 2021) demonstrating a shared neural architecture across psychiatric and neurological conditions. Moreover, pathophysiological commonalities have been consistently observed at multiple biological levels, including genetic and molecular evidence (Bryois et al., 2022; Cauda et al., 2018; Peall, Owen, & Hall, 2024; Smeland et al., 2025). Our findings are also aligned with the cross-disorder dysconnectivity hypothesis of the human connectome (van den Heuvel & Sporns, 2019). This is particularly evident in neurodegenerative diseases, where processes such as the transneuronal transportation of toxic misfolded proteins, trophic failure, and nodal stress degeneration drive neural death and atrophy (Raj & Powell, 2018). These processes result in network-like degeneration patterns that closely mirror the structural, functional, and genetic architecture of brain connectivity (Cauda et al., 2018; Fornito, Zalesky, & Breakspear, 2015; Raj & Powell, 2018). Importantly, over the disease course, these degeneration patterns gradually spread throughout the brain (Wilson et al., 2023), suggesting the possibility that widespread neural disruption encompasses regions implicated in psychiatric disorders. This raises the intriguing hypothesis that the observed degeneration patterns might reflect a unifying neurobiological substrate, potentially linking psychiatric and neurological conditions within a common pathophysiological framework (Cauda et al., 2019; Crossley et al., 2014; de Lange et al., 2019).

From a neurobiological perspective, do the present results support a categorical or a neural general dimension of psychopathology? We have demonstrated that localized, diagnosis-selective variations can be associated with BD and SZ. Nevertheless, the low probability of selectivity we identified across the rest of the brain is also reminiscent of recent dimensional models, such as the general psychopathology factor (P-factor) (Caspi et al., 2014). This model posits that the meta-structure of mental illnesses and their neural equivalent can be understood hierarchically, comprising both a single general dimension and several specific dimensions at the lower levels of the hierarchy (Caspi & Moffitt, 2018; Sprooten, Franke, & Greven, 2022). While this study provides potential neural insights that may contribute to the interpretation of a ‘neural P-factor’, the validity and substantive meaning of this dimension remain subjects of ongoing debate (DeYoung et al., 2022; Haeffel et al., 2022; Sprooten et al., 2022). Finally, it is necessary to note that the underlying cellular mechanisms responsible for changes in VBM signals in mental illnesses remain elusive (Mancuso et al., 2020). Understanding the pathophysiological principles driving neuroanatomical variations is a crucial next step for unraveling the biological basis of disorder variability and selectivity. We aspire that the presented meta-analytic features shall facilitate and provide a novel coordinate frame for such translational insights.

## Methodological considerations

The current meta-analytic study proposes a novel and statistically rigorous perspective on the complex neuroanatomical architecture underlying three prominent psychiatric disorders. In comparison to prior qualitative reviews and meta-analyses, our methodological design advances the field on several levels. We assembled the largest VBM meta-analytic dataset to date, examining data from 98 distinct diagnostic categories. This approach allowed us to optimize the balance between sensitivity and susceptibility to false-positive effects, thereby enhancing the statistical power of our quantitative synthesis (Manuello, Costa, Cauda, & Liloia, 2022; Müller et al., 2018). Also, the innovative transdiagnostic approach allowed us to extend the clinical information given by canonical neuroimaging meta-analyses focusing on a single disorder of interest, providing valuable insights into the selective role of each aberrant neuroanatomical component of clinical conditions under investigation (Cauda et al., 2020; Liloia et al., 2024). Not less important, by employing a Bayesian-based framework, we moved beyond binary frequentist concepts of statistical significance (i.e. dichotomous reject/do-not-reject framework), providing a direct probabilistic hypothesis assessment (Costa et al., 2021; Friston et al., 2002). Finally, although this study proposed a new outlook on the selective brain architectures of BD, MDD, and SZ, it is important to note that the BACON methodology can potentially be applied to any other clinical condition reporting neuroanatomical variations, opening attractive translational prospects for an in-depth comprehension of the clinical brain.

Despite these strengths, several limitations warrant consideration. By definition, neuroimaging meta-analyses are associated with lower spatial resolution than native statistical parametric maps, as they rely on reported stereotactic coordinates rather than full voxelwise data (Müller et al., 2018). Although our experimental design employed the anatomical ALE, the most widely used meta-analytic algorithm worldwide with demonstrated unbiased spatial reconstruction (Eickhoff et al., 2016; Manuello et al., 2022; Müller et al., 2018; Radua & Mataix-Cols, 2012; Tahmasian et al., 2019), this constraint remains inherent. At the same time, it is important to note that in the absence of a publicly available repository of peer-reviewed voxelwise whole-brain MRI data, using coordinate-based databases such as BrainMap remains the unique approach to exploring disorder-selective brain variations. Nevertheless, coordinate-based inference is also contingent on the reporting practices of published VBM studies (Manuello et al., 2022). In particular, the historically incomplete reporting of GM increases in the psychiatric primary literature can affect meta-analytic synthesis of ‘healthy controls < disorders of interest’ contrasts (Mancuso et al., 2020), potentially introducing reporting-related bias and undermining interpretability when increases are the target of inference. For this reason, we did not include a dedicated case–control analysis focused on GM increases in the present work. At the same time, systematically characterizing increase-selective GM effects remains an important target for future research. Still, the cross-sectional secondary-level design precludes analysis of the potential impact on findings of key sociodemographic and clinical variables (e.g. age, sex, education, illness duration, presence of psychotic features during mood episodes, and medication status). While we do not consider this a limitation *per se*, as the primary aim of a neuroimaging meta-analysis is to overcome sample heterogeneity and identify invariant findings across groups of interest (Eickhoff et al., 2012; Fox, Lancaster, Laird, & Eickhoff, 2014; Manuello et al., 2022; Tahmasian et al., 2019), disregarding the unique characteristics of individual subjects or diagnostic subgroups may be overly simplistic and risk overlooking critical features. We propose that future research could develop subject-level implementations of the BACON methodology to assess the neuroanatomical selectivity of variations and explore whether this approach can discriminate between different categories or dimensions within the disorder of interest. Currently, the principal challenge in extending this methodology to subject-level data lies in identifying focal neuroanatomical variations in the absence of normative intensity values, which are necessary to reliably distinguish T1 images of healthy versus clinical subjects (Arbabshirani, Plis, Sui, & Calhoun, 2017; Bzdok & Karrer, 2021; Liloia et al., 2021; Scarpazza & Simone, 2016).

## Conclusions

Our secondary level findings indicate that diagnosis-selective and robust neuroanatomical variations are identifiable in BD and SZ, but not in MDD. These results refine our understanding of the neuroanatomy of these complex disorders, opening attractive prospects for future neuroimaging-based translational research.

## Supporting information

10.1017/S0033291726103511.sm001 Liloia et al. supplementary material Liloia et al. supplementary material

## Data Availability

The datasets generated during and/or analyzed during the current study are available from the corresponding author on reasonable request.

## Supplementary material

The supplementary material for this article can be found at http://doi.org/10.1017/S0033291726103511.
