# Supplementary material for: Identification of a diagnosis-selective neurobiological substrate for bipolar disorder, major depressive disorder, and schizophrenia: a meta-analysis of 57,717 subjects
Source: Psychol Med. 2026 Feb 24;56:e52. doi: 10.1017/S0033291726103511 (PMC12969203; doi:10.1017/S0033291726103511)
Supplement: Liloia et al. supplementary material [file S0033291726103511sup001.docx]

**Supplementary material**

**Identification of a Diagnosis-Selective Neurobiological Substrate for Bipolar Disorder, Major Depressive Disorder, and Schizophrenia: A Meta-Analysis of 57,717 Subjects**

**eMethods 1.** Data search

**eMethods 2.** Data selection

**eMethods 3.** Anatomical likelihood estimation analysis

**eMethods 4.** Bayes fACtor mOdeliNg analysis

**eMethods 5.** Fail-safe analysis

**eMethods 6.** Functional and behavioral analyses

**eTable 1.** Experiments included in Bayes fACtor mOdeliNg analysis: BD dataset

**eTable 2.** Experiments included in Bayes fACtor mOdeliNg analysis: MDD dataset

**eTable 3.** Experiments included in Bayes fACtor mOdeliNg analysis: SZ dataset

**eTable 4.** Experiments included in Bayes fACtor mOdeliNg analysis: BrainMap psychiatric disorders dataset

**eTable 5.** Experiments included in Bayes fACtor mOdeliNg analysis: BrainMap psychiatric disorders and neurological diseases dataset

**eTable 6.** Distribution of the VBM data included (main analysis)

**eTable 7.** Distribution of the VBM data included (additional analysis)

**eTable 8.** Meta-analytic connectivity modeling of the right middle temporal gyrus

**eTable 9.** Behavioral results of the right middle temporal gyrus MACM

**eTable 10.** Meta-analytic connectivity modeling of the right anterior cingulate cortex

**eTable 11.** Behavioral results of the right anterior cingulate cortex MACM

**eReferences.**

This supplementary material has been provided by the authors to give readers additional information about their work.

**eMethods 1.** Data search

A systematic literature search was performed covering all peer-reviewed articles from inception to December 1, 2024, without restrictions on the publication year. The MEDLINE database (<https://pubmed.ncbi.nlm.nih.gov/>) was accessed via the PubMed search engine to identify voxel-based morphometry (VBM) experiments reporting data related to subjects with bipolar disorder (BD), major depressive disorder (MDD), and schizophrenia (SZ). Separate queries were constructed for each disorder as follows:

• “BD” query in MEDLINE (using PubMed Advanced Search Builder):

(“Bipolar disorder” [Title/Abstract] OR “BD” [Title/Abstract] OR “Bipolar” [Title/Abstract]) AND (“voxel-based morphometry” [Title/Abstract] OR “VBM” [Title/Abstract]);

• “MDD” query in MEDLINE (using PubMed Advanced Search Builder):

(“Major depressive disorder” [Title/Abstract] OR “MDD” [Title/Abstract] OR “Depression” [Title/Abstract]) AND (“voxel-based morphometry” [Title/Abstract] OR “VBM” [Title/Abstract]);

• “SZ” query in MEDLINE (using PubMed Advanced Search Builder):

(“Schizophrenia” [Title/Abstract] OR “SZ” [Title/Abstract]) AND (“voxel-based morphometry” [Title/Abstract] OR “VBM” [Title/Abstract]).

In addition, another standardized search was conducted using the BrainMap database (<http://brainmap.org/>). This search aimed to retrieve VBM data related to other psychiatric disorders and neurological diseases showing significant gray matter reductions, excluding BD, MDD, and SZ results. Relevant data were identified from the VBM sector (Vanasse et al., 2018) of the BrainMap database using the following search algorithm:

• “Non-BD Non-MDD Non-SZ” query in BrainMap (via Sleuth v.3.0.4 software package; <https://www.brainmap.org/sleuth/>):

[Diagnosis matches either IS NOT bipolar disorder AND IS NOT major depressive disorder AND IS NOT schizophrenia] “+” [Experiments context is disease effects] “+” [Experiments contrast is gray matter] “+” [Observed changes match controls > patients].

Notably, the search interface of the Sleuth software systematically organizes BrainMap’s paradigm class entries. This feature facilitates the pooling of experiments of interest, rather than isolating them based on domain-specific keywords.

**eMethods 2.** Data selection

**Standard Criteria.** To be deemed eligible, VBM experiments were required to have been published in peer-reviewed English language journals and to statistically contrast gray matter volume and/or concentration between a clinical group and a cohort of healthy controls. Only experiments reporting whole-brain analyses accompanied by stereotactic coordinates (x, y, z) in Talairach or Montreal Neurological Institute (MNI) space were included. This selection process was implemented to mitigate potential biases associated with region-of-interest (ROI) analyses and to minimize spatial inaccuracies (Manuello, Costa, Cauda, & Liloia, 2022; Müller et al., 2018).

**Clinical Criteria.** For experiments involving individuals with BD, MDD, and SZ, the clinical groups were required to include participants with diagnoses established according to standardized DSM or ICD criteria. For experiments involving other psychiatric disorders and neurological diseases, only experiments categorized as "disease effects context" within the BrainMap VBM database were eligible. No restrictions were applied regarding the participants' age, sex, or diagnostic subtype. Experiments involving clinical conditions potentially associated with BD, MDD, or SZ were considered for inclusion, provided that none of the participants had a reported diagnosis of BD, MDD, or SZ. Experiments including medicated individuals were not excluded, given their significant representation in the literature (Chen et al., 2022; Gray, Müller, Eickhoff, & Fox, 2020; Liloia et al., 2021; Tahmasian et al., 2019). However, only experiments that reported between-group differences attributable to diagnosis were eligible; experiments primarily examining pharmacological treatment effects were excluded (Manuello et al., 2022; Müller et al., 2018).

**Technical Criteria.** To minimize possible redundancy due to overlapping patient groups, strict measures were adopted both within and between experiments. If multiple experiments by the same first author described the same clinical group and reported identical stereotactic coordinates, only the earliest publication was retained for analysis. In cases where multiple experiments were presented in a single publication, analyses involving the same patient group or its subgroups were excluded, ensuring that only experiments describing nonredundant clinical groups were included (Manuello et al., 2022). Duplicate findings across experiments by different first authors that analyzed the same participant cohort were identified and excluded through post hoc analyses using the GingerALE software package (v.3.0.2; <https://www.brainmap.org/software.html#GingerALE>).

**eMethods 3.** Anatomical likelihood estimation analysis

The anatomical likelihood estimation (ALE) is a quantitative voxel-based meta-analysis technique utilized to assess the neuroanatomical spatial convergence of findings through statistical comparisons across a selected sample of neuroimaging experiments from existing literature (Eickhoff, Bzdok, Laird, Kurth, & Fox, 2012; Eickhoff et al., 2016). In ALE, a three-dimensional Gaussian probability distribution is centered on each focus derived from experimental data (i.e., the local maximum coordinate of gray matter variation):

$$p\left( d \right)=\frac{1}{\sigma^{3}\sqrt{{(2\pi)}^{3}}}e^{- \frac{d^{2}}{2\sigma^{2}}}$$

Here, $d$ denotes the Euclidean distance between the voxel and the focus and $\sigma$ represents spatial uncertainty determined by the full-width half-maximum (FWHM):

$$\sigma=\frac{FWHM}{\sqrt{8In 2}}$$

The ALE procedure generates modeled activation (MA) maps for each selected experiment by combining Gaussian probability distributions derived from experimental foci (Laird et al., 2009). Subsequently, a final ALE map is constructed by combining all MA maps. Significant convergence is assessed by comparing these ALE scores to an empirical null distribution representing random spatial overlap between experiments while accounting for the fixed within-study distribution of foci. In this analysis, a random-effects approach is applied, which evaluates above-chance convergence across experiments (Turkeltaub et al., 2012); thus, the observed ALE scores are then tested against the null distribution to determine whether the spatial convergence of reported coordinates exceeds what would be expected by chance.

**eMethods 4.** Bayes fACtor mOdeliNg analysis

Bayes fACtor mOdeliNg (BACON) (Costa et al., 2021) was developed as a neuroimaging coordinate-based meta-analytic method to derive posterior probability distributions concerning variation in gray matter selectivity associated with a given brain disorder. This method integrates Bayes Factor (BF) (Kass & Raftery, 1995) calculations and unthresholded maps generated using the ALE method (Eickhoff et al., 2016).

The Bayes' theorem, represented in Equation 1, serves as the basis for expressing relative belief, also referred to as the BF:

$\frac{P\left( H_{0} | D \right)}{P\left( H_{1} | D \right)}=\frac{P\left( D | H_{0} \right)}{P\left( D | H_{1} \right)}\frac{P\left( H_{0} \right)}{P\left( H_{1} \right)}$ (1)

Where $D$denotes the measurement of gray matter variation at a given voxel derived from the ALE meta-analyses (i.e., disorder of interest map, and non-disorder of interest map); $H_{0}$ represents the hypothesis positing the occurrence of the disorder of interest at that voxel; and $H_{1}$ corresponds to the hypothesis asserting the occurrence of any other disorder (i.e., the negation of $H_{0}$). Using BACON, the probability that a measured variation ($D$) in each voxel of the brain indicates the presence of the disorder of interest (​$H_{0}$) can be computed. Due to the absence of prior knowledge about the hypotheses’ probabilities, they were assumed to have equal priors, a principle validated in prior studies (Cauda et al., 2024; Costa et al., 2021; Liloia et al., 2023). Therefore, the Bayes Factor ${BF}_{01}$ can be redefined as:

${BF}_{01}=\frac{P(D|H_{0})}{P\left( D | H_{1} \right)}$ (2)

By considering that the sum of posterior probabilities $H_{0}$​ and $H_{1}$ must equal 1, Equation 2 can be transformed as follows:

${BF}_{01}=\frac{P\left( H_{0} | D \right)}{1-P\left( H_{0} | D \right)}$ (3)

The Bayes Factor ${BF}_{01}$​ provides the degree of evidence for the two hypotheses: a value ${BF}_{01}>$ 1 indicates evidence favoring $H_{0}$, whereas ${BF}_{01}<$ 1 favors $H_{1}$. Inverting Equation 3, the posterior probability of the disorder of interest for each voxel can be expressed as follows:

$P\left( H_{0} | D \right)=\frac{BF_{01}}{BF_{01}+1}$ (4)

This formulation enables the direct computation of posterior probabilities for the hypothesis of interest using the Bayes Factor ${BF}_{01}$.

**eMethods 5.** Fail-safe analysis

The fail-safe approach is a statistical quantitative method utilized in medical and psychological meta-analyses (Orwin, 1983). In recent years, it has been adapted for use in the neuroimaging coordinate-based meta-analysis (CBMA) and domain to assess the robustness of ALE findings in relation to potential “file-drawer” publication biases (Acar, Seurinck, Eickhoff, & Moerkerke, 2018). This method operates under the assumption that unpublished experiments with opposing results exist and estimates how many such counter-evidence experiments can be added to a CBMA before its results are invalidated. Specifically, this approach incrementally introduces simulated noise (representing unreported experiments) into the meta-analytic dataset and assesses the stability of the original findings.

In our analysis, the fail-safe procedure was employed to address the possibility that BrainMap lacks a sufficient number of experiments with contradicting evidence. For this purpose, we applied the publicly available R script developed by Acar et al. (2018) (<https://github.com/NeuroStat/GenerateNull>). The pipeline for this method involves two primary phases: noise generation and robustness estimation.

During the noise generation phase, simulated noise experiments are created. To ensure the realism of the generated data, the algorithm is constrained by the distributions of x-y-z coordinates of gray matter variation and the number of clinical subjects reported in the included experiments. For example, if the experiments in the meta-analytic dataset of interest reported 1–30 significant foci of variation, the simulated noise experiments are generated within this same range. These constraints aim to ensure that the noise closely mimics plausible experimental data. Following this, the positions of the generated coordinates are randomized within the same gray matter mask used in the BACON framework. This process is performed independently for all datasets representing conditions outside the primary interest (i.e., Non-BD, Non-MDD, and Non-SZ).

Then, the simulated noise data and those of the original meta-analytic sample are combined and fed into the algorithm to perform the ALE (Eickhoff et al., 2016). This stage ensures that the analysis accounts for potential conflicting results absent from the datasets of no interest. The procedure involves multiple iterations for each dataset of no interest, increasing the proportion of simulated noise data incrementally in each run (i.e., 6%, 10%, and 30%). For example, at the 30% noise threshold of the primary analysis, the non-BD dataset is augmented with 115 simulated random experiments, the non-MDD dataset with 112 experiments, and the non-SZ dataset with 100 experiments. The ALE maps generated at each noise level are then integrated with the BACON framework to investigate the robustness of primary and additional findings. This is achieved by systematically evaluating the ability of BACON to maintain significant clusters of variation selectivity as noise levels increase. BACON maps are generated for every noise condition and subsequently compared to the original BACON map (generated before introducing simulated noise). This procedure offers an empirical assessment of how robust the BACON-derived results are to the potential influence of publication bias.

**eMethods 6.** Functional and behavioral analyses.

First, we assessed the normal pattern of task-based coactivation for each BACON-derived cluster of gray matter selectivity that survived our robustness analysis via meta-analytic connectivity modeling (MACM) (Laird et al., 2013; Robinson, Laird, Glahn, Lovallo, & Fox, 2010). This approach identifies brain areas that are consistently coactivating with a given ROI across a wide range of neuroimaging experiments. The analysis was performed using the BrainMap functional database (Fox & Lancaster, 2002; Laird et al., 2009), which focuses on functional MRI experiments of healthy participants involved in normal mapping task-based experiments without interventions or group comparisons. At the time of analysis (December 2024), 8,377 eligible experiments were included, each reporting results as stereotaxic coordinates. The MACM process began by identifying all the experiments within the database that reported at least one activation coordinate in the ROI selected. ALE meta-analysis was subsequently employed to evaluate convergence across the coordinates from these experiments. Significant coactivation outside the ROI was determined via ALE-based methods with a cluster-level familywise error correction threshold of P < 0.05 and a cluster-forming threshold of P < 0.001(Eickhoff et al., 2016).

Second, we used the Behavioral plugin (Lancaster et al., 2012) in Mango software (v.4.0) to statistically associate each task-based coactivation network identified via MACM with corresponding physiological mental processes by testing which functional neuroimaging task is more likely to activate a user-selected region of interest. This automated analysis is based on 9,400 functional MRI studies in healthy participants and classifies mental operations into five domains (i.e. cognition, perception, interoception, emotion, and action), which are further divided into 60 subdomains. A detailed taxonomy can be found at <https://brainmap.org/taxonomy/behaviors/>. A Bonferroni-corrected threshold at P < 0.05 was adopted to designate statistically significant behavioral associations (Lancaster et al., 2012; Liloia et al., 2021).

**eTable 1.** Experiments included in Bayes fACtor mOdeliNg analysis: BD dataset

| **First author** | **Year** | **PMID** | **Experimental group diagnosis**  **(details of the group)** | **Subjects** | **Controls** | **Foci** |
| --- | --- | --- | --- | --- | --- | --- |
| Adleman N E | 2012 | 22650379 | Bipolar Disorder | 55 | 66 | 2 |
| Adler C M | 2005 | 15922309 | Bipolar Disorder | 32 | 27 | 1 |
| Alonso-Lana S | 2016 | 27448153 | Bipolar Disorder | 33 | 28 | 1 |
| Altamura A C | 2018 | 29223329 | Bipolar Disorder | 46 | 56 | 4 |
| Altamura A C | 2018 | 29223329 | Bipolar Disorder | 62 | 56 | 10 |
| Amann B L | 2016 | 25968549 | Bipolar Disorder | 45 | 45 | 1 |
| Ambrosi E | 2013 | 23489395 | Bipolar Disorder | 20 | 21 | 8 |
| Brown G G | 2011 | 21924872 | Bipolar Disorder | 15 | 21 | 19 |
| Cai Y | 2015 | 25502401 | Bipolar Disorder | 23 | 23 | 2 |
| Castro-Fornieles J | 2018 | 28707138 | Bipolar Disorder | 15 | 70 | 3 |
| Chang M | 2018 | 29036668 | Bipolar Disorder | 86 | 156 | 12 |
| Chen X | 2007 | 17464719 | Bipolar Disorder | 24 | 25 | 1 |
| Chen L | 2018 | 30069426 | Bipolar Disorder | 43 | 47 | 2 |
| Cui L | 2011 | 21138758 | Bipolar Disorder | 24 | 36 | 7 |
| Doris A | 2004 | 15033185 | Bipolar Disorder | 11 | 16 | 34 |
| Eker C | 2014 | 24589068 | Bipolar Disorder | 28 | 30 | 3 |
| Farrow T F D | 2005 | 15993858 | Bipolar Disorder | 8 | 22 | 12 |
| Gao W | 2013 | 23477846 | Bipolar Disorder | 18 | 18 | 1 |
| Gao W | 2021 | 33714175 | Bipolar Disorder (Psychotic) | 28 | 19 | 7 |
| Gao W | 2021 | 33714175 | Bipolar Disorder (Non-Psychotic) | 26 |  | 4 |
| Gold A L | 2016 | 27871637 | Bipolar Disorder | 20 | 53 | 1 |
| Ha T H | 2009 | 19429131 | Bipolar Disorder | 23 | 23 | 19 |
| Ha T H | 2009 | 19429131 | Bipolar Disorder | 23 | 23 | 7 |
| Hajek T | 2013 | 22818781 | Bipolar Disorder | 19 | 18 | 1 |
| Hajek T | 2014 | 25074491 | Bipolar Disorder | 33 | 11 | 5 |
| Haldane M | 2008 | 18308812 | Bipolar Disorder | 44 | 44 | 12 |
| Haller S | 2011 | 21284917 | Bipolar Disorder | 19 | 47 | 1 |
| Hozer F | 2021 | 31983348 | Bipolar Disorder | 269 | 316 | 10 |
| Janssen J | 2008 | 18827723 | Bipolar Disorder | 20 | 51 | 1 |
| Keramatian K | 2021 | 32419481 | Bipolar Disorder | 61 | 43 | 1 |
| Kim D | 2013 | 23769608 | Bipolar Disorder | 49 | 50 | 3 |
| Lee J | 2017 | 28963911 | Bipolar Disorder | 21 | 21 | 5 |
| Lee DK | 2020 | 32379845 | Bipolar Disorder | 65 | 65 | 2 |
| Li M | 2011 | 21236649 | Bipolar Disorder | 24 | 36 | 5 |
| Li H | 2020 | 33023515 | Bipolar Disorder | 44 | 36 | 6 |
| Lochhead R A | 2004 | 15184034 | Bipolar Disorder | 11 | 31 | 1 |
| Lyoo I K | 2004 | 15013835 | Bipolar Disorder | 39 | 43 | 4 |
| Maggioni E | 2017 | 29136642 | Bipolar Disorder | 176 | 383 | 3 |
| Matsubara T | 2016 | 28030612 | Bipolar Disorder | 10 | 26 | 2 |
| McIntosh A M | 2004 | 15476683 | Bipolar Disorder | 19 | 49 | 2 |
| Metin B | 2023 | 37166512 | Bipolar Disorder | 37 | 27 | 3 |
| Minuzzi L | 2017 | 29232965 | Bipolar Disorder | 32 | 36 | 2 |
| Molina V | 2011 | 21188405 | Bipolar Disorder | 19 | 24 | 2 |
| Narita K | 2011 | 21115089 | Bipolar Disorder | 31 | 84 | 13 |
| Nugent A C | 2006 | 16256376 | Bipolar Disorder (Medicated) | 20 | 65 | 1 |
| Nugent A C | 2006 | 16256376 | Bipolar Disorder (Unmedicated) | 16 |  | 2 |
| Poletti A | 2016 | 26454335 | Bipolar Disorder | 206 | 136 | 1 |
| Redlich R | 2014 | 25188810 | Bipolar Disorder | 58 | 58 | 8 |
| Repple J | 2017 | 28239946 | Bipolar Disorder | 39 | 42 | 2 |
| Rocha-Rego V | 2013 | 23734914 | Bipolar Disorder (Cohort 1) | 26 | 26 | 5 |
| Rocha-Rego V | 2013 | 23734914 | Bipolar Disorder (Cohort 2) | 14 | 14 | 5 |
| Rossi R | 2013 | 23146251 | Bipolar Disorder | 14 | 40 | 20 |
| Sani G | 2016 | 26782273 | Bipolar Disorder | 78 | 78 | 7 |
| Saricicek A | 2015 | 26233321 | Bipolar Disorder | 28 | 29 | 1 |
| Sheperd A M | 2015 | 25268788 | Bipolar Disorder | 30 | 34 | 6 |
| Singh A | 2022 | 33846953 | Bipolar Disorder | 20 | 20 | 1 |
| Song J | 2015 | 26056452 | Bipolar Disorder | 44 | 35 | 3 |
| Song H | 2020 | 32571005 | Bipolar Disorder | 36 | 29 | 9 |
| Stanfield A C | 2009 | 19267696 | Bipolar Disorder | 66 | 66 | 2 |
| Sun N | 2020 | 32389612 | Bipolar Disorder | 30 | 31 | 7 |
| Tang LR | 2014 | 25218414 | Bipolar Disorder | 27 | 27 | 24 |
| Thiel K | 2024 | 38332015 | Bipolar Disorder (Type I) | 73 | 136 | 6 |
| Thiel K | 2024 | 38332015 | Bipolar Disorder (Type II) | 63 |  | 1 |
| Tost H | 2010 | 19419772 | Bipolar Disorder | 42 | 42 | 17 |
| Vai B | 2020 | 32238313 | Bipolar Disorder | 74 | 74 | 4 |
| Wang F | 2011 | 21666263 | Bipolar Disorder | 41 | 77 | 17 |
| Wang X | 2019 | 30905048 | Bipolar Disorder | 30 | 31 | 6 |
| Watson D R | 2012 | 22056751 | Bipolar Disorder | 24 | 24 | 1 |
| Xiao Q | 2020 | 32158006 | Bipolar Disorder | 21 | 18 | 3 |
| Xiao Q | 2020 | 32158006 | Bipolar Disorder | 19 | 18 | 3 |
| Yang Y | 2022 | 35757556 | Bipolar Disorder | 42 | 95 | 6 |
| Yatham L N | 2007 | 17988358 | Bipolar Disorder | 15 | 15 | 3 |
| Zhang Y N | 2021 | 33644197 | Bipolar Disorder | 13 | 20 | 4 |
|  |  |  | **TOTAL** | **2959** | **3602** | **420** |

**eTable 2.** Experiments included in Bayes fACtor mOdeliNg analysis: MDD dataset

| **First author** | **Year** | **PMID** | **Experimental group diagnosis (details of the group)** | **Subjects** | **Controls** | **Foci** |
| --- | --- | --- | --- | --- | --- | --- |
| Abe O | 2010 | 19959342 | Major depressive disorder | 21 | 42 | 9 |
| Alemany S | 2013 | 23433857 | Major depressive disorder | 12 | 21 | 4 |
| Arnone D | 2009 | 38553590 | Major depressive disorder | 25 | 35 | 4 |
| Arnone D | 2013 | 23128153 | Major depressive disorder | 39 | 66 | 5 |
| Begouiganan L | 2009 | 19071222 | Major depressive disorder | 21 | 21 | 6 |
| Cai Y | 2015 | 25502401 | Major depressive disorder | 23 | 23 | 1 |
| Camilleri | 2020 | 31884221 | Major depressive disorder | 85 | 86 | 1 |
| Chaney A | 2014 | 23900024 | Major depressive disorder | 37 | 46 | 2 |
| Chen M | 2020 | 32664021 | Major depressive disorder | 22 | 22 | 2 |
| Chen L | 2018 | 30069426 | Major depressive disorder | 36 | 47 | 3 |
| Cheng YQ | 2010 | 20594947 | Major depressive disorder | 68 | 68 | 1 |
| Dai N | 2020 | 32447184 | Major depressive disorder | 166 | 166 | 1 |
| Fang J | 2015 | 26125296 | Major depressive disorder | 20 | 18 | 7 |
| Frodl T | 2008 | 18838632 | Major depressive disorder | 38 | 30 | 65 |
| Gong Q | 2011 | 21134472 | Major depressive disorder | 23 | 42 | 9 |
| Grieve SM | 2013 | 24273717 | Major depressive disorder | 102 | 34 | 41 |
| Guo W | 2014 | 24863419 | Major depressive disorder (First-Episode) | 24 | 44 | 1 |
| Guo W | 2014 | 24863419 | Major depressive disorder (Recurrent) | 21 |  | 1 |
| Harada K | 2016 | 27344619 | Major depressive disorder | 45 | 61 | 6 |
| Harada K | 2018 | 29472854 | Major depressive disorder | 16 | 30 | 1 |
| Hellewell SC | 2019 | 31341158 | Major depressive disorder | 98 | 66 | 10 |
| Hwang JP | 2010 | 20430976 | Major depressive disorder | 70 | 26 | 3 |
| Igata N | 2017 | 28638109 | Major depressive disorder | 27 | 44 | 3 |
| Inkster B | 2011 | 20977527 | Major depressive disorder | 145 | 183 | 1 |
| Jiang X | 2021 | 33096331 | Major depressive disorder | 20 | 30 | 2 |
| Jiang Y | 2019 | 30149746 | Major depressive disorder | 20 | 20 | 4 |
| Jung J | 2014 | 25200096 | Major depressive disorder | 50 | 29 | 2 |
| Kandilarova S | 2019 | 31234950 | Major depressive disorder | 39 | 42 | 3 |
| Klauser P | 2015 | 25462395 | Major depressive disorder | 56 | 33 | 1 |
| Kim MJ | 2008 | 18930633 | Major depressive disorder | 22 | 25 | 4 |
| Kong L | 2014 | 24427263 | Major depressive disorder | 28 | 28 | 2 |
| Lai CH | 2014 | 24445133 | Major depressive disorder | 38 | 27 | 5 |
| Lai CH | 2015 | 26208214 | Major depressive disorder | 53 | 54 | 6 |
| Lee HY | 2011 | 21546094 | Major depressive disorder | 47 | 51 | 21 |
| Li CT | 2010 | 19931620 | Major depressive disorder | 25 | 25 | 3 |
| Li Y | 2019 | 31176187 | Major depressive disorder | 56 | 56 | 6 |
| Liu CH | 2014 | 24406440 | Major depressive disorder | 19 | 19 | 2 |
| Liu P | 2020 | 32421626 | Major depressive disorder (With Gastrointestinal Symptoms) | 36 | 27 | 1 |
| Liu P | 2020 | 32421626 | Major depressive disorder (Without Gastrointestinal Symptoms) | 22 |  | 1 |
| Liu P | 2019 | 31190826 | Major depressive disorder | 49 | 30 | 4 |
| Lu S | 2019 | 30831535 | Major depressive disorder | 30 | 48 | 1 |
| Machino A | 2012 | 25064808 | Major depressive disorder | 29 | 29 | 5 |
| Machino A | 2014 | 25064808 | Major depressive disorder | 29 | 29 | 1 |
| Mak AK | 2009 | 19596037 | Major depressive disorder | 17 | 17 | 11 |
| Meng Q | 2020 | 32631032 | Major depressive disorder | 159 | 53 | 1 |
| Mutschler I | 2019 | 25051163 | Major depressive disorder | 26 | 26 | 3 |
| Opel N | 2019 | 31304733 | Major depressive disorder | 506 | 358 | 3 |
| Opel N | 2016 | 26355299 | Major depressive disorder | 20 | 20 | 8 |
| Oudega M L | 2014 | 24686000 | Major depressive disorder | 55 | 23 | 5 |
| Ozalay O | 2016 | 27136662 | Major depressive disorder | 24 | 24 | 1 |
| Peng J | 2010 | 20466498 | Major depressive disorder | 22 | 30 | 18 |
| Perico CAM | 2011 | 21320250 | Major depressive disorder | 20 | 94 | 2 |
| Redlich R | 2014 | 25188810 | Major depressive disorder | 58 | 58 | 8 |
| Ribeiz S R I | 2013 | 24244606 | Major depressive disorder | 30 | 22 | 1 |
| Salvadore G | 2011 | 21073959 | Major depressive disorder | 58 | 107 | 6 |
| Scheuerecker J | 2010 | 20569645 | Major depressive disorder | 13 | 15 | 11 |
| Serra-Blasco M | 2013 | 23620451 | Major depressive disorder | 22 | 32 | 12 |
| Shad MU | 2012 | 22537357 | Major depressive disorder | 22 | 22 | 11 |
| Shah PJ | 1998 | 9828995 | Major depressive disorder | 20 | 20 | 6 |
| Shen Z | 2016 | 27668175 | Major depressive disorder | 147 | 130 | 6 |
| Smith GS | 2010 | 19173332 | Major depressive disorder | 16 | 13 | 6 |
| Soriano-Mas C | 2011 | 20875637 | Major depressive disorder | 70 | 40 | 1 |
| Stratmann M | 2014 | 25051163 | Major depressive disorder | 132 | 132 | 5 |
| Tae WS | 2015 | 19596030 | Major depressive disorder | 20 | 21 | 19 |
| Tang Y | 2007 | 17825533 | Major depressive disorder | 14 | 13 | 2 |
| Ueda I | 2016 | 26960194 | Major depressive disorder | 30 | 48 | 1 |
| van Eijndhoven P | 2013 | 23929204 | Major depressive disorder | 20 | 31 | 1 |
| van Tol MJ | 2010 | 20921116 | Major depressive disorder | 68 | 65 | 2 |
| van Tol MJ | 2014 | 24176247 | Major depressive disorder | 20 | 20 | 5 |
| Vasic N | 2008 | 18191459 | Major depressive disorder | 15 | 14 | 3 |
| Wagner G | 2011 | 20832482 | Major depressive disorder | 30 | 30 | 9 |
| Wang J | 2021 | 33505257 | Major depressive disorder | 36 | 27 | 1 |
| Xie C | 2012 | 22909988 | Major depressive disorder | 18 | 25 | 7 |
| Yang X | 2015 | 26425096 | Major depressive disorder | 50 | 50 | 1 |
| Yang S | 2017 | 28843886 | Major depressive disorder | 84 | 84 | 3 |
| Yang Y | 2022 | 35757556 | Major depressive disorder | 85 | 95 | 4 |
| Yang Y | 2020 | 32157476 | Major depressive disorder | 187 | 103 | 8 |
| Yuan Y | 2008 | 18550031 | Major depressive disorder | 19 | 16 | 3 |
| Zhang X | 2012 | 22129771 | Major depressive disorder | 33 | 32 | 1 |
| Zhang Y | 2020 | 33551870 | Major depressive disorder | 30 | 63 | 2 |
| Zhuo C | 2017 | 27766588 | Major depressive disorder | 45 | 48 | 1 |
| Zou K | 2010 | 19897176 | Major depressive disorder | 23 | 23 | 2 |
|  |  |  | **TOTAL** | **4116** | **3867** | **461** |

**eTable 3.** Experiments included in Bayes fACtor mOdeliNg analysis: SZ dataset

| **First Author** | **Year** | **PMID** | **Experimental group diagnosis (details of the group)** | **Subjects** | **Controls** | **Foci** |
| --- | --- | --- | --- | --- | --- | --- |
| Adamu M J | 2023 | 37275974 | Schizophrenia | 72 | 74 | 8 |
| Amann B L | 2016 | 25968549 | Schizophrenia | 45 | 18 | 9 |
| Ananth H | 2002 | 12202269 | Schizophrenia | 20 | 20 | 13 |
| Anderson V M | 2015 | 25716781 | Schizophrenia | 15 | 20 | 7 |
| Antonova E | 2005 | 16039619 | Schizophrenia | 45 | 40 | 4 |
| Asami T | 2012 | 21924364 | Schizophrenia | 33 | 36 | 6 |
| Bassitt D P | 2007 | 16960651 | Schizophrenia | 50 | 30 | 4 |
| Bonilha L | 2008 | 11040111 | Schizophrenia | 14 | 13 | 9 |
| Brown G G | 2011 | 21924872 | Schizophrenia | 17 | 21 | 9 |
| Cai J | 2022 | 35873260 | Schizophrenia | 60 | 40 | 2 |
| Cascella NG | 2010 | 20452187 | Schizophrenia | 19 | 90 | 12 |
| Chang M | 2016 | 27723806 | Schizophrenia | 60 | 71 | 9 |
| Chen Z | 2011 | 24443827 | Schizophrenia | 86 | 86 | 2 |
| Del Vecchio G | 2017 | 28290743 | Schizophrenia | 61 | 59 | 2 |
| Donohoe G | 2011 | 20934520 | Schizophrenia | 70 | 38 | 10 |
| Douaud G | 2007 | 17698497 | Schizophrenia | 25 | 25 | 23 |
| Egashira K | 2014 | 25358262 | Schizophrenia | 24 | 41 | 5 |
| Farrow T F D | 2005 | 15993858 | Schizophrenia | 25 | 22 | 19 |
| Ferri F | 2012 | 22361253 | Schizophrenia | 19 | 19 | 1 |
| Frascarelli M | 2023 | 36715578 | Schizophrenia | 16 | 19 | 1 |
| Garcia-Marti G | 2008 | 17716795 | Schizophrenia | 17 | 19 | 5 |
| Giuliani L | 2005 | 39753575 | Schizophrenia | 41 | 34 | 14 |
| Gou N | 2022 | 35492688 | Schizophrenia (Violent) | 31 | 43 | 5 |
| Gou N | 2022 | 35492688 | Schizophrenia (Non-Violent) | 39 |  | 5 |
| Guo X | 2013 | 24391807 | Schizophrenia | 30 | 33 | 6 |
| Guo W | 2015 | 26496253 | Schizophrenia | 49 | 46 | 6 |
| Guo F | 2019 | 31233895 | Schizophrenia | 33 | 33 | 21 |
| Ha T H | 2004 | 15664796 | Schizophrenia | 35 | 35 | 13 |
| Henze R | 2011 | 20572905 | Schizophrenia | 13 | 13 | 2 |
| Herold R | 2009 | 19016669 | Schizophrenia | 29 | 21 | 38 |
| Hidese S | 2018 | 29702462 | Schizophrenia | 83 | 130 | 8 |
| Hirao K | 2008 | 18774263 | Schizophrenia | 20 | 20 | 6 |
| Horacek J | 2011 | 21599563 | Schizophrenia | 44 | 56 | 13 |
| Hu M | 2013 | 23360727 | Schizophrenia | 55 | 59 | 1 |
| Huang P | 2015 | 26403064 | Schizophrenia | 18 | 26 | 3 |
| Janssen J | 2008 | 18827723 | Schizophrenia | 25 | 51 | 2 |
| Jayakumar PN | 2005 | 15866362 | Schizophrenia | 18 | 18 | 10 |
| Jiang Y | 2018 | 29668409 | Schizophrenia | 30 | 126 | 26 |
| Job DE | 2003 | 14511796 | Schizophrenia | 34 | 36 | 8 |
| Kasparek T | 2009 | 19647777 | Schizophrenia | 32 | 18 | 5 |
| Kawada R | 2009 | 19625009 | Schizophrenia | 26 | 26 | 13 |
| Kawasaki Y | 2007 | 17045492 | Schizophrenia | 30 | 30 | 9 |
| Kenneth Martin A | 2014 | 25453991 | Schizophrenia | 26 | 50 | 9 |
| Kim GW | 2017 | 28520743 | Schizophrenia | 22 | 22 | 4 |
| Kong L | 2015 | 25595222 | Schizophrenia | 22 | 20 | 17 |
| Koutsouleris N | 2007 | 18054834 | Schizophrenia | 175 | 177 | 34 |
| Kubicki M | 2002 | 12498745 | Schizophrenia | 16 | 18 | 1 |
| Lei W | 2015 | 26409573 | Schizophrenia | 88 | 44 | 1 |
| Lei W | 2019 | 31059954 | Schizophrenia | 14 | 32 | 1 |
| Li C | 2019 | 31377989 | Schizophrenia | 86 | 86 | 34 |
| Liao J | 2015 | 25937503 | Schizophrenia | 93 | 99 | 12 |
| Lui S | 2009 | 19168334 | Schizophrenia | 10 | 10 | 9 |
| Lui S | 2009 | 18981063 | Schizophrenia | 68 | 68 | 3 |
| Ma M | 2021 | 34354618 | Schizophrenia | 64 | 65 | 3 |
| Martì-Bonmatì L | 2007 | 17641373 | Schizophrenia | 21 | 10 | 8 |
| McDonald C | 2005 | 15863740 | Schizophrenia | 25 | 52 | 12 |
| McIntosh A M | 2004 | 8050090 | Schizophrenia | 26 | 49 | 4 |
| Meda S A | 2008 | 18378428 | Schizophrenia (First-Episode) | 34 | 34 | 37 |
| Meda S A | 2008 | 18378428 | Schizophrenia (Chronic) | 21 |  | 31 |
| Meisenzahl E M | 2008 | 18703313 | Schizophrenia (First-Episode) | 72 | 177 | 67 |
| Meisenzahl E M | 2008 | 18703313 | Schizophrenia (Chronic) | 93 |  | 48 |
| Molina V | 2010 | 20153145 | Schizophrenia | 30 | 40 | 3 |
| Molina V | 2011 | 21188405 | Schizophrenia | 17 | 41 | 11 |
| Moorhead T W | 2005 | 15110009 | Schizophrenia | 25 | 29 | 14 |
| Nakamura K | 2013 | 23508623 | Schizophrenia | 34 | 51 | 1 |
| Neugebauer K | 2019 | 31105606 | Schizophrenia | 18 | 19 | 21 |
| O'Daly O | 2007 | 17720459 | Schizophrenia | 28 | 32 | 7 |
| Oertel-Knochel V | 2012 | 22464726 | Schizophrenia | 31 | 37 | 8 |
| Ohnishi T | 2006 | 16330500 | Schizophrenia | 19 | 38 | 19 |
| Ortiz-Gil J | 2011 | 21727234 | Schizophrenia | 23 | 39 | 1 |
| Paillere-Martinot M L | 2001 | 11378311 | Schizophrenia | 20 | 20 | 9 |
| Poeppl T B | 2014 | 24423214 | Schizophrenia | 20 | 30 | 3 |
| Poletti S | 2016 | 26454335 | Schizophrenia | 96 | 136 | 8 |
| Pomarol-Clotet E | 2010 | 20065955 | Schizophrenia | 31 | 31 | 2 |
| Price G | 2010 | 19632338 | Schizophrenia | 41 | 47 | 4 |
| Qiu L | 2011 | 21991357 | Schizophrenia | 33 | 29 | 23 |
| Rametti G | 2010 | 20547448 | Schizophrenia | 23 | 23 | 1 |
| Ren W | 2013 | 23732942 | Schizophrenia | 100 | 100 | 6 |
| Rose E J | 2014 | 25044277 | Schizophrenia | 163 | 150 | 15 |
| Salgado-Pineda P | 2003 | 12814586 | Schizophrenia | 13 | 13 | 15 |
| Salgado-Pineda P | 2011 | 21095105 | Schizophrenia | 14 | 14 | 5 |
| Sans-Sansa B | 2013 | 23522907 | Schizophrenia | 31 | 59 | 5 |
| Sarro S | 2013 | 23222039 | Schizophrenia | 81 | 61 | 3 |
| Schaufelberger M S | 2007 | 18055927 | Schizophrenia | 62 | 94 | 6 |
| Schiffer B | 2010 | 20647266 | Schizophrenia | 12 | 14 | 11 |
| Schuster C | 2012 | 21205677 | Schizophrenia | 27 | 40 | 12 |
| Shapleske J | 2002 | 12427683 | Schizophrenia | 72 | 32 | 9 |
| Shen C L | 2023 | 36604437 | Schizophrenia (15-Year Group) | 36 | 72 | 3 |
| Shen C L | 2023 | 36604437 | Schizophrenia (25-Year Group) | 39 | 78 | 6 |
| Sheng J | 2013 | 23769260 | Schizophrenia | 33 | 41 | 15 |
| Sigmundsson T | 2001 | 11156806 | Schizophrenia | 27 | 27 | 5 |
| Singh S | 2014 | 24562867 | Schizophrenia | 14 | 14 | 3 |
| Singh S | 2015 | 25963262 | Schizophrenia | 14 | 14 | 22 |
| Smesny S | 2010 | 20478385 | Schizophrenia (First-Episode) | 13 | 25 | 4 |
| Smesny S | 2010 | 20478385 | Schizophrenia (Chronic) | 11 |  | 16 |
| Spalthoff R | 2018 | 30049600 | Schizophrenia | 51 | 102 | 6 |
| Suzuki M | 2002 | 11955962 | Schizophrenia | 45 | 42 | 5 |
| Tan S | 2015 | 26406464 | Schizophrenia | 18 | 17 | 5 |
| Tang J | 2012 | 22802957 | Schizophrenia | 29 | 34 | 1 |
| Tian L | 2011 | 22174900 | Schizophrenia | 30 | 29 | 50 |
| Tomelleri L | 2009 | 19717283 | Schizophrenia | 45 | 79 | 2 |
| Torres U S | 2016 | 27354958 | Schizophrenia (Chronic) | 99 | 151 | 15 |
| Torres U S | 2016 | 27354958 | Schizophrenia (First-Episode) | 62 |  | 5 |
| Tregellas J R | 2007 | 17336502 | Schizophrenia | 64 | 32 | 9 |
| Van Ascsche L | 2024 | 37577955 | Schizophrenia | 35 | 36 | 2 |
| van Tol MJ | 2014 | 25061563 | Schizophrenia | 51 | 51 | 7 |
| Venkatasubramanian G | 2010 | 20174515 | Schizophrenia | 30 | 27 | 14 |
| Voets N L | 2008 | 18793730 | Schizophrenia | 25 | 25 | 20 |
| Wang J | 2017 | 29166884 | Schizophrenia | 18 | 21 | 3 |
| Watson DR | 2012 | 22056751 | Schizophrenia | 25 | 25 | 2 |
| Whitford T J | 2005 | 16677830 | Schizophrenia | 31 | 30 | 12 |
| Wilke M | 2001 | 11304078 | Schizophrenia | 48 | 48 | 11 |
| Witthaus H | 2009 | 19616415 | Schizophrenia | 23 | 29 | 7 |
| Wolf R C | 2008 | 18434103 | Schizophrenia | 28 | 14 | 12 |
| Wu H | 2022 | 36221050 | Schizophrenia | 74 | 53 | 6 |
| Xu L | 2009 | 18266214 | Schizophrenia | 120 | 120 | 55 |
| Yamada M | 2007 | 17240165 | Schizophrenia | 20 | 20 | 6 |
| Yang ZY | 2019 | 31629132 | Schizophrenia | 37 | 28 | 12 |
| Yang Y | 2022 | 35757556 | Schizophrenia | 70 | 95 | 4 |
| Yoshihara Y | 2008 | 19102744 | Schizophrenia | 18 | 18 | 1 |
| Zhang Y | 2015 | 26281967 | Schizophrenia | 37 | 30 | 1 |
| Zhao X | 2022 | 35751735 | Schizophrenia | 52 | 29 | 7 |
| Zhuo C | 2017 | 28143464 | Schizophrenia | 95 | 93 | 10 |
|  |  |  | **TOTAL** | **5015** | **5436** | **1295** |

**eTable 4.** Experiments included in Bayes fACtor mOdeliNg analysis: BrainMap psychiatric disorders dataset.

| **First author** | **Year** | **BrainMap ID** | **Experimental group diagnosis (details of the group)** | **Subjects** | **Controls** | **Foci** |
| --- | --- | --- | --- | --- | --- | --- |
| Abell F | 1999 | 12060002 | Autism Spectrum Disorder | 15 | 15 | 3 |
| Ahmed F | 2012 | 14110027 | Post Traumatic Stress Disorder | 21 | 32 | 3 |
| Ahrendts J | 2011 | 13100086 | Attention Deficit Hyperactivity Disorder | 31 | 31 | 2 |
| Altena E | 2010 | 11040009 | Insomnia Disorder | 24 | 13 | 3 |
| Amianto F | 2021 | 21080051 | Bulimia Nervosa | 13 | 14 | 2 |
| Asami T | 2009 | 11040040 | Panic Disorder | 24 | 24 | 14 |
| Bertsch K | 2013 | 15090036 | Antisocial Personality Disorder (With Borderline Personality Disorder) | 13 | 14 | 20 |
| Bertsch K | 2013 | 15090036 | Antisocial Personality Disorder (Without Borderline Personality Disorder) | 12 |  | 30 |
| Boddaert N | 2004 | 8060159 | Autism Spectrum Disorder | 21 | 12 | 4 |
| Boghi A | 2011 | 13100004 | Anorexia Nervosa | 21 | 27 | 19 |
| Bonath B | 2018 | 19120002 | Attention Deficit Hyperactivity Disorder | 18 | 18 | 12 |
| Bossini L | 2017 | 21120112 | Post Traumatic Stress Disorder | 19 | 19 | 5 |
| Brambati S M | 2004 | 8050024 | Specific Learning Disorder | 10 | 11 | 9 |
| Brieber S | 2007 | 10010005 | [Attention Deficit Hyperactivity Disorder](https://www.nhs.uk/conditions/attention-deficit-hyperactivity-disorder-adhd/) | 15 | 15 | 9 |
| Brieber S | 2007 | 10010005 | Autism Spectrum Disorder | 15 | 15 | 6 |
| Brooks S J | 2011 | 13100007 | Anorexia Nervosa | 14 | 21 | 5 |
| Brooks S J | 2013 | 18100012 | Binge Eating Disorder | 59 | 97 | 1 |
| Brenneis C | 2005 | 8050031 | Narcolepsy | 12 | 12 | 3 |
| Brown W E | 2001 | 8050033 | Specific Learning Disorder | 16 | 14 | 8 |
| Brunner R | 2010 | 11040043 | Borderline Personality Disorder | 20 | 20 | 3 |
| Budhiraja M | 2017 | 21090085 | Conduct Disorder | 31 | 25 | 4 |
| Cai J | 2018 | 19120003 | Autism Spectrum Disorder | 38 | 27 | 3 |
| Carmona S | 2005 | 8060164 | Attention Deficit Hyperactivity Disorder | 25 | 25 | 17 |
| Castro-Fornieles J | 2009 | 9050023 | Anorexia Nervosa | 12 | 9 | 3 |
| Castro-Manglano P D | 2011 | 13100191 | Schizoaffective Disorder | 18 | 20 | 10 |
| Chanraud S | 2007 | 8050039 | Substance Use Disorder | 31 | 28 | 10 |
| Chanraud S | 2009 | 11040189 | Substance Use Disorder | 24 | 24 | 14 |
| Chao L L | 2012 | 14110028 | Post Traumatic Stress Disorder (Medicated) | 17 | 20 | 3 |
| Chao L L | 2012 | 14110028 | Post Traumatic Stress Disorder (Unmedicated) | 15 |  | 2 |
| Chen S | 2006 | 8050042 | Post Traumatic Stress Disorder | 12 | 12 | 4 |
| Chen S | 2009 | 11040190 | Post Traumatic Stress Disorder | 12 | 12 | 3 |
| Chen Y | 2012 | 14110029 | Post Traumatic Stress Disorder | 10 | 10 | 1 |
| Cheng B | 2015 | 16010005 | Post Traumatic Stress Disorder | 30 | 30 | 3 |
| Cheng Y | 2011 | 12060004 | Autism Spectrum Disorder (Asperger's Syndrome) | 11 | 25 | 13 |
| Cheng Y | 2011 | 12060004 | Autism Spectrum Disorder (Primary Autism) | 12 |  | 3 |
| Corbo V | 2005 | 8050044 | Post Traumatic Stress Disorder | 14 | 14 | 5 |
| Craig M C | 2007 | 11080274 | Autism Spectrum Disorder | 14 | 19 | 5 |
| D'Agata F | 2015 | 21080069 | Anorexia Nervosa | 21 | 17 | 7 |
| D'Agata F | 2015 | 21080069 | Bulimia Nervosa | 18 |  | 2 |
| Dalwani M | 2011 | 15090038 | Conduct Disorder | 25 | 19 | 4 |
| de Oliveira-Souza R | 2008 | 11040185 | Antisocial Personality Disorder | 15 | 15 | 22 |
| Eckart C | 2011 | 13100106 | Post Traumatic Stress Disorder | 20 | 13 | 5 |
| Ecker C | 2010 | 13100108 | Autism Spectrum Disorder | 22 | 22 | 14 |
| Ecker C | 2012 | 13100107 | Autism Spectrum Disorder | 89 | 89 | 11 |
| Eckert M A | 2005 | 8060170 | Specific Learning Disorder | 13 | 13 | 5 |
| Fahim C | 2012 | 20060011 | Oppositional Defiant Disorder | 18 | 20 | 1 |
| Fairchild G | 2011 | 15090039 | Conduct Disorder (Early-Onset) | 36 | 27 | 6 |
| Fairchild G | 2011 | 15090039 | Conduct Disorder (Adolescent-Onset) | 27 |  | 4 |
| Foster N E | 2015 | 19120005 | Autism Spectrum Disorder | 38 | 46 | 1 |
| Freitag C M | 2008 | 19120006 | Autism Spectrum Disorder | 15 | 15 | 1 |
| Friedrich H C | 2012 | 13100020 | Anorexia Nervosa (Without Weight-Restored) | 12 | 14 | 8 |
| Friedrich H C | 2012 | 13100020 | Anorexia Nervosa Patients (With Weight-Restored) | 13 |  | 2 |
| Galandra C | 2018 | 21090079 | Substance Use Disorder | 23 | 18 | 16 |
| Gao Y | 2021 | 21090080 | Conduct Disorder | 34 | 34 | 3 |
| Gaudio S | 2011 | 13100024 | Anorexia Nervosa | 15 | 16 | 3 |
| Gilbert A R | 2008 | 9050032 | Obsessive Compulsive Disorder | 25 | 20 | 7 |
| Gregory S | 2012 | 15090040 | Psychopathy | 17 | 22 | 18 |
| Greimel E | 2013 | 19120006 | Autism Spectrum Disorder | 47 | 51 | 4 |
| Guggenmos M | 2017 | 21020006 | Substance Use Disorder | 119 | 97 | 22 |
| Hakamata Y | 2007 | 11040195 | Post Traumatic Stress Disorder | 14 | 70 | 1 |
| He N | 2015 | 19120007 | Attention Deficit Hyperactivity Disorder | 37 | 35 | 4 |
| Herringa R | 2012 | 14110033 | Post Traumatic Stress Disorder | 13 | 28 | 4 |
| Hoeft F | 2007 | 13100121 | Specific Learning Disorder | 19 | 19 | 6 |
| Huebner T | 2008 | 11040094 | Conduct Disorder | 23 | 23 | 3 |
| Hyde K L | 2010 | 11040196 | Autism Spectrum Disorder | 15 | 13 | 3 |
| Iannaccone R | 2015 | 19120008 | Attention Deficit Hyperactivity Disorder | 18 | 18 | 3 |
| Jagger-Rickels A C | 2018 | 19120001 | Attention Deficit Hyperactivity Disorder | 41 | 32 | 15 |
| Jagger-Rickels A C | 2018 | 19120001 | Specific Learning Disorder | 17 |  | 12 |
| Jang D P | 2007 | 11080278 | Substance Use Disorder | 20 | 20 | 12 |
| Johnston B A | 2014 | 19120009 | Attention Deficit Hyperactivity Disorder | 34 | 34 | 12 |
| Joos A | 2010 | 10060042 | Anorexia Nervosa | 12 | 18 | 7 |
| Kappel V | 2014 | 19120010 | Attention Deficit Hyperactivity Disorder (Adults) | 16 | 20 | 5 |
| Kappel V | 2014 | 19120010 | Attention Deficit Hyperactivity Disorder (Children) | 14 |  | 3 |
| Karlsson H | 2014 | 18090003 | Binge Eating Disorder | 23 | 22 | 7 |
| Kasai K | 2008 | 12070015 | Post Traumatic Stress Disorder | 18 | 23 | 7 |
| Kaufman | 2013 | 21070046 | Autism Spectrum Disorder | 10 | 10 | 1 |
| Kaufmann C | 2002 | 8050076 | Narcolepsy | 12 | 32 | 19 |
| Kawasaki Y | 2004 | 11080309 | Schizotypal Disorder | 25 | 50 | 4 |
| Ke X | 2008 | 11040048 | Autism Spectrum Disorder | 17 | 15 | 1 |
| Kim S J | 2009 | 11040050 | Narcolepsy | 17 | 17 | 29 |
| Kobel M | 2010 | 13100130 | Attention Deficit Hyperactivity Disorder | 14 | 12 | 1 |
| Koprivova J | 2009 | 11040051 | Obsessive Compulsive Disorder | 14 | 15 | 30 |
| Kronbichler M | 2008 | 11080295 | Specific Learning Disorder | 13 | 15 | 9 |
| Kumar U | 2017 | 19120011 | Attention Deficit Hyperactivity Disorder | 18 | 18 | 4 |
| Kurth F | 2011 | 12060005 | Autism Spectrum Disorder | 52 | 52 | 1 |
| Kwon H | 2004 | 8050081 | Autism Spectrum Disorder (Asperger's Syndrome) | 11 | 13 | 3 |
| Lai C H | 2012 | 16080066 | Panic Disorder | 30 | 21 | 4 |
| Lai C H | 2015 | 17050006 | Panic Disorder | 53 | 54 | 2 |
| Leutgeb V | 2016 | 22030018 | Borderline Personality Disorder | 20 | 19 | 3 |
| Li L | 2006 | 8050084 | Post Traumatic Stress Disorder | 12 | 12 | 4 |
| Li X | 2015 | 19120024 | Attention Deficit Hyperactivity Disorder | 30 | 30 | 2 |
| Liao M | 2013 | 20070026 | Anxiety Disorder | 26 | 25 | 1 |
| Lim L | 2013 | 19120012 | Attention Deficit Hyperactivity Disorder | 29 | 29 | 3 |
| Ludolph A G | 2006 | 8060209 | Tourette Syndrome | 14 | 15 | 2 |
| Ma Z | 2019 | 21110103 | Generalized Anxiety Disorder | 15 | 28 | 2 |
| Maier S | 2016 | 19120015 | Attention Deficit Hyperactivity Disorder | 131 | 95 | 1 |
| Mannerkoski M K | 2009 | 11040199 | Intellectual Disablility | 26 | 39 | 2 |
| Massana G | 2003 | 8050087 | Panic Disorder | 18 | 18 | 1 |
| Massimo L | 2009 | 11040200 | Persistent Depressive Disorder | 9 | 27 | 22 |
| Massimo L | 2009 | 11040200 | Histrionic Personality Disorder | 5 |  | 24 |
| Matsumoto R | 2010 | 13100139 | Obsessive Compulsive Disorder | 16 | 32 | 4 |
| McAlonan G M | 2007 | 13100043 | Attention Deficit Hyperactivity Disorder | 28 | 31 | 8 |
| McAlonan G M | 2005 | 12060007 | Autism Spectrum Disorder | 17 | 17 | 13 |
| McAlonan G M | 2008 | 11040026 | Autism Spectrum Disorder (High Functioning Autism) | 17 | 55 | 8 |
| McAlonan G M | 2008 | 11040026 | Autism Spectrum Disorder (Asperger's Syndrome) | 16 |  | 4 |
| McAlonan G M | 2002 | 12060006 | Autism Spectrum Disorder | 17 | 24 | 9 |
| Mengotti P | 2011 | 12060001 | Autism Spectrum Disorder (Children) | 7 | 7 | 2 |
| Mengotti P | 2011 | 12060001 | Autism Spectrum Disorder | 20 | 22 | 2 |
| Milham M P | 2005 | 8050094 | Anxiety Disorder | 17 | 34 | 6 |
| Moon C | 2014 | 21110106 | Generalized Anxiety Disorder | 13 | 9 | 5 |
| Moon C | 2015 | 21110105 | Generalized Anxiety Disorder | 17 | 17 | 5 |
| Moon C | 2018 | 21060016 | Obsessive Compulsive Disorder | 18 | 18 | 3 |
| Moorhead T W | 2005 | 8090183 | Specific Learning Disorder | 18 | 29 | 9 |
| Muller S | 2013 | 19120018 | Autism Spectrum Disorder | 12 | 12 | 10 |
| Muller-Vahl K R | 2009 | 11040201 | Tourette Syndrome | 19 | 20 | 14 |
| Na K S | 2013 | 17050007 | Panic Disorder (Without Agoraphobia) | 22 | 22 | 2 |
| Na K S | 2013 | 17050007 | Panic Disorder (With Agoraphobia) | 12 |  | 7 |
| Nardo D | 2010 | 11050255 | Post Traumatic Stress Disorder | 21 | 22 | 5 |
| Niedtfeld I | 2013 | 14110036 | Borderline Personality Disorder | 60 | 60 | 2 |
| O'Doherty D C M | 2017 | 21120115 | Post Traumatic Stress Disorder | 25 | 25 | 26 |
| Overmeyer S | 2001 | 13100054 | Attention Deficit Hyperactivity Disorder | 18 | 16 | 9 |
| Pannacciulli N | 2006 | 8050103 | Binge Eating Disorder | 24 | 36 | 7 |
| Pujol J | 2004 | 13100059 | Obsessive Compulsive Disorder | 72 | 72 | 3 |
| Ramos-Quiroga J A | 2016 | 19120017 | Attention Deficit Hyperactivity Disorder | 44 | 44 | 3 |
| Riva D | 2011 | 12060008 | Autism Spectrum Disorder | 21 | 21 | 13 |
| Rocha-Rego V | 2012 | 14110031 | Post Traumatic Stress Disorder | 16 | 16 | 2 |
| Roman-Urrestarazu A | 2016 | 19120019 | Attention Deficit Hyperactivity Disorder | 49 | 34 | 1 |
| Rossi R | 2012 | 17050008 | Borderline Personality Disorder | 26 | 26 | 39 |
| Salmond C H | 2005 | 8050117 | Autism Spectrum Disorder | 14 | 13 | 2 |
| Salmond C H | 2007 | 12060009 | Autism Spectrum Disorder (Low Functioning) | 9 | 9 | 6 |
| Sasayama D | 2010 | 13100160 | Attention Deficit Hyperactivity Disorder | 10 | 17 | 9 |
| Schiffer B | 2007 | 8050120 | Pedophilic Disorder | 18 | 24 | 2 |
| Schwartz D L | 2010 | 10030032 | Substance Use Disorder | 61 | 44 | 4 |
| Seidman L J | 2019 | 19120020 | Attention Deficit Hyperactivity Disorder | 74 | 54 | 3 |
| Sethi A | 2017 | 19120021 | Attention Deficit Hyperactivity Disorder | 30 | 30 | 2 |
| Shott M E | 2015 | 18100011 | Binge Eating Disorder | 18 | 24 | 8 |
| Silani G | 2005 | 8060227 | Specific Learning Disorder | 32 | 32 | 1 |
| Sobanski T | 2010 | 11040145 | Panic Disorder | 17 | 17 | 2 |
| Spencer M D | 2006 | 8050129 | Intellectual Disablility | 63 | 72 | 2 |
| Steinbrink C | 2008 | 9050065 | Specific Learning Disorder | 7 | 7 | 2 |
| Stevens M C | 2019 | 19120016 | Attention Deficit Hyperactivity Disorder | 24 | 24 | 1 |
| Strawn J R | 2013 | 21110107 | Generalized Anxiety Disorder | 15 | 28 | 2 |
| Suchan B | 2010 | 11040077 | Anorexia Nervosa | 15 | 15 | 2 |
| Sui S G | 2010 | 13100070 | Post Traumatic Stress Disorder | 11 | 12 | 11 |
| Szesko P R | 2008 | 11040103 | Obsessive Compulsive Disorder | 37 | 26 | 3 |
| Tanabe J | 2009 | 9050067 | Substance Use Disorder | 19 | 20 | 1 |
| Tavanti M | 2012 | 13100174 | Post Traumatic Stress Disorder | 25 | 25 | 26 |
| Thomaes K | 2010 | 13100176 | Post Traumatic Stress Disorder | 31 | 28 | 5 |
| Tiihonen J | 2008 | 9050069 | Psychopathy | 12 | 25 | 2 |
| Tiihonen J | 2008 | 9050069 | Antisocial Personality Disorder | 26 |  | 31 |
| Toal F | 2010 | 12060010 | Autism Spectrum Disorder | 65 | 33 | 3 |
| Togao O | 2010 | 13100178 | Obsessive Compulsive Disorder | 23 | 26 | 6 |
| Tomoda A | 2009 | 11050264 | Dissociative Identity Disorder | 23 | 14 | 1 |
| Uchida R R | 2008 | 9050085 | Panic Disorder | 19 | 20 | 1 |
| Valente A A Jr | 2005 | 8060229 | Obsessive Compulsive Disorder | 19 | 15 | 2 |
| Van den Heuvel O A | 2009 | 11040186 | Obsessive Compulsive Disorder | 55 | 50 | 5 |
| van Tol M J | 2010 | 13100073 | Anxiety Disorder | 68 | 65 | 1 |
| van Wingen G | 2013 | 19120027 | Attention Deficit Hyperactivity Disorder (Without Cocaine Dependence) | 14 | 15 | 2 |
| van Wingen G | 2013 | 19120027 | Attention Deficit Hyperactivity Disorder (With Cocaine Dependence) | 10 |  | 2 |
| Villemointeix T | 2015 | 19120022 | Attention Deficit Hyperactivity Disorder (Met158-Carriers) | 28 | 15 | 1 |
| Villemointeix T | 2015 | 19120022 | Attention Deficit Hyperactivity Disorder (Val158 homozygotes) | 10 | 25 | 1 |
| Wang H | 2022 | 22020014 | Autism Spectrum Disorder (Pediatric) | 24 | 19 | 2 |
| Wang H | 2022 | 22020014 | Autism Spectrum Disorder (Adult) | 10 | 13 | 1 |
| Wang J | 2007 | 13100076 | Attention Deficit Hyperactivity Disorder | 12 | 12 | 4 |
| Wang L | 2019 | 19120013 | Attention Deficit Hyperactivity Disorder | 30 | 25 | 3 |
| Watkins K E | 2002 | 8060231 | Language Disorder | 10 | 17 | 10 |
| Yoneyama E | 2003 | 8090206 | Schizotypal Personality Disorder | 14 | 28 | 3 |
| Yang Q | 2018 | 19120025 | Autism Spectrum Disorder | 16 | 16 | 3 |
| Yoo H K | 2005 | 8050150 | Panic Disorder | 18 | 18 | 7 |
| Yoo S Y | 2008 | 11040184 | Obsessive Compulsive Disorder (Female) | 24 | 24 | 4 |
| Yoo S Y | 2009 | 11040185 | Obsessive Compulsive Disorder (Male) | 47 | 47 | 5 |
| Zhang J | 2011 | 13100080 | Post Traumatic Stress Disorder | 10 | 10 | 3 |
| Zhang J | 2018 | 21080075 | Conduct Disorder | 60 | 60 | 3 |
| Zhang X | 2018 | 21120121 | Post Traumatic Stress Disorder | 35 | 36 | 1 |
| Zhao Y | 2019 | 19120026 | Attention Deficit Hyperactivity Disorder | 36 | 36 | 6 |
| **TOTAL** | | | | **4246** | **4234** | **1137** |

**eTable 5.** Experiments included in Bayes fACtor mOdeliNg analysis: BrainMap psychiatric disorders and neurological diseases dataset.

| **First author** | **Year** | **BrainMap ID** | **Experimental group diagnosis (details of the group)** | **Subjects** | **Controls** | **Foci** |
| --- | --- | --- | --- | --- | --- | --- |
| Abele M | 2007 | 11040215 | Spinocerebellar Ataxia | 14 | 14 | 1 |
| Abell F | 1999 | 12060002 | Autism Spectrum Disorder | 15 | 15 | 3 |
| Adlam A L R | 2006 | 8060152 | Primary Progressive Aphasia | 7 | 22 | 6 |
| Agosta F | 2007 | 8060153 | Amyotrophic Lateral Sclerosis | 25 | 18 | 4 |
| Agosta F | 2010 | 13100084 | Progressive Supranuclear Palsy | 20 | 24 | 16 |
| Agosta F | 2011 | 13100085 | Alzheimer's Disease | 23 | 15 | 32 |
| Agosta F | 2011 | 13100085 | Mild Cognitive Impairment | 15 | 0 | 1 |
| Agosta F | 2013 | 23090001 | Parkinson's Disease | 54 | 42 | 4 |
| Agostini A | 2013 | 16030040 | Chronic Pain (Crohn's Disease) | 18 | 18 | 4 |
| Ahmed F | 2012 | 14110027 | Post Traumatic Stress Disorder | 21 | 32 | 3 |
| Ahmed R M | 2019 | 22050051 | Alzheimer's Disease | 16 | 19 | 9 |
| Ahmed R M | 2019 | 22050051 | Frontotemporal Dementia | 28 |  | 13 |
| Ahrendts J | 2011 | 13100086 | Attention Deficit Hyperactivity Disorder | 31 | 31 | 2 |
| Alcauter S | 2011 | 11080265 | Spinocerebellar Ataxia | 9 | 9 | 65 |
| Altena E | 2010 | 11040009 | Insomnia Disorder | 24 | 13 | 3 |
| Amianto F | 2021 | 21080051 | Bulimia Nervosa | 13 | 14 | 2 |
| Antonini G | 2004 | 8050005 | Myotonic Dystrophy | 22 | 22 | 21 |
| As-Sanie S | 2012 | 13110201 | Chronic Pain (With Endometriosis) | 17 | 17 | 7 |
| As-Sanie S | 2012 | 13110201 | Chronic Pain (Without Endometriosis) | 15 | 14 | 4 |
| As-Sanie S | 2012 | 13110201 | Chronic Pain | 6 | 12 | 1 |
| Asami T | 2009 | 11040040 | Panic Disorder | 24 | 24 | 14 |
| Ash S | 2011 | 13100089 | Lewy Body Dementia | 11 | 16 | 43 |
| Ash S | 2009 | 13100090 | Progressive Non-Fluent Aphasia | 6 | 10 | 3 |
| Ash S | 2009 | 13100090 | Semantic Dementia (Without Social/Executive Disorder) | 7 |  | 3 |
| Ash S | 2009 | 13100090 | Semantic Dementia (With Social/Executive Disorder) | 9 |  | 7 |
| Ash S | 2016 | 22060083 | Frontotemporal Dementia | 54 | 27 | 2 |
| Aubert-Broche B | 2011 | 16010003 | Multiple Sclerosis | 30 | 29 | 4 |
| Audoin B | 2006 | 8050006 | Multiple Sclerosis | 21 | 10 | 1 |
| Audoin B | 2010 | 10060033 | Clinical Isolated Syndrome | 62 | 37 | 26 |
| Audoin B | 2004 | 16020008 | Multiple Sclerosis | 18 | 18 | 10 |
| Audoin B | 2007 | 16030011 | Multiple Sclerosis | 38 | 45 | 6 |
| Aydin K | 2009 | 11040187 | Subacute Sclerosing Panencephalitis | 17 | 30 | 11 |
| Baez S | 2016 | 22060073 | Frontotemporal Dementia | 26 | 23 | 8 |
| Baez S | 2016 | 22070092 | Frontotemporal Dementia | 21 | 19 | 5 |
| Baez S | 2019 | 22050074 | Frontotemporal Dementia | 16 | 22 | 19 |
| Bai F | 2008 | 20100037 | Mild Cognitive Impairment | 20 | 20 | 7 |
| Baldeweg T | 2006 | 11080292 | Sickle Cell Disease Patients (With Lesions) | 16 | 31 | 1 |
| Baldeweg T | 2006 | 11080292 | Sickle Cell Disease Patients (Without Lesions) | 20 |  | 1 |
| Barad M J | 2013 | 16030041 | Chronic Pain (Complex Regional Pain Syndrome) | 15 | 15 | 9 |
| Barbeau E | 2008 | 11040158 | Mild Cognitive Impairment (Normal DMS48 Performance) | 12 | 28 | 17 |
| Barbeau E | 2008 | 11040158 | Mild Cognitive Impairment (Impaired DMS48 Performance) | 16 |  | 13 |
| Baron J C | 2001 | 8050007 | Alzheimer's Disease | 19 | 16 | 14 |
| Baxter L C | 2006 | 8050011 | Alzheimer's Disease | 15 | 15 | 6 |
| Beacher F D | 2009 | 11040065 | Neurocardiogenic Syncope | 18 | 19 | 3 |
| Bell-McGinty S | 2005 | 8060155 | Mild Cognitive Impairment (Converters) | 9 | 47 | 7 |
| Bell-McGinty S | 2005 | 8060155 | Mild Cognitive Impairment (Non-Converters) | 28 |  | 10 |
| Belton E | 2003 | 8060156 | Dyspraxia (Verbal & Orofacial) | 10 | 17 | 9 |
| Berlingeri M | 2008 | 12070013 | Alzheimer's Disease | 21 | 22 | 16 |
| Bernasconi N | 2004 | 8050013 | Epilepsy (Temporal Lobe Left-Sided) | 45 | 47 | 26 |
| Bernasconi N | 2004 | 8050013 | Epilepsy (Temporal Lobe Right-Sided) | 40 |  | 13 |
| Bertsch K | 2013 | 15090036 | Antisocial Personality Disorder (With Borderline Personality Disorder) | 13 | 14 | 20 |
| Bertsch K | 2013 | 15090036 | Antisocial Personality Disorder (Without Borderline Personality Disorder) | 12 |  | 30 |
| Beyer M K | 2007 | 8050016 | Parkinson's Disease | 16 | 20 | 18 |
| Bezdicek O | 2017 | 23090002 | Parkinson's Disease | 19 | 27 | 1 |
| Bitter T | 2010 | 10060034 | Anosmia | 17 | 17 | 35 |
| Bitter T | 2010 | 11040084 | Hyposmia (Post-Infectious) | 24 | 43 | 15 |
| Bitter T | 2010 | 11040084 | Hyposmia (Sinunasal) | 17 |  | 12 |
| Bitter T | 2011 | 11080271 | Parosmia | 22 | 22 | 1 |
| Biundo R | 2011 | 13100192 | Parkinson's Disease | 57 | 22 | 4 |
| Boccardi M | 2005 | 13100092 | Frontotemporal Dementia | 9 | 26 | 19 |
| Boddaert N | 2004 | 8050020 | Smith Magenis Syndrome | 5 | 12 | 4 |
| Boddaert N | 2004 | 8060159 | Autism Spectrum Disorder | 21 | 12 | 4 |
| Bodini B | 2009 | 11040159 | Multiple Sclerosis | 35 | 23 | 16 |
| Boghi A | 2011 | 13100004 | Anorexia Nervosa | 21 | 27 | 19 |
| Bonath B | 2018 | 19120002 | Attention Deficit Hyperactivity Disorder | 18 | 18 | 12 |
| Bonavita S | 2011 | 16030013 | Multiple Sclerosis | 36 | 18 | 24 |
| Bonekamp D | 2010 | 10030025 | Mild Cognitive Impairment | 10 | 20 | 1 |
| Bonilha L | 2004 | 8050021 | Epilepsy (Temporal Lobe Right-Sided) | 21 | 49 | 14 |
| Bonilha L | 2004 | 8050021 | Epilepsy (Temporal Lobe Left-Sided) | 22 |  | 20 |
| Borroni B | 2008 | 11040066 | Corticobasal Degeneration | 20 | 21 | 14 |
| Bossini L | 2017 | 21120112 | Post Traumatic Stress Disorder | 19 | 19 | 5 |
| Bouilleret V | 2008 | 11040160 | Epilepsy (Temporal Lobe) | 12 | 30 | 12 |
| Boxer A L | 2003 | 8050023 | Alzheimer's Disease | 11 | 15 | 3 |
| Boxer A L | 2003 | 8050023 | Semantic Dementia | 11 |  | 4 |
| Boxer A L | 2006 | 8060161 | Corticobasal Degeneration | 14 |  | 11 |
| Boxer A L | 2006 | 8060161 | Progressive Supranuclear Palsy | 15 |  | 8 |
| Boyen K | 2013 | 22110104 | Tinnitus | 31 | 24 | 7 |
| Bozzali M | 2006 | 10010013 | Alzheimer's Disease | 22 | 20 | 19 |
| Bozzali M | 2006 | 10010013 | Mild Cognitive Impairment (Converter) | 14 |  | 14 |
| Bozzali M | 2006 | 10010013 | Mild Cognitive Impairment (Non-converter) | 8 |  | 2 |
| Brambati S M | 2004 | 8050024 | Specific Learning Disorder | 10 | 11 | 9 |
| Brambati S M | 2009 | 14070012 | Semantic Dementia (Left Temporal Lobe Variant) | 13 | 25 | 15 |
| Brambati S M | 2009 | 14070012 | Semantic Dementia (Right Temporal Lobe Variant) | 6 |  | 15 |
| Brazdil M | 2009 | 10030016 | Epilepsy (Temporal Lobe) | 20 | 20 | 9 |
| Brenneis C | 2004 | 8050025 | Progressive Supranuclear Palsy | 12 | 12 | 12 |
| Brenneis C | 2003 | 8050027 | Multiple System Atrophy | 12 | 12 | 16 |
| Brenneis C | 2006 | 8050028 | Multiple-system Atrophy (Cerebellar Variant) | 13 | 14 | 16 |
| Brenneis C | 2006 | 8050028 | Spinocerebellar Ataxia | 22 |  | 15 |
| Brenneis C | 2003 | 8050029 | Spinocerebellar Ataxia | 9 | 23 | 15 |
| Brenneis C | 2004 | 8050030 | Lewy Body Dementia | 10 | 10 | 7 |
| Brenneis C | 2004 | 8050030 | Alzheimer's Disease | 10 |  | 14 |
| Brenneis C | 2005 | 8050031 | Narcolepsy | 12 | 12 | 3 |
| Brieber S | 2007 | 10010005 | [Attention Deficit Hyperactivity Disorder](https://www.nhs.uk/conditions/attention-deficit-hyperactivity-disorder-adhd/) | 15 | 15 | 9 |
| Brieber S | 2007 | 10010005 | Autism Spectrum Disorder | 15 | 15 | 6 |
| Brooks S J | 2011 | 13100007 | Anorexia Nervosa | 14 | 21 | 5 |
| Brooks S J | 2013 | 18100012 | Binge Eating Disorder | 59 | 97 | 1 |
| Brown W E | 2001 | 8050033 | Specific Learning Disorder | 16 | 14 | 8 |
| Brunner R | 2010 | 11040043 | Borderline Personality Disorder | 20 | 20 | 3 |
| Brys M | 2009 | 11040113 | Alzheimer's Disease | 8 | 21 | 9 |
| Budhiraja M | 2017 | 21090085 | Conduct Disorder | 31 | 25 | 4 |
| Burton E J | 2002 | 9010001 | Lewy Body Dementia | 25 | 25 | 29 |
| Burton E J | 2004 | 11040044 | Parkinson's Disease (With Dementia) | 26 | 36 | 37 |
| Burton E J | 2004 | 11040044 | Parkinson's Disease (Without Dementia) | 31 |  | 8 |
| Cai J | 2018 | 19120003 | Autism Spectrum Disorder | 38 | 27 | 3 |
| Camicioli R | 2009 | 13100096 | Parkinson's Disease | 43 | 43 | 3 |
| Canessa N | 2011 | 13100097 | Obstructive Sleep Apnea | 17 | 15 | 6 |
| Canu E | 2010 | 11080297 | Alzheimer's Disease (Late Onset) | 24 | 24 | 22 |
| Canu E | 2010 | 11080297 | Alzheimer's Disease (Early Onset) | 18 | 18 | 15 |
| Cao C | 2021 | 23090004 | Mild Cognitive Impairment | 20 | 29 | 4 |
| Carmona S | 2005 | 8060164 | Attention Deficit Hyperactivity Disorder | 25 | 25 | 17 |
| Caroli A | 2007 | 15010013 | Alzheimer's Disease | 9 | 17 | 5 |
| Caroli A | 2007 | 15010013 | Mild Cognitive Impairment | 14 |  | 2 |
| Castro-Fornieles J | 2009 | 9050023 | Anorexia Nervosa | 12 | 9 | 3 |
| Castro-Manglano P D | 2011 | 13100191 | Schizoaffective Disorder | 18 | 20 | 10 |
| Ceccarelli A | 2009 | 13100099 | Multiple Sclerosis | 18 | 17 | 3 |
| Ceccarelli A | 2008 | 16030058 | Multiple Sclerosis (Benign) | 19 | 20 | 2 |
| Ceccarelli A | 2008 | 16030058 | Multiple Sclerosis (Relapsing Remitting) | 15 |  | 2 |
| Ceko M | 2013 | 16030043 | Chronic Pain (Fibromyalgia) (Old) | 14 | 14 | 14 |
| Ceko M | 2013 | 16030043 | Chronic Pain (Fibromyalgia) (Young) | 14 | 15 | 9 |
| Celle S | 2010 | 11040114 | Restless Legs Syndrome | 17 | 54 | 3 |
| Cerasa A | 2013 | 16030014 | Multiple Sclerosis (Without Cerebellar Signs) | 14 | 20 | 2 |
| Cerasa A | 2013 | 16030014 | Multiple Sclerosis (With Cerebellar Signs) | 12 |  | 6 |
| Chan C H | 2006 | 8050038 | Epilepsy (Childhood Absence) | 13 | 109 | 4 |
| Chang C C | 2009 | 11040116 | Multiple System Atrophy (Cerebellar Variant) | 10 | 37 | 11 |
| Chang C C | 2009 | 11040116 | Multiple System Atrophy (Parkinsonian Variant) | 13 |  | 6 |
| Chang J L | 2005 | 8060166 | Amyotrophic Lateral Sclerosis | 20 | 22 | 14 |
| Chang S E | 2008 | 11040013 | Developmental Stuttering | 7 | 7 | 14 |
| Chanraud S | 2007 | 8050039 | Substance Use Disorder | 31 | 28 | 10 |
| Chanraud S | 2009 | 11040189 | Substance Use Disorder | 24 | 24 | 14 |
| Chao L L | 2012 | 14110028 | Post Traumatic Stress Disorder (Medicated) | 17 | 20 | 3 |
| Chao L L | 2012 | 14110028 | Post Traumatic Stress Disorder (Unmedicated) | 15 |  | 2 |
| Chen B | 2017 | 23090005 | Parkinson's Disease (With Mild Cognitive Impairment) | 21 | 21 | 10 |
| Chen B | 2017 | 23090005 | Parkinson's Disease (Without Mild Cognitive Impairment) | 11 |  | 10 |
| Chen F | 2016 | 23090008 | Parkinson's Disease | 37 | 21 | 9 |
| Chen M | 2016 | 23090007 | Parkinson's Disease | 23 | 15 | 10 |
| Chen S | 2006 | 8050042 | Post Traumatic Stress Disorder | 12 | 12 | 4 |
| Chen S | 2009 | 11040190 | Post Traumatic Stress Disorder | 12 | 12 | 3 |
| Chen W T | 2018 | 22010002 | Chronic Pain (Migraine) | 56 | 36 | 1 |
| Chen Y | 2012 | 14110029 | Post Traumatic Stress Disorder | 10 | 10 | 1 |
| Chen Y | 2018 | 22070093 | Frontotemporal Dementia | 45 | 35 | 1 |
| Chen Y | 2018 | 22070093 | Semantic Dementia | 28 |  | 2 |
| Chen Y | 2018 | 22070093 | Progressive Non Fluent Aphasia | 23 |  | 2 |
| Chen Y | 2019 | 23090006 | Parkinson's Disease | 61 | 59 | 27 |
| Cheng B | 2015 | 16010005 | Post Traumatic Stress Disorder | 30 | 30 | 3 |
| Cheng K | 2020 | 23090009 | Parkinson's Disease (With Fall) | 17 | 37 | 10 |
| Cheng K | 2020 | 23090009 | Parkinson's Disease (Without Fall) | 18 |  | 2 |
| Cheng Y | 2011 | 12060004 | Autism Spectrum Disorder (Asperger's Syndrome) | 11 | 25 | 13 |
| Cheng Y | 2011 | 12060004 | Autism Spectrum Disorder (Primary Autism) | 12 |  | 3 |
| Chenji S | 2020 | 21060013 | Amyotrophic Lateral Sclerosis | 22 | 43 | 1 |
| Chetelat G | 2002 | 8050043 | Mild Cognitive Impairment | 22 | 22 | 10 |
| Chetelat G | 2002 | 8050043 | Alzheimer's Disease | 16 |  | 15 |
| Clausi S | 2009 | 11040191 | Right Cerebellar Damage | 8 | 15 | 9 |
| Colloby S J | 2014 | 20100039 | Alzheimer's Disease | 47 | 39 | 4 |
| Colloby S J | 2014 | 20100039 | Lewy Body Dementia | 41 |  | 5 |
| Compta Y | 2012 | 13100193 | Parkinson's Disease (With Dementia) | 15 | 12 | 12 |
| Compta Y | 2012 | 13100193 | Parkinson's Disease (Without Dementia) | 18 |  | 5 |
| Corbo V | 2005 | 8050044 | Post Traumatic Stress Disorder | 14 | 14 | 5 |
| Cordato N J | 2005 | 8050045 | Progressive Supranuclear Palsy | 21 | 23 | 9 |
| Cordato N J | 2005 | 8050045 | Parkinson's Disease | 17 |  | 1 |
| Cormack F | 2005 | 8090163 | Mesial Temporal Sclerosis (Left) | 20 | 22 | 9 |
| Cormack F | 2005 | 8090163 | Mesial Temporal Sclerosis (Right) | 10 |  | 4 |
| Cosottini M | 2012 | 13100102 | Amyotrophic Lateral Sclerosis | 20 | 16 | 21 |
| Cousins K A Q | 2016 | 22060075 | Frontotemporal Dementia | 12 | 18 | 4 |
| Craig M C | 2007 | 11080274 | Autism Spectrum Disorder | 14 | 19 | 5 |
| Critchley H D | 2003 | 8050046 | Pure Autnomic Failure | 15 | 15 | 10 |
| D'Agata F | 2011 | 13100103 | Spinocerebellar Ataxia | 12 | 31 | 19 |
| D'Agata F | 2015 | 21080069 | Anorexia Nervosa | 21 | 17 | 7 |
| D'Agata F | 2015 | 21080069 | Bulimia Nervosa | 18 |  | 2 |
| Dalwani M | 2011 | 15090038 | Conduct Disorder | 25 | 19 | 4 |
| Dash S K | 2018 | 18100010 | Multiple System Atrophy (With Cerebellar Atrophy) | 30 | 25 | 4 |
| Dash S K | 2018 | 18100010 | Multiple System Atrophy (Without Cerebellar Atrophy) | 20 |  | 4 |
| Dasjamts T | 2011 | 20100040 | Alzheimer's Disease | 23 | 23 | 20 |
| Davis K D | 2007 | 16030044 | Chronic Pain | 9 | 11 | 2 |
| de Araujo-Filho G M | 2009 | 13100190 | Epilepsy | 54 | 30 | 6 |
| de Oliveira-Souza R | 2008 | 11040185 | Antisocial Personality Disorder | 15 | 15 | 22 |
| Della Nave R | 2008 | 9050027 | Friedreich's Ataxia | 22 | 25 | 2 |
| Della Nave R | 2008 | 11040117 | Spinocerebellar Ataxia (Type 1) | 10 | 10 | 7 |
| Della Nave R | 2008 | 11040117 | Spinocerebellar Ataxia (Type 2) | 10 |  | 5 |
| Delmaire C | 2007 | 11080275 | Dystonia | 30 | 30 | 6 |
| Dermody N | 2016 | 20070016 | Alzheimer's Disease | 25 | 22 | 4 |
| Dermody N | 2016 | 20070016 | Frontotemporal Dementia | 24 |  | 1 |
| Dermody N | 2016 | 22060076 | Alzheimer's Disease | 12 | 12 | 5 |
| Dermody N | 2016 | 22060076 | Frontotemporal Dementia | 12 |  | 5 |
| Desgranges B | 2007 | 12070014 | Semantic Dementia (Without Hypometabolism) | 10 | 17 | 6 |
| Desgranges B | 2007 | 12070014 | Semantic Dementia (With Hypometabolism) | 10 |  | 2 |
| Devenney E | 2015 | 22050054 | Frontotemporal Dementia | 58 | 25 | 7 |
| Di Paola M | 2007 | 8090166 | Alzheimer's Disease | 18 | 18 | 18 |
| Diez-Cirarda M | 2015 | 23090010 | Parkinson's Disease | 37 | 15 | 3 |
| Donnelly-Kehoe P A | 2019 | 22050055 | Frontotemporal Dementia (Country 1) | 44 | 60 | 13 |
| Donnelly-Kehoe P A | 2019 | 22050055 | Frontotemporal Dementia (Country 2) | 44 | 60 | 11 |
| Donnelly-Kehoe P A | 2019 | 22050055 | Frontotemporal Dementia (Country 3) | 44 | 60 | 6 |
| Draganski B | 2006 | 8050051 | Limb Amputation | 28 | 28 | 2 |
| Draganski B | 2003 | 8050052 | Dystonia (Idiopathic Cervical) | 10 | 10 | 3 |
| Droby A | 2021 | 23090011 | Parkinson's Disease | 16 | 20 | 2 |
| Eckart C | 2011 | 13100106 | Post Traumatic Stress Disorder | 20 | 13 | 5 |
| Ecker C | 2010 | 13100108 | Autism Spectrum Disorder | 22 | 22 | 14 |
| Ecker C | 2012 | 13100107 | Autism Spectrum Disorder | 89 | 89 | 11 |
| Eckert M A | 2005 | 8060170 | Specific Learning Disorder | 13 | 13 | 5 |
| Eckert M A | 2006 | 8060171 | Williams Syndrome | 8 | 9 | 20 |
| Ellis C M | 2001 | 13100109 | Amyotrophic Lateral Sclerosis | 16 | 8 | 3 |
| Eshaghi A | 2014 | 16030018 | Multiple Sclerosis | 36 | 19 | 6 |
| Etgen T | 2005 | 8060172 | Restless Legs Syndrome | 28 | 28 | 2 |
| Etgen T | 2006 | 10060056 | Dystonia (Primary Blepharospasm) | 16 | 16 | 1 |
| Fahim C | 2012 | 20060011 | Oppositional Defiant Disorder | 18 | 20 | 1 |
| Fairchild G | 2011 | 15090039 | Conduct Disorder (Early-Onset) | 36 | 27 | 6 |
| Fairchild G | 2011 | 15090039 | Conduct Disorder (Adolescent-Onset) | 27 |  | 4 |
| Fallon N | 2013 | 16030045 | Chronic Pain (Fibromyalgia) | 16 | 15 | 2 |
| Farb N A S | 2013 | 22040031 | Frontotemporal Dementia | 8 | 16 | 13 |
| Farrow T F D | 2007 | 13100110 | Alzheimer's Disease (Early-Onset) | 7 | 11 | 10 |
| Farrow T F D | 2007 | 13100110 | Alzheimer's Disease (non Early-Onset) | 7 | 11 | 10 |
| Feldmann A | 2008 | 11040163 | Posterior Cortical Atrophy | 1 | 20 | 7 |
| Feldmann A | 2008 | 11040163 | Alzheimer's Disease | 6 |  | 11 |
| Fernandez-Matarrubia M | 2017 | 22050036 | Frontotemporal Dementia | 26 | 24 | 9 |
| Fioravanti V | 2015 | 23090012 | Parkinson's Disease | 20 | 15 | 2 |
| Fiorenzato E | 2017 | 23090013 | Multiple System Atrophy | 72 | 36 | 31 |
| Focke N K | 2011 | 13060001 | Parkinson's Disease | 21 | 22 | 2 |
| Foster N E | 2015 | 19120005 | Autism Spectrum Disorder | 38 | 46 | 1 |
| Freitag C M | 2008 | 19120006 | Autism Spectrum Disorder | 15 | 15 | 1 |
| Friedrich H C | 2012 | 13100020 | Anorexia Nervosa (Without Weight-Restored) | 12 | 14 | 8 |
| Friedrich H C | 2012 | 13100020 | Anorexia Nervosa Patients (With Weight-Restored) | 13 |  | 2 |
| Frisoni G B | 2002 | 8090170 | Alzheimer's Disease | 29 | 26 | 34 |
| Galandra C | 2018 | 21090079 | Substance Use Disorder | 23 | 18 | 16 |
| Gale S D | 2005 | 8050053 | Traumatic Brain Injury | 9 | 9 | 16 |
| Gao Y | 2021 | 21090080 | Conduct Disorder | 34 | 34 | 3 |
| Gao Y | 2017 | 23090014 | Parkinson's Disease (With Mild Cognitive Impairment) | 23 | 21 | 6 |
| Gao Y | 2017 | 23090014 | Parkinson's Disease (Without Mild Cognitive Impairment) | 23 |  | 6 |
| Garrido L | 2009 | 11040091 | Developmental Prosopagnosia | 17 | 18 | 6 |
| Gaudio S | 2011 | 13100024 | Anorexia Nervosa | 15 | 16 | 3 |
| Gavazzi C | 2007 | 8050055 | Huntington's Disease | 9 | 11 | 4 |
| Gee J | 2003 | 8050056 | Alzheimer's Disease | 12 | 12 | 7 |
| Gee J | 2003 | 8050056 | Frontotemporal Dementia | 29 |  | 11 |
| Geha P Y | 2008 | 13100112 | Chronic Pain (Old) | 26 | 11 | 1 |
| Geha P Y | 2008 | 13100112 | Chronic Pain (Young) | 11 | 11 | 1 |
| Gerrits N J H M | 2014 | 23090015 | Parkinson's Disease | 93 | 46 | 8 |
| Ghosh B C | 2012 | 14050007 | Progressive Supranuclear Palsy | 23 | 22 | 2 |
| Gilbert A R | 2008 | 9050032 | Obsessive Compulsive Disorder | 25 | 20 | 7 |
| Gili T | 2011 | 20110041 | Alzheimer's Disease | 11 | 10 | 11 |
| Ginestroni A | 2008 | 9050034 | Spinocerebellar Ataxia | 15 | 15 | 4 |
| Giordano A | 2013 | 14050008 | Progressive Supranuclear Palsy | 15 | 15 | 6 |
| Gobbi C | 2014 | 16030020 | Multiple Sclerosis (Non-Depressed) | 54 | 90 | 56 |
| Gobbi C | 2014 | 16030020 | Multiple Sclerosis (Depressed) | 69 |  | 56 |
| Gobbi C | 2014 | 16030020 | Multiple Sclerosis (Non-Fatigued) | 59 |  | 56 |
| Gobbi C | 2014 | 16030020 | Multiple Sclerosis (Fatigued) | 64 |  | 56 |
| Gold B T | 2010 | 11040071 | Mild Cognitive Impairment | 12 | 14 | 4 |
| Granert O | 2011 | 13100117 | Dystonia | 11 | 12 | 4 |
| Gregory S | 2012 | 15090040 | Psychopathy | 17 | 22 | 18 |
| Greimel E | 2013 | 19120006 | Autism Spectrum Disorder | 47 | 51 | 4 |
| Grieder M | 2013 | 20110042 | Alzheimer's Disease | 14 | 19 | 1 |
| Gross R G | 2010 | 10060039 | Corticobasal Degeneration | 20 | 8 | 6 |
| Grosskreutz J | 2006 | 8050063 | Amyotrophic Lateral Sclerosis | 17 | 17 | 16 |
| Grossman M | 2004 | 9050035 | Corticobasal Degeneration | 9 | 12 | 9 |
| Grossman M | 2004 | 9050035 | Semantic Dementia | 8 |  | 4 |
| Grossman M | 2004 | 9050035 | Progressive Non-Fluent Aphasia | 7 |  | 6 |
| Grossman M | 2004 | 9050035 | Frontotemporal Dementia (Non-Aphasic) | 14 |  | 5 |
| Guedj E | 2009 | 14080019 | Mild Cognitive Impairment | 19 | 28 | 7 |
| Guggenmos M | 2017 | 21020006 | Substance Use Disorder | 119 | 97 | 22 |
| Guimaraes R P | 2017 | 23090016 | Parkinson's Disease (Moderate) | 66 | 40 | 2 |
| Guimaraes R P | 2017 | 23090016 | Parkinson's Disease (Severe) | 66 |  | 6 |
| Guo X | 2010 | 11040119 | Alzheimer's Disease | 13 | 14 | 16 |
| Gustin S M | 2011 | 11080299 | Chronic Pain (Trigeminal Neuropathic Pain) | 21 | 30 | 7 |
| Gwilym S E | 2010 | 11020004 | Chronic Pain | 16 | 16 | 2 |
| Hafkemeijer A | 2017 | 22070096 | Frontotemporal Dementia | 12 | 22 | 1 |
| Hakamata Y | 2007 | 11040195 | Post Traumatic Stress Disorder | 14 | 70 | 1 |
| Hall A M | 2008 | 11040017 | Alzheimer's Disease (Converted) | 21 | 127 | 2 |
| Hall A M | 2008 | 11040017 | Alzheimer's Disease (Non-Converted) | 26 |  | 2 |
| Halpern C H | 2004 | 8060190 | Corticobasal Degeneration | 5 | 12 | 4 |
| Halpern C H | 2004 | 8060190 | Semantic Dementia | 3 |  | 1 |
| Hamalainen A | 2007 | 9010002 | Alzheimer's Disease | 15 | 21 | 19 |
| Hamalainen A | 2007 | 9010002 | Mild Cognitive Impairment | 14 |  | 6 |
| Hamalainen A | 2007 | 14080024 | Mild Cognitive Impairment (Progressive) | 13 | 22 | 29 |
| Hamalainen A | 2007 | 14080024 | Mild Cognitive Impairment (Stable) | 43 |  | 35 |
| Han X | 2017 | 17070010 | Multiple Sclerosis | 20 | 20 | 11 |
| He N | 2015 | 19120007 | Attention Deficit Hyperactivity Disorder | 37 | 35 | 4 |
| Healey M L | 2015 | 22060084 | Frontotemporal Dementia | 12 | 14 | 5 |
| Henley S M | 2009 | 11040122 | Huntington's Disease | 40 | 20 | 27 |
| Henry R G | 2008 | 9080093 | Clinically Isolated Syndrome | 41 | 49 | 15 |
| Herringa R | 2012 | 14110033 | Post Traumatic Stress Disorder | 13 | 28 | 4 |
| Hirao K | 2006 | 8050067 | Alzheimer's Disease | 61 | 61 | 2 |
| Hoeft F | 2007 | 13100121 | Specific Learning Disorder | 19 | 19 | 6 |
| Holzapfel M | 2006 | 8060194 | Turner Syndrome | 10 | 10 | 4 |
| Honea R A | 2009 | 11040123 | Alzheimer's Disease | 60 | 56 | 13 |
| Hong J Y | 2012 | 23090017 | Parkinson's Disease (Without Subjective Memory Complaints) | 15 | 25 | 6 |
| Hong J Y | 2012 | 23090017 | Parkinson's Disease (With Subjective Memory Complaints) | 20 |  | 5 |
| Huang C W | 2018 | 20110045 | Alzheimer's Disease | 50 | 30 | 5 |
| Huang L | 2019 | 23090019 | Parkinson's Disease | 9 | 7 | 9 |
| Huang W | 2011 | 15070020 | Epilepsy (Generalized Seizure Disorder) | 31 | 37 | 12 |
| Huebner T | 2008 | 11040094 | Conduct Disorder | 23 | 23 | 3 |
| Huey E D | 2009 | 11040125 | Corticobasal Degeneration | 48 | 14 | 19 |
| Hüfner K | 2009 | 11040020 | Unilateral Vestibular Deafferentation Syndrome | 16 | 16 | 12 |
| Hüfner K | 2007 | 11040021 | Downbeat Nystagmus | 11 | 15 | 7 |
| Huynth N T | 2014 | 21110101 | Obstructive Sleep Apnea | 13 | 7 | 1 |
| Hyde K L | 2010 | 11040196 | Autism Spectrum Disorder | 15 | 13 | 3 |
| Iannaccone R | 2015 | 19120008 | Attention Deficit Hyperactivity Disorder | 18 | 18 | 3 |
| Ille R | 2011 | 13100030 | Huntington's Disease | 18 | 18 | 10 |
| Irish M | 2013 | 2260079 | Alzheimer's Disease | 10 | 10 | 5 |
| Irish M | 2013 | 2260079 | Frontotemporal Dementia | 10 |  | 10 |
| Ishii K | 2005 | 10060059 | Alzheimer's Disease | 30 | 30 | 3 |
| Ivo R | 2013 | 16030046 | Chronic Pain (Chronic Lower Back Pain) | 14 | 14 | 16 |
| Jagger-Rickels A C | 2018 | 19120001 | Attention Deficit Hyperactivity Disorder | 41 | 32 | 15 |
| Jagger-Rickels A C | 2018 | 19120001 | Specific Learning Disorder | 17 |  | 12 |
| Jang D P | 2007 | 11080278 | Substance Use Disorder | 20 | 20 | 12 |
| Jauhiainen A M | 2008 | 14080025 | Mild Cognitive Impairment | 7 | 13 | 4 |
| Johnston B A | 2014 | 19120009 | Attention Deficit Hyperactivity Disorder | 34 | 34 | 12 |
| Joo E Y | 2010 | 11040127 | Obstructive Sleep Apnea | 36 | 31 | 27 |
| Joos A | 2010 | 10060042 | Anorexia Nervosa | 12 | 18 | 7 |
| Joubert S | 2006 | 8050072 | Frontotemporal Dementia | 3 | 20 | 7 |
| Jurkiewicz M T | 2006 | 8050073 | Traumatic Brain Injury | 17 | 17 | 2 |
| Kanda T | 2008 | 9050040 | Alzheimer's Disease | 20 | 20 | 7 |
| Kanda T | 2008 | 9050040 | Frontotemporal Dementia | 13 |  | 9 |
| Kappel V | 2014 | 19120010 | Attention Deficit Hyperactivity Disorder (Adults) | 16 | 20 | 5 |
| Kappel V | 2014 | 19120010 | Attention Deficit Hyperactivity Disorder (Children) | 14 |  | 3 |
| Karlsson H | 2014 | 18090003 | Binge Eating Disorder | 23 | 22 | 7 |
| Kasai K | 2008 | 12070015 | Post Traumatic Stress Disorder | 18 | 23 | 7 |
| Kassubek J | 2005 | 11040221 | Huntington's Disease | 44 | 22 | 2 |
| Kassubek J | 2007 | 11080280 | Kennedy Disease | 18 | 20 | 10 |
| Kassubek J | 2002 | 8090174 | Epilepsy | 7 | 30 | 8 |
| Kassubek J | 2004 | 8050075 | Huntington's Disease | 44 | 22 | 17 |
| Kassubek J | 2007 | 12070016 | Spastic Paraparesis (Complicated Hereditary) | 12 | 15 | 7 |
| Kassubek J | 2007 | 12070016 | Spastic Paraparesis (Pure Hereditary) | 21 | 17 | 5 |
| Kato S | 2012 | 13110214 | Parkinson's Disease | 9 | 22 | 11 |
| Kaufman | 2013 | 21070046 | Autism Spectrum Disorder | 10 | 10 | 1 |
| Kaufmann C | 2002 | 8050076 | Narcolepsy | 12 | 32 | 19 |
| Kawachi T | 2006 | 8050077 | Alzheimer's Disease | 32 | 30 | 14 |
| Kawasaki Y | 2004 | 11080309 | Schizotypal Disorder | 25 | 50 | 4 |
| Ke X | 2008 | 11040048 | Autism Spectrum Disorder | 17 | 15 | 1 |
| Keller S S | 2002 | 8060206 | Epilepsy (Temporal Lobe Left) | 58 | 58 | 14 |
| Keller S S | 2002 | 8060206 | Epilepsy (Temporal Lobe Right) | 58 |  | 13 |
| Keller S S | 2007 | 8090175 | Epilepsy (Rendered Free of Seizures Post Surgery) | 10 | 77 | 4 |
| Keller S S | 2007 | 8090175 | Epilepsy (Persistent Post Surgical Seizures) | 12 |  | 3 |
| Khaleeli Z | 2007 | 16030022 | Multiple Sclerosis | 46 | 23 | 7 |
| Kim E J | 2007 | 13100035 | Frontotemporal Lobar Degeneration (Tau Positive) | 6 | 61 | 7 |
| Kim E J | 2007 | 13100035 | Frontotemporal Lobar Degeneration (Tau Negative) | 8 |  | 8 |
| Kim J H | 2008 | 9050043 | Chronic Pain (Migraine) | 20 | 33 | 20 |
| Kim J H | 2007 | 11080281 | Epilepsy (Juvenile Myoclonic) | 25 | 44 | 2 |
| Kim S | 2011 | 13100127 | Alzheimer's Disease | 61 | 33 | 10 |
| Kim S J | 2009 | 11040050 | Narcolepsy | 17 | 17 | 29 |
| Kirchner H | 2011 | 13100129 | Ataxia | 31 | 19 | 21 |
| Kobayakawa M | 2017 | 23090020 | Parkinson's Disease | 20 | 37 | 6 |
| Kobel M | 2010 | 13100130 | Attention Deficit Hyperactivity Disorder | 14 | 12 | 1 |
| Koenig P | 2008 | 11050243 | Alzheimer's Disease | 6 | 11 | 8 |
| Koenigkam-Santos M | 2008 | 9050084 | Kallman Syndrome | 21 | 16 | 2 |
| Koprivova J | 2009 | 11040051 | Obsessive Compulsive Disorder | 14 | 15 | 30 |
| Koskenkorva P | 2009 | 11040052 | Unverricht-Lundborg Disease | 34 | 30 | 10 |
| Kostic V S | 2010 | 13100197 | Parkinson's Disease (Depressed) | 16 | 26 | 15 |
| Kostic V S | 2010 | 13100197 | Parkinson's Disease (Non-Depressed) | 24 |  | 15 |
| Kronbichler M | 2008 | 11080295 | Specific Learning Disorder | 13 | 15 | 9 |
| Kuchinad A | 2007 | 13110205 | Chronic Pain (Fibromyalgia) | 10 | 10 | 5 |
| Kumar U | 2017 | 19120011 | Attention Deficit Hyperactivity Disorder | 18 | 18 | 4 |
| Kumfor F | 2014 | 22050061 | Alzheimer's Disease | 14 | 12 | 1 |
| Kumfor F | 2014 | 22050061 | Frontotemporal Dementia | 13 |  | 1 |
| Kumfor F | 2019 | 22050058 | Frontotemporal Dementia | 25 | 24 | 4 |
| Kumfor F | 2019 | 22050058 | Semantic Dementia | 14 |  | 4 |
| Kunst J | 2019 | 23090021 | Alzheimer's Disease | 12 | 58 | 3 |
| Kunst J | 2019 | 23090021 | Mild Cognitive Impairment | 27 |  | 5 |
| Kunst J | 2019 | 23090021 | Parkinson's Disease | 23 |  | 3 |
| Kurth F | 2011 | 12060005 | Autism Spectrum Disorder | 52 | 52 | 1 |
| Kwon H | 2004 | 8050081 | Autism Spectrum Disorder (Asperger's Syndrome) | 11 | 13 | 3 |
| Labate A | 2010 | 11040074 | Epilepsy (Temporal Lobe Mild) | 19 | 37 | 3 |
| Labate A | 2010 | 11040074 | Epilepsy (Temporal Lobe Refractory) | 19 |  | 1 |
| Lagarde J | 2013 | 14050009 | Progressive Supranuclear Palsy | 19 | 18 | 5 |
| Lagarde J | 2013 | 14050009 | Frontotemporal Dementia | 16 |  | 8 |
| Lagarde J | 2015 | 20110046 | Alzheimer's Disease | 14 | 18 | 3 |
| Lagarde J | 2015 | 20110046 | Frontotemporal Dementia | 18 |  | 10 |
| Lagarde J | 2015 | 20110046 | Progressive Supranuclear Palsy | 21 |  | 10 |
| Lai C H | 2012 | 16080066 | Panic Disorder | 30 | 21 | 4 |
| Lai C H | 2015 | 17050006 | Panic Disorder | 53 | 54 | 2 |
| Lee E | 2013 | 23090025 | Parkinson's Disease | 40 | 40 | 11 |
| Lee J E | 2013 | 13110212 | Parkinson's Disease (Mild Cognitive Impairment Converters) | 15 | 21 | 8 |
| Lee J E | 2013 | 13110212 | Parkinson's Disease (Mild Cognitive Impairment Non-Converters) | 36 |  | 3 |
| Lee J E | 2010 | 23090024 | Parkinson's Disease | 41 | 21 | 3 |
| Lee J E | 2014 | 23100026 | Parkinson's Disease | 38 | 50 | 7 |
| Lee W | 2017 | 23090023 | Parkinson's Disease (With Visual Hallucinations) | 10 | 30 | 2 |
| Lee W | 2017 | 23090023 | Parkinson's Disease (Without Visual Hallucinations) | 21 |  | 2 |
| Lehmann M | 2011 | 20110047 | Alzheimer's Disease | 30 | 50 | 1 |
| Lehmann M | 2011 | 20110047 | Posterior Cortical Atrophy | 48 |  | 1 |
| Lentini E | 2020 | 20070025 | Klinefelter's Syndrome (XXY) (Male) | 33 | 41 | 11 |
| Lentini E | 2020 | 20070025 | Klinefelter's Syndrome (XXY) (Female) | 33 | 45 | 7 |
| Leutgeb V | 2016 | 22030018 | Borderline Personality Disorder | 20 | 19 | 3 |
| Li L | 2006 | 8050084 | Post Traumatic Stress Disorder | 12 | 12 | 4 |
| Li L | 2022 | 23100030 | Parkinson's Disease (With Mild Cognitive Impairment) | 23 | 23 | 2 |
| Li L | 2022 | 23100031 | Parkinson's Disease (Without Mild Cognitive Impairment) | 22 |  | 2 |
| Li X | 2015 | 19120024 | Attention Deficit Hyperactivity Disorder | 30 | 30 | 2 |
| Li Y | 2023 | 23100029 | Mild Cognitive Impairment | 44 | 40 | 3 |
| Li Y | 2016 | 23100028 | Parkinson's Disease | 23 | 20 | 3 |
| Li Y | 2020 | 23100031 | Parkinson's Disease | 98 | 66 | 2 |
| Li Z | 2020 | 22010005 | Chronic Pain (Migraine) | 72 | 46 | 2 |
| Liang P | 2016 | 23100032 | Parkinson's Disease | 16 | 21 | 3 |
| Liao M | 2013 | 20070026 | Anxiety Disorder | 26 | 25 | 1 |
| Libon D J | 2009 | 13100040 | Progressive Non-Fluent Aphasia | 11 | 43 | 13 |
| Libon D J | 2009 | 13100040 | Semantic Dementia | 10 |  | 7 |
| Libon D J | 2009 | 13100040 | Frontotemporal Lobar Degeneration | 51 |  | 13 |
| Lim L | 2013 | 19120012 | Attention Deficit Hyperactivity Disorder | 29 | 29 | 3 |
| Lin A | 2013 | 16030024 | Multiple Sclerosis | 11 | 11 | 5 |
| Lin C H | 2013 | 13100196 | Essential Tremor | 10 | 13 | 19 |
| Lin C H | 2013 | 13100196 | Parkinson's Disease | 10 |  | 15 |
| Lin K | 2009 | 11040169 | Epilepsy (Juvenile Myoclonic Photosensitivity) | 19 | 30 | 4 |
| Lin K | 2009 | 11040169 | Epilepsy (Juvenile Myoclonic Without Photosensitivity) | 41 |  | 8 |
| Liu H | 2020 | 22010006 | Chronic Pain (Migraine) | 37 | 37 | 1 |
| Liu M | 2011 | 15070021 | Epilepsy (Generalized Seizure Disorder) | 10 | 10 | 6 |
| Liu M | 2011 | 15070021 | Epilepsy (Juvenile Myoclonic) | 15 | 15 | 5 |
| Liu Y | 2018 | 22110106 | Tinnitus | 24 | 24 | 5 |
| Long Z | 2016 | 20110048 | Mild Cognitive Impairment | 29 | 33 | 3 |
| Lu C | 2010 | 11040053 | Developmental Stuttering | 12 | 12 | 9 |
| Ludolph A G | 2006 | 8060209 | Tourette Syndrome | 14 | 15 | 2 |
| Ma Z | 2019 | 21110103 | Generalized Anxiety Disorder | 15 | 28 | 2 |
| Maeda Y | 2013 | 16030047 | Chronic Pain (Carpal Tunnel Syndrome) | 28 | 28 | 1 |
| Maier S | 2016 | 19120015 | Attention Deficit Hyperactivity Disorder | 131 | 95 | 1 |
| Mallik S | 2015 | 16060063 | Multiple Sclerosis (Relapsing-Remitting) | 51 | 29 | 7 |
| Mallik S | 2015 | 16060063 | Multiple Sclerosis (Secondary Progressive) | 28 |  | 4 |
| Maneru C | 2003 | 10060046 | Hypoxic-Ischemic Encephalopathy | 13 | 13 | 9 |
| Mannerkoski M K | 2009 | 11040199 | Intellectual Disability | 26 | 39 | 2 |
| Martikainen I K | 2013 | 16030048 | Chronic Pain (Chronic Nonspecific Back Pain) | 16 | 16 | 3 |
| Martinez-Horta S | 2020 | 21060027 | Huntington's Disease (Non-Demented) | 20 | 15 | 8 |
| Martinez-Horta S | 2020 | 21060027 | Huntington's Disease (Demented) | 15 | 0 | 8 |
| Martino D | 2011 | 13100138 | Dystonia (Primary Blespharospasm) | 25 | 24 | 6 |
| Massana G | 2003 | 8050087 | Panic Disorder | 18 | 18 | 1 |
| Massimo L | 2009 | 11040200 | Persistent Depressive Disorder | 9 | 27 | 22 |
| Massimo L | 2009 | 11040200 | Histrionic Personality Disorder | 5 |  | 24 |
| Massimo L | 2013 | 22060086 | Alzheimer's Disease | 17 | 30 | 13 |
| Massimo L | 2013 | 22060086 | Frontotemporal Dementia | 37 |  | 14 |
| Matsuda H | 2002 | 8090180 | Alzheimer's Disease | 15 | 25 | 13 |
| Matsumoto R | 2010 | 13100139 | Obsessive Compulsive Disorder | 16 | 32 | 4 |
| Matsunari I | 2007 | 9050048 | Alzheimer's Disease | 27 | 40 | 9 |
| Mazere J | 2008 | 13100140 | Alzheimer's Disease | 8 | 8 | 6 |
| McAlonan G M | 2007 | 13100043 | Attention Deficit Hyperactivity Disorder | 28 | 31 | 8 |
| McAlonan G M | 2005 | 12060007 | Autism Spectrum Disorder | 17 | 17 | 13 |
| McAlonan G M | 2008 | 11040026 | Autism Spectrum Disorder (High Functioning Autism) | 17 | 55 | 8 |
| McAlonan G M | 2008 | 11040026 | Autism Spectrum Disorder (Asperger's Syndrome) | 16 |  | 4 |
| McAlonan G M | 2002 | 12060006 | Autism Spectrum Disorder | 17 | 24 | 9 |
| McMillan A B | 2004 | 8050091 | Epilepsy (Left Temporal Lobe) | 13 | 62 | 7 |
| McMillan A B | 2004 | 8050091 | Lobe Epilepsy (Right Temporal Lobe) | 12 |  | 15 |
| Melloni M | 2016 | 22040033 | Alzheimer's Disease | 21 | 22 | 23 |
| Melloni M | 2016 | 22040033 | Frontotemporal Dementia | 26 |  | 6 |
| Melloni M | 2015 | 23100035 | Parkinson's Disease | 14 | 13 | 3 |
| Mengotti P | 2011 | 12060001 | Autism Spectrum Disorder (Children) | 7 | 7 | 2 |
| Mengotti P | 2011 | 12060001 | Autism Spectrum Disorder | 20 | 22 | 2 |
| Meppelink A M | 2011 | 13100199 | Parkinson's Disease (Without Visual Hallucinations) | 13 | 14 | 13 |
| Meppelink A M | 2011 | 13100199 | Parkinson's diease (With Visual Hallucinations) | 11 |  | 17 |
| Mesaros S | 2008 | 13100143 | Multiple Sclerosis (Secondary Progressive) | 35 | 21 | 64 |
| Mesaros S | 2008 | 13100143 | Multiple Sclerosis (Benign) | 60 |  | 31 |
| Mesaros S | 2008 | 9050051 | Multiple Sclerosis | 28 | 21 | 2 |
| Meyer S | 2017 | 22050046 | Frontotemporal Dementia | 52 | 52 | 2 |
| Mezzapesa D M | 2007 | 9050052 | Amyotrophic Lateral Sclerosis | 16 | 9 | 13 |
| Miettinen P S | 2011 | 13100144 | Alzheimer's Disease | 16 | 21 | 5 |
| Miettinen P S | 2011 | 13100144 | Mild Cognitive Impairment | 18 |  | 5 |
| Migliaccio R | 2009 | 11050261 | Alzheimer's Disease | 16 | 65 | 18 |
| Migliaccio R | 2009 | 11050261 | Progressive Non-Fluent Aphasia | 10 |  | 18 |
| Migliaccio R | 2009 | 11050261 | Posterior Cortical Atrophy | 14 |  | 18 |
| Milham M P | 2005 | 8050094 | Anxiety Disorder | 17 | 34 | 6 |
| Minnerop M | 2007 | 10010002 | Multiple System Atrophy (Cerebellar) | 16 | 16 | 18 |
| Minnerop M | 2007 | 10010002 | Multiple System Atrophy (Parkinsonian) | 16 |  | 22 |
| Minnerop M | 2008 | 11040057 | Dystonia | 13 | 13 | 5 |
| Molko N | 2003 | 8090182 | Turner Syndrome | 14 | 14 | 2 |
| Molko N | 2004 | 8060217 | Turner Syndrome | 14 | 14 | 12 |
| Moon C | 2014 | 21110106 | Generalized Anxiety Disorder | 13 | 9 | 5 |
| Moon C | 2015 | 21110105 | Generalized Anxiety Disorder | 17 | 17 | 5 |
| Moon C | 2018 | 21060016 | Obsessive Compulsive Disorder | 18 | 18 | 3 |
| Moorhead T W | 2005 | 8090183 | Specific Learning Disorder | 18 | 29 | 9 |
| Morbelli S | 2016 | 22050047 | Frontotemporal Dementia (Positive Symptoms) | 12 | 20 | 5 |
| Morbelli S | 2016 | 22050047 | Frontotemporal Dementia (Negative Symptoms) | 13 |  | 4 |
| Mordasini L | 2012 | 16030049 | Chronic Pain | 20 | 20 | 3 |
| Morgan B | 2011 | 22060088 | Frontotemporal Dementia | 14 | 12 | 2 |
| Morgen K | 2006 | 8060218 | Multiple Sclerosis | 19 | 19 | 2 |
| Morrell M J | 2010 | 13100146 | Obstructive Sleep Apnea | 60 | 60 | 2 |
| Mueller S G | 2006 | 8050095 | Epilepsy (With Mesial Temporal Sclerosis) | 26 | 30 | 28 |
| Muhlau M | 2013 | 16030026 | Clinically Isolated Syndrome | 249 | 49 | 12 |
| Muhlau M | 2007 | 8050096 | Huntington's Disease | 46 | 46 | 32 |
| Muhlau M | 2006 | 8060219 | Tinnitus | 28 | 28 | 1 |
| Muller S | 2013 | 19120018 | Autism Spectrum Disorder | 12 | 12 | 10 |
| Muller-Vahl K R | 2009 | 11040201 | Tourette Syndrome | 19 | 20 | 14 |
| Mummery C J | 2000 | 8060220 | Semantic Dementia | 6 | 14 | 17 |
| Na K S | 2013 | 17050007 | Panic Disorder (Without Agoraphobia) | 22 | 22 | 2 |
| Na K S | 2013 | 17050007 | Panic Disorder (With Agoraphobia) | 12 |  | 7 |
| Naduthota R | 2017 | 23100037 | Parkinson's Disease | 72 | 72 | 18 |
| Nagano-Saito A | 2005 | 8050097 | Parkinson's Disease | 19 | 31 | 3 |
| Nardo D | 2010 | 11050255 | Post Traumatic Stress Disorder | 21 | 22 | 5 |
| Nestor P J | 2003 | 8090184 | Progressive Non Fluent Aphasia | 10 | 10 | 1 |
| Nevler N | 2017 | 22060089 | Frontotemporal Dementia | 32 | 17 | 8 |
| Niedtfeld I | 2013 | 14110036 | Borderline Personality Disorder | 60 | 60 | 2 |
| Nishio Y | 2010 | 10060054 | Parkinson's Disease | 40 | 13 | 36 |
| Nyatega C O | 2022 | 23100038 | Parkinson's Disease | 20 | 18 | 4 |
| O'Callaghan C | 2019 | 22070094 | Alzheimer's Disease | 24 | 37 | 2 |
| O'Callaghan C | 2019 | 22070094 | Frontotemporal Dementia | 31 |  | 3 |
| O'Doherty D C M | 2017 | 21120115 | Post Traumatic Stress Disorder | 25 | 25 | 26 |
| O'Muircheartaigh J | 2011 | 13100150 | Epilepsy (Juvenile Myoclonic) | 28 | 55 | 2 |
| Obermann M | 2007 | 8060221 | Blepharopasm | 11 | 11 | 4 |
| Obermann M | 2007 | 8060221 | Dystonia | 9 | 9 | 3 |
| Obermann M | 2013 | 16030050 | Chronic Pain (Trigeminal Neuralgia) | 60 | 49 | 14 |
| Ohnishi T | 2001 | 8050099 | Alzheimer's Disease | 26 | 23 | 2 |
| Okada T | 2004 | 8050100 | Chronic Pain | 16 | 49 | 2 |
| Overmeyer S | 2001 | 13100054 | Attention Deficit Hyperactivity Disorder | 18 | 16 | 9 |
| Padovani A | 2006 | 8050102 | Progress Supranuclear Palsy | 14 | 14 | 22 |
| Pagonabarraga J | 2014 | 23100039 | Parkinson's Disease | 15 | 15 | 1 |
| Pail M | 2010 | 11080301 | Epilepsy (Left Mesial Temporal Lobe) | 20 | 40 | 1 |
| Pail M | 2010 | 11080301 | Epilepsy (Right Mesial Temporal Lobe) | 20 |  | 1 |
| Pan W J | 2007 | 11040203 | Blindness | 14 | 16 | 3 |
| Pannacciulli N | 2006 | 8050103 | Binge Eating Disorder | 24 | 36 | 7 |
| Pantano P | 2011 | 13100055 | Dystonia | 19 | 28 | 8 |
| Pardini M | 2009 | 11040175 | Corticobasal Degeneration | 25 | 12 | 4 |
| Pardini M | 2009 | 11040175 | Frontotemporal Dementia | 22 |  | 5 |
| Parisi L | 2014 | 16030028 | Multiple Sclerosis (Classic) | 9 | 9 | 3 |
| Parisi L | 2014 | 16030028 | Multiple Sclerosis (Cortical) | 9 |  | 5 |
| Park K M | 2015 | 22060070 | Transient Global Amnesia | 80 | 29 | 4 |
| Peinemann A | 2005 | 9010007 | Huntington's Disease | 25 | 25 | 7 |
| Pell G S | 2008 | 9050055 | Epilepsy | 19 | 115 | 18 |
| Pennanen C | 2005 | 8050105 | Mild Cognitive Impairment | 51 | 32 | 10 |
| Pereira J B | 2009 | 11040060 | Parkinson's Disease | 36 | 20 | 30 |
| Pereira J M | 2009 | 11050263 | Alzheimer's Disease | 3 | 25 | 1 |
| Pereira J M | 2009 | 11050263 | Frontotemporal Dementia (With Tau Inclusions) | 6 |  | 4 |
| Pereira J M | 2009 | 11050263 | Frontotemporal Dementia (With Ubiquitin Inclusions) | 9 |  | 3 |
| Pereira J M | 2009 | 11050263 | Progressive Non Fluent Aphasia | 3 |  | 2 |
| Pereira J M | 2009 | 11050263 | Semantic Dementia (With Tau Inclusions) | 3 |  | 4 |
| Pereira J M | 2009 | 11050263 | Semantic Dementia (With Ubiquitin Inclusions) | 5 |  | 5 |
| Pereira J M | 2009 | 11050263 | Frontotemporal Dementia | 4 |  | 2 |
| Planetta P J | 2015 | 23100040 | Multiple System Atrophy | 14 | 14 | 5 |
| Planetta P J | 2015 | 23100040 | Parkinson's Disease | 14 |  | 6 |
| Potgeiser A R E | 2014 | 23100041 | Parkinson's Disease | 77 | 87 | 6 |
| Prakash R S | 2010 | 16030029 | Multiple Sclerosis | 21 | 15 | 12 |
| Preziosa P | 2016 | 17070011 | Multiple Slerosis (Cognitively Impaired) | 23 | 61 | 10 |
| Preziosa P | 2016 | 17070011 | Multiple Sclerosis (Cognitively Preserved) | 38 |  | 4 |
| Prinster A | 2006 | 8050107 | Multiple Sclerosis | 51 | 34 | 8 |
| Prinster A | 2010 | 11040140 | Multiple Sclerosis | 128 | 35 | 20 |
| Ptito M | 2008 | 9050058 | Blindness | 11 | 21 | 10 |
| Pujol J | 2004 | 13100059 | Obsessive Compulsive Disorder | 72 | 72 | 3 |
| Quarantelli M | 2006 | 8050108 | Dystrophy (Facioscapulohumeral) | 30 | 39 | 9 |
| Quattrone A | 2008 | 9080100 | Essential Tremor (Arm) | 43 | 32 | 1 |
| Quattrone A | 2008 | 9080100 | Essential Tremor (Head) | 50 |  | 3 |
| Rabinovici G D | 2007 | 11040099 | Alzheimer's Disease | 11 | 40 | 19 |
| Rabinovici G D | 2007 | 11040099 | Frontotemporal Lobar Degeneration | 18 |  | 38 |
| Raji C A | 2009 | 11040030 | Alzheimer's Disease | 33 | 169 | 41 |
| Rami L | 2009 | 11040232 | Alzheimer's Disease | 31 | 27 | 6 |
| Rami L | 2009 | 11040232 | Mild Cognitive Impairment | 14 |  | 5 |
| Ramirez-Ruiz B | 2007 | 13100195 | Parkinson's Disease (Without Visual Hallucinations) | 20 | 21 | 2 |
| Ramirez-Ruiz B | 2007 | 13100195 | Parkinson's Disease (With Visual Hallucinations) | 18 |  | 10 |
| Ramos-Quiroga J A | 2016 | 19120017 | Attention Deficit Hyperactivity Disorder | 44 | 44 | 3 |
| Ranjeva J P | 2005 | 16030031 | Clinically Isolated Syndrome | 18 | 18 | 8 |
| Rankin K P | 2011 | 21050002 | Frontotemporal Dementia (Behavioral Variant) | 3 | 53 | 23 |
| Rankin K P | 2011 | 21050002 | Frontotemporal Dementia (Pick Disease) | 5 |  | 22 |
| Reetz K | 2011 | 13100157 | Spinocerebellar Ataxia | 16 | 16 | 12 |
| Reiss A L | 2004 | 8060222 | Williams Syndrome | 43 | 40 | 13 |
| Remy F | 2005 | 8090188 | Alzheimer's Disease | 8 | 11 | 21 |
| Riccitelli G | 2011 | 16030059 | Multiple Sclerosis (Fatigued) | 10 | 14 | 7 |
| Riccitelli G | 2011 | 16030059 | Multiple Sclerosis (Non-Fatigued) | 14 |  | 5 |
| Riccitelli G | 2012 | 13100158 | Multiple Sclerosis | 78 | 88 | 42 |
| Ridler K | 2001 | 8090189 | Tuberous Sclerosis | 10 | 8 | 2 |
| Riederer F | 2012 | 16030053 | Chronic Pain | 29 | 29 | 8 |
| Riederer F | 2008 | 9050060 | Epilepsy (Left Mesial Temporal Lobe) | 9 | 12 | 10 |
| Riederer F | 2008 | 9050060 | Epilepsy (Right Mesial Temporal Lobe) | 13 |  | 3 |
| Riva D | 2011 | 12060008 | Autism Spectrum Disorder | 21 | 21 | 13 |
| Rocca M A | 2014 | 16030033 | Multiple Sclerosis (Non-Fatigued) | 32 | 35 | 4 |
| Rocca M A | 2014 | 16030033 | Multiple Sclerosis (Fatigued) | 31 |  | 12 |
| Rocca M A | 2006 | 8060223 | Chronic Pain (Migraine With Aura) | 7 | 15 | 9 |
| Rocca M A | 2006 | 8060223 | Chronic Pain (Migraine Without Aura) | 9 |  | 14 |
| Rocha-Rego V | 2012 | 14110031 | Post Traumatic Stress Disorder | 16 | 16 | 2 |
| Rodriguez-Raecke R | 2009 | 13110208 | Chronic Pain | 32 | 32 | 16 |
| Rodriguez-Raecke R | 2013 | 16030054 | Chronic Pain (Osteoarthritis) (Left Primary) | 7 | 20 | 1 |
| Rodriguez-Raecke R | 2013 | 16030054 | Osteoarthritis (Osteoarthritis) (Right Primary) | 20 |  | 10 |
| Roman-Urrestarazu A | 2016 | 19120019 | Attention Deficit Hyperactivity Disorder | 49 | 34 | 1 |
| Rosen H J | 2002 | 8060224 | Frontotemporal Lobar Degeneration | 20 | 20 | 8 |
| Rossi R | 2012 | 17050008 | Borderline Personality Disorder | 26 | 26 | 39 |
| Rowe J B | 2010 | 21020011 | Parkinson's Disease | 82 | 82 | 1 |
| Ruscheweyh R | 2011 | 11080302 | Chronic Pain | 45 | 31 | 26 |
| Salmond C H | 2000 | 8050118 | Amnesia | 5 | 8 | 8 |
| Salmond C H | 2005 | 8050117 | Autism Spectrum Disorder | 14 | 13 | 2 |
| Salmond C H | 2007 | 12060009 | Autism Spectrum Disorder (Low Functioning) | 9 | 9 | 6 |
| Sanchis-Segura C | 2016 | 17070012 | Multiple Sclerosis (Female) | 34 | 18 | 4 |
| Sanchis-Segura C | 2016 | 17070012 | Multiple Sclerosis (Male) | 22 | 35 | 6 |
| Santana M | 2010 | 10060066 | Epilepsy (Left Mesial Temporal Lobe) | 59 | 30 | 16 |
| Santana M | 2010 | 10060066 | Epilepsy (Right Mesial Temporal Lobe) | 41 |  | 10 |
| Santos M C A | 2016 | 23100043 | Parkinson's Disease (Right Onset) | 24 | 80 | 7 |
| Santos M C A | 2016 | 23100043 | Parkinson's Disease (Left Onset) | 36 |  | 4 |
| Sasayama D | 2010 | 13100160 | Attention Deficit Hyperactivity Disorder | 10 | 17 | 9 |
| Saykin A J | 2006 | 8050119 | Mild Cognitive Impairment (Cognitive Complaints) | 40 | 40 | 12 |
| Saykin A J | 2006 | 8050119 | Mild Cognitive Impairment (Non-Cognitive Complaints) | 40 |  | 15 |
| Schiffer B | 2007 | 8050120 | Pedophilic Disorder | 18 | 24 | 2 |
| Schmidt-Wilcke T | 2006 | 8050122 | Chronic Pain (Migraine) | 18 | 18 | 5 |
| Schmidt-Wilcke T | 2007 | 9050062 | Chronic Pain (Fibromyalgia) | 20 | 22 | 2 |
| Schmidt-Wilcke T | 2005 | 11030006 | Chronic Pain (Chronic Tension Headache) | 20 | 40 | 16 |
| Schmidt-Wilcke T | 2008 | 11030005 | Chronic Pain (Migraine) | 35 | 31 | 4 |
| Schmidt-Wilcke T | 2009 | 11040205 | Mild Cognitive Impairment | 18 | 18 | 4 |
| Schmidt-Wilcke T | 2010 | 10060067 | Chronic Pain (Idiopathic Facial Pain) | 11 | 11 | 9 |
| Schumacher J | 2019 | 23100044 | Alzheimer's Disease | 28 | 22 | 21 |
| Schumacher J | 2019 | 23100044 | Lewy Body Dementia | 39 |  | 21 |
| Schwartz D L | 2010 | 10030032 | Substance Use Disorder | 61 | 44 | 4 |
| Seeley W W | 2008 | 13100065 | Frontotemporal Lobar Degeneration (CDR score of 0.5) | 15 | 45 | 29 |
| Seeley W W | 2008 | 13100065 | Frontotemporal Lobar Degeneration (CDR score of 1) | 15 |  | 33 |
| Seeley W W | 2008 | 13100065 | Frontotemporal Lobar Degeneration (CDR score of 2-3) | 15 |  | 43 |
| Seidman L J | 2019 | 19120020 | Attention Deficit Hyperactivity Disorder | 74 | 54 | 3 |
| Seminowicz D A | 2010 | 13100161 | Chronic Pain | 56 | 49 | 18 |
| Senda J | 2011 | 14030006 | Amyotrophic Lateral Sclerosis | 17 | 17 | 6 |
| Sepulcre J | 2006 | 8050123 | Multiple Sclerosis | 31 | 15 | 9 |
| Sethi A | 2017 | 19120021 | Attention Deficit Hyperactivity Disorder | 30 | 30 | 2 |
| Sheelakumari R | 2020 | 22050048 | Frontotemporal Dementia | 25 | 20 | 34 |
| Shen D | 2018 | 21060019 | Amyotrophic Lateral Sclerosis | 11 | 20 | 6 |
| Shigomoto Y | 2013 | 21100045 | Multiple System Atrophy | 20 | 30 | 9 |
| Shiino A | 2006 | 9050064 | Alzheimer's Desease | 40 | 88 | 16 |
| Shiino A | 2006 | 9050064 | Mild Cognitive Impairment | 20 |  | 10 |
| Shin S | 2012 | 13110215 | Parkinson's Disease (With Visual Hallucinations) | 46 | 25 | 20 |
| Shin S | 2012 | 13110215 | Parkinson's Disease (Without Visual Hallucinations) | 64 |  | 1 |
| Shott M E | 2015 | 18100011 | Binge Eating Disorder | 18 | 24 | 8 |
| Silani G | 2005 | 8060227 | Specific Learning Disorder | 32 | 32 | 1 |
| Simon T J | 2005 | 8090193 | 22q11.2 Deletion Syndrome | 18 | 18 | 8 |
| Sobanski T | 2010 | 11040145 | Panic Disorder | 17 | 17 | 2 |
| Sowell E R | 2001 | 8050125 | Fetal Alcohol Syndrome | 21 | 21 | 17 |
| Spano B | 2010 | 11040207 | Multiple Sclerosis | 10 | 20 | 12 |
| Specht K | 2003 | 8050127 | Multiple-System Atrophy | 14 | 13 | 1 |
| Spencer M D | 2006 | 8050129 | Intellectual Disability | 63 | 72 | 2 |
| Steinbach R | 2021 | 21060020 | Amyotrophic Lateral Sclerosis (Bulb-Onset) | 26 | 69 | 3 |
| Steinbach R | 2021 | 21060020 | Amyotrophic Lateral Sclerosis (Limb-Onset) | 52 |  | 1 |
| Steinbrink C | 2008 | 9050065 | Specific Learning Disorder | 7 | 7 | 2 |
| Stevens M C | 2019 | 19120016 | Attention Deficit Hyperactivity Disorder | 24 | 24 | 1 |
| Strawn J R | 2013 | 21110107 | Generalized Anxiety Disorder | 15 | 28 | 2 |
| Suchan B | 2010 | 11040077 | Anorexia Nervosa | 15 | 15 | 2 |
| Sui S G | 2010 | 13100070 | Post Traumatic Stress Disorder | 11 | 12 | 11 |
| Summerfield C | 2005 | 8050131 | Parkinson's Disease (Without Dementia) | 13 | 13 | 3 |
| Summerfield C | 2005 | 8050131 | Parkinson's Disease (With Dementia) | 16 |  | 10 |
| Sunol M | 2022 | 22030022 | Chronic Pain (Fibromyalgia) | 34 | 38 | 1 |
| Swartz B E | 2016 | 21070049 | Epilepsy (Juvenile Myoclonic) | 14 | 14 | 4 |
| Szesko P R | 2008 | 11040103 | Obsessive Compulsive Disorder | 37 | 26 | 3 |
| Tae W S | 2006 | 8050133 | Epilepsy (Juvenile Myoclonic) | 19 | 19 | 6 |
| Tae W S | 2010 | 10060071 | Epilepsy (Mesial Temporal Lobe Left) | 16 | 23 | 28 |
| Tae W S | 2010 | 10060071 | Epilepsy (Mesial Temporal Lobe Right) | 15 |  | 31 |
| Takahashi R | 2010 | 13100171 | Alzheimer's Disease | 51 | 40 | 6 |
| Takahashi R | 2010 | 13100171 | Lewy Body Dementia | 43 |  | 6 |
| Takahashi R | 2011 | 13100172 | Progressive Supranuclear Palsy | 16 | 20 | 7 |
| Tanabe J | 2009 | 9050067 | Substance Use Disorder | 19 | 20 | 1 |
| Tavanti M | 2012 | 13100174 | Post Traumatic Stress Disorder | 25 | 25 | 26 |
| Tavazzi E | 2012 | 16020009 | Amyotrophic Lateral Sclerosis | 20 | 31 | 7 |
| Tavazzi E | 2012 | 16020009 | Multiple Sclerosis | 18 |  | 17 |
| Terada T | 2018 | 23100047 | Parkinson's Disease | 40 | 10 | 9 |
| Tessitore A | 2012 | 13100200 | Parkinson's Disease | 12 | 12 | 2 |
| Thivard L | 2007 | 13100175 | Amyotrophic Lateral Sclerosis | 15 | 25 | 19 |
| Thomaes K | 2010 | 13100176 | Post Traumatic Stress Disorder | 31 | 28 | 5 |
| Tiihonen J | 2008 | 9050069 | Psychopathy | 12 | 25 | 2 |
| Tiihonen J | 2008 | 9050069 | Antisocial Personality Disorder | 26 |  | 31 |
| Tir M | 2009 | 11040150 | Multiple System Atrophy | 14 | 14 | 1 |
| Tir M | 2009 | 11040150 | Parkinson's Desease | 19 |  | 2 |
| Toal F | 2010 | 12060010 | Autism Spectrum Disorder | 65 | 33 | 3 |
| Togao O | 2010 | 13100178 | Obsessive Compulsive Disorder | 23 | 26 | 6 |
| Tomoda A | 2009 | 11050264 | Dissociative Identity Disorder | 23 | 14 | 1 |
| Torelli F | 2011 | 13100179 | Obstructive Sleep Apnea | 16 | 14 | 1 |
| Touvinen T | 2017 | 22060091 | Alzheimer's Disease | 23 | 25 | 1 |
| Touvinen T | 2017 | 22060091 | Frontotemporal Dementia | 21 |  | 1 |
| Tse N Y | 2020 | 21050003 | Corticobasal Degeneration | 16 | 33 | 14 |
| Tse N Y | 2020 | 21050003 | Progressive Supranuclear Palsy | 16 |  | 10 |
| Tu C H | 2010 | 11030008 | Chronic Pain (Primary Dysmenorrhea) | 32 | 32 | 9 |
| Tu S | 2015 | 22060090 | Alzheimer's Disease | 20 | 23 | 2 |
| Tu S | 2015 | 22060090 | Frontotemporal Dementia | 24 |  | 1 |
| Tu S | 2015 | 22060090 | Semantic Dementia | 14 |  | 1 |
| Tzarouchi L C | 2010 | 11040064 | Multiple System Atrophy | 11 | 11 | 31 |
| Uchida R R | 2008 | 9050085 | Panic Disorder | 19 | 20 | 1 |
| Ung H | 2012 | 16030055 | Chronic Pain | 47 | 47 | 3 |
| Valente A A Jr | 2005 | 8060229 | Obsessive Compulsive Disorder | 19 | 15 | 2 |
| Valet M | 2009 | 11020003 | Chronic Pain | 14 | 25 | 13 |
| Valfre W | 2008 | 9050072 | Chronic Pain (Migraine) | 27 | 27 | 11 |
| van de Pavert S H | 2015 | 16030039 | Multiple Sclerosis (Primary Progressive) | 25 | 30 | 7 |
| van de Pavert S H | 2015 | 16030039 | Multiple Sclerosis (Relapsing Remitting) | 30 |  | 3 |
| van de Pavert S H | 2015 | 16030039 | Multiple Sclerosis (Secondary Progressive) | 25 |  | 10 |
| Van den Heuvel O A | 2009 | 11040186 | Obsessive Compulsive Disorder | 55 | 50 | 5 |
| van Tol M J | 2010 | 13100073 | Anxiety Disorder | 68 | 65 | 1 |
| van Wingen G | 2013 | 19120027 | Attention Deficit Hyperactivity Disorder (Without Cocaine Dependence) | 14 | 15 | 2 |
| van Wingen G | 2013 | 19120027 | Attention Deficit Hyperactivity Disorder (With Cocaine Dependence) | 10 |  | 2 |
| Vannorsdall T D | 2010 | 11040211 | Traumatic Brain Injury | 14 | 28 | 6 |
| Vargha-Khadem F | 2003 | 8090199 | Hypoxic-ischemic Damage | 11 | 16 | 9 |
| Vartiainen N | 2009 | 11040151 | Herpes Simplex Virus | 8 | 28 | 7 |
| Villemointeix T | 2015 | 19120022 | Attention Deficit Hyperactivity Disorder (Met158-Carriers) | 28 | 15 | 1 |
| Villemointeix T | 2015 | 19120022 | Attention Deficit Hyperactivity Disorder (Val158 homozygotes) | 10 | 25 | 1 |
| Wang G | 2015 | 23100048 | Progressive Supranuclear Palsy | 24 | 23 | 50 |
| Wang H | 2022 | 22020014 | Autism Spectrum Disorder (Pediatric) | 24 | 19 | 2 |
| Wang H | 2022 | 22020014 | Autism Spectrum Disorder (Adult) | 10 | 13 | 1 |
| Wang J | 2007 | 13100076 | Attention Deficit Hyperactivity Disorder | 12 | 12 | 4 |
| Wang L | 2019 | 19120013 | Attention Deficit Hyperactivity Disorder | 30 | 25 | 3 |
| Waragai M | 2009 | 10030024 | Alzheimer's Disease | 15 | 12 | 2 |
| Watkins K E | 2002 | 8060231 | Language Disorder | 10 | 17 | 10 |
| Weber Y G | 2010 | 10060073 | Myotonic Dystrophy (Type 1) | 14 | 20 | 17 |
| Weber Y G | 2010 | 10060073 | Myotonic Dystrophy (Type 2) | 9 | 18 | 15 |
| Wei G | 2020 | 22050066 | Alzheimer's Disease | 20 | 28 | 5 |
| Wei G | 2020 | 22050066 | Frontotemporal Dementia (Early) | 22 |  | 5 |
| Wei G | 2020 | 22050066 | Frontotemporal Dementia (Late) | 22 |  | 1 |
| Wei W | 2016 | 16070065 | Epilepsy (Medial Temporal With Hippocampal Sclerosis) | 30 | 30 | 11 |
| Wei W | 2016 | 16070065 | Epilepsy (Medial Temporal With Other Lesions) | 30 |  | 4 |
| White N S | 2003 | 8050143 | Down Syndrome | 19 | 11 | 28 |
| Whitwell J L | 2005 | 8090202 | FrontoTemporal Dementia (Ubiquitin-Positive) | 9 | 20 | 3 |
| Whitwell J L | 2005 | 8090202 | FrontoTemporal Dementia (Pick Disease) | 7 |  | 6 |
| Whitwell J L | 2005 | 8090202 | FrontoTemporal Dementia (Ubiquitin-Negative) | 5 |  | 2 |
| Whitwell J L | 2007 | 11080304 | Frontotemporal Lobar Degeneration (With Hyperphagia) | 7 | 9 | 2 |
| Whitwell J L | 2007 | 11080304 | Frontotemporal Lobar Degeneration (With a Pathological Sweet Tooth) | 9 |  | 13 |
| Whitwell J L | 2007 | 8050145 | Alzheimer's Desease | 38 | 38 | 1 |
| Whitwell J L | 2007 | 8050145 | Posterior Cortical Atrophy | 38 |  | 1 |
| Whitwell J L | 2013 | 14050010 | Primary Progressive Apraxia | 16 | 20 | 4 |
| Whitwell J L | 2013 | 14050010 | Progressive Supranuclear Palsy | 16 |  | 8 |
| Whitwell J L | 2009 | 11040214 | Frontotemporal Dementia (IVS10+16 MAPT Mutation) | 4 | 19 | 1 |
| Whitwell J L | 2009 | 11040214 | Various Neurodegenerative Diseases (IVS10+3 MAPT Mutation) | 3 |  | 1 |
| Whitwell J L | 2009 | 11040214 | Frontotemporal Dementia (N279K MAPT Mutation) | 3 |  | 1 |
| Whitwell J L | 2009 | 11040214 | Frontotemporal Dementia (P301L MAPT Mutation) | 4 |  | 1 |
| Whitwell J L | 2009 | 11040214 | Frontotemporal Dementia (S305N MAPT Mutation) | 2 |  | 1 |
| Whitwell J L | 2009 | 11040214 | Frontotemporal Dementia (V337M MAPT Mutation) | 3 |  | 1 |
| Whitwell J L | 2004 | 13100185 | Frontotemporal Lobar Degeneration (Tau-Negative) | 8 | 20 | 3 |
| Whitwell J L | 2004 | 13100185 | Frontotemporal Lobar Degeneration (Tau-Positive) | 9 |  | 10 |
| Wiest R | 2005 | 8090203 | Epilepsy (Mesial Temporal Lobe) | 7 | 30 | 7 |
| Wilson N | 2020 | 22050067 | Alzheimer's Disease | 18 | 25 | 5 |
| Wilson N | 2020 | 22050067 | Frontotemporal Dementia | 19 |  | 1 |
| Wilson S M | 2009 | 13100186 | Semantic Dementia | 5 | 48 | 15 |
| Woermann F G | 2000 | 9080103 | Epilepsy (Temporal Lobe With Intermittent Explosive Disorder) | 25 | 35 | 2 |
| Woermann F G | 2000 | 9080103 | Epilepsy (Temporal Lobe Without Intermittent Explosive Disorder) | 25 |  | 1 |
| Wolf R C | 2009 | 9050080 | Huntington's Disease | 12 | 16 | 14 |
| Wood P B | 2009 | 11040235 | Chronic Pain (Fibromyalgia) | 30 | 20 | 4 |
| Wu Y | 2020 | 23100049 | Parkinson's Disease | 30 | 15 | 3 |
| Xia J | 2013 | 23100050 | Parkinson's Disease | 32 | 25 | 18 |
| Xiao J X | 2007 | 11080287 | Amblyopia | 13 | 14 | 4 |
| Xie S | 2006 | 8090204 | Alzheimer's Desease | 13 | 16 | 9 |
| Xuan M | 2019 | 23100051 | Parkinson's Disease (Early Onset) | 28 | 23 | 5 |
| Xuan M | 2019 | 23100051 | Parkinson's Disease (Middle-Late Onset) | 37 | 23 | 5 |
| Yang F C | 2013 | 16030056 | Chronic Pain (Migraine) (Left-Sided Attack) | 26 | 49 | 13 |
| Yang F C | 2013 | 16030056 | Chronic Pain (Migraine) (Right-Sided Attack) | 23 |  | 33 |
| Yang Q | 2018 | 19120025 | Autism Spectrum Disorder | 16 | 16 | 3 |
| Yaouhi K | 2009 | 11040106 | Obstructive Sleep Apnea | 16 | 14 | 7 |
| Yasuda C L | 2010 | 11040153 | Epilepsy (With Negative Family History of Epilepsy) | 29 | 69 | 13 |
| Yasuda C L | 2010 | 11040153 | Epilepsy (With Positive Family History of Epilepsy) | 40 |  | 13 |
| Yasuda C L | 2010 | 11040039 | Epilepsy (Seizure Free) | 34 | 69 | 23 |
| Yasuda C L | 2010 | 11040039 | Epilepsy (With Worthwhile Improvement) | 23 |  | 12 |
| Yasuda C L | 2010 | 11040039 | Epilepsy (Whitout Improvement) | 10 |  | 30 |
| Yin C | 2014 | 21010001 | Mild Cognitive Impairment | 11 | 22 | 5 |
| Yoneyama E | 2003 | 8090206 | Schizotypal Personality Disorder | 14 | 28 | 3 |
| Yoo H K | 2005 | 8050150 | Panic Disorder | 18 | 18 | 7 |
| Yoo S Y | 2008 | 11040184 | Obsessive Compulsive Disorder (Female) | 24 | 24 | 4 |
| Yoo S Y | 2009 | 11040185 | Obsessive Compulsive Disorder (Male) | 47 | 47 | 5 |
| Yoon E J | 2013 | 16030057 | Chronic Pain (Spinal Cord Injury) | 10 | 10 | 4 |
| Younger J W | 2010 | 10060053 | Chronic Pain (Chronic Myofascial Temporomandibular Pain) | 14 | 15 | 1 |
| Zahn R | 2005 | 13100189 | Alzheimer's Disease | 10 | 10 | 4 |
| Zahn R | 2005 | 13100189 | Progressive Non Fluent Aphasia | 5 |  | 4 |
| Zamboni G | 2008 | 11040079 | Frontotemporal Dementia | 62 | 14 | 11 |
| Zhang J | 2011 | 13100080 | Post Traumatic Stress Disorder | 10 | 10 | 3 |
| Zhang J | 2018 | 21080075 | Conduct Disorder | 60 | 60 | 3 |
| Zhang J | 2015 | 23100052 | Parkinson's Disease (With Mild Cognitive Impairment) | 21 | 20 | 5 |
| Zhang J | 2015 | 23100052 | Parkinson's Disease (Without Mild Cognitive Impairment) | 14 |  | 7 |
| Zhang X | 2016 | 17070013 | Multiple Sclerosis | 39 | 29 | 4 |
| Zhang X | 2018 | 21120121 | Post Traumatic Stress Disorder | 35 | 36 | 1 |
| Zhang Y | 2014 | 21100099 | With Mild Cognitive Impairment | 28 | 29 | 19 |
| Zhao Y | 2019 | 19120026 | Attention Deficit Hyperactivity Disorder | 36 | 36 | 6 |
| Zhao Z | 2014 | 21010002 | Mild Cognitive Impairment | 20 | 18 | 7 |
| Zhong M | 2022 | 23100054 | Parkinson's Disease (With Hallucinations) | 23 | 31 | 4 |
| Zhong M | 2022 | 23100054 | Parkinson's Disease (Without Hallucinations) | 35 |  | 4 |
| **TOTAL** | | | | **17450** | **15346** | **6564** |

**eTable 6.** Distribution of the VBM data included (main analysis)

| **BACON**  **data sets** | **Experiments** | | **Patients** | | **Controls** | **Foci of variation** | |
| --- | --- | --- | --- | --- | --- | --- | --- |
|  | **N** | **%** | **N** | **%** | **N** | **N** | **%** |
| BD | 73 | 16.1 | 2959 | 18 | 17139* | 420 | 13 |
| MDD | 82 | 18.1 | 4116 | 25 |  | 461 | 14 |
| SZ | 123 | 27.1 | 5015 | 31 |  | 1295 | 39 |
| BrainMap | 175 | 38.7 | 4246 | 26 |  | 1137 | 34 |
| **Total** | 453 | 100 | 16336 | 100 | 17139 | 3313 | 100 |

*Multiple experimental groups share certain control groups. Details can be seen in the eTables 1-5. N = number; % = percentage.

**eTable 7.** Distribution of the VBM data included (additional analysis)

| **BACON**  **data sets** | **Experiments** | | **Patients** | | **Controls** | **Foci of variation** | |
| --- | --- | --- | --- | --- | --- | --- | --- |
|  | **N** | **%** | **N** | **%** | **N** | **N** | **%** |
| BD | 73 | 7.1 | 2959 | 10 | 28177* | 420 | 4.8 |
| MDD | 82 | 8 | 4116 | 13.9 |  | 461 | 5.3 |
| SZ | 123 | 12 | 5015 | 16.9 |  | 1295 | 14.8 |
| BrainMap | 743 | 72.9 | 17450 | 59.2 |  | 6564 | 75.1 |
| **Total** | 1021 | 100 | 29540 | 100 | 28177 | 8740 | 100 |

*Multiple experimental groups share certain control groups. Details can be seen in the eTables 1-5. N = number; % = percentage.

**eTable 8.** Meta-analytic connectivity modeling (MACM) of the right middle temporal gyrus in bipolar disorder

| **Cluster**  **ID** | **Talairach** | | | **ALE**  **value** | **P**  **value** | **Local maximum**  **(brain area)** |
| --- | --- | --- | --- | --- | --- | --- |
|  | **x** | **y** | **z** |  |  |  |
| 1 | 52 | -62 | 22 | 0.080103904 | 1,05E-24 | Right Superior Temporal Gyrus (BA 39) |
|  | 46 | -68 | 34 | 0.016626408 | 5,77E+01 | Right Middle Temporal Gyrus (BA 39) |
| 2 | -46 | -68 | 22 | 0.029632468 | 3,14E-02 | Left Middle Temporal Gyrus (BA 39) |
| 3 | 2 | -56 | 24 | 0.029390708 | 3,82E-02 | Left Posterior Cingulate Cortex (BA 31) |
| 4 | 62 | -10 | -18 | 0.027509483 | 1,76E-01 | Right Inferior Temporal Gyrus (BA 21) |
| 5 | -54 | -10 | -18 | 0.02279647 | 6,77E-02 | Left Inferior Temporal Gyrus (BA 21) |

ALE = activation likelihood estimation; BA = Brodmann area. Results are family-wise error-corrected (FWE-c) for multiple comparisons, with a cluster-level inference of p < .05 and a cluster-forming threshold of p < .001 on the voxel level (1,000 permutation runs).

**eTable 9.** Behavioral results of the right middle temporal gyrus MACM

| **Category**  **(sub-category)** | **Domain** | **Z**  **score** |
| --- | --- | --- |
| Social cognition | Cognition | 6.528 |
| Memory (Explicit) | Cognition | 4.439 |
| Language (Semantics) | Cognition | 4.214 |
| Reasoning | Cognition | 3.71 |
| Language (Syntax) | Cognition | 3.586 |

The green color represents the cognition behavioral domain. A threshold of p < .05 with Bonferroni correction for multiple comparisons was applied, corresponding to a subdomain z-score $\geq$ 3.

**eTable 10.** Meta-analytic connectivity modeling (MACM) of the right anterior cingulate cortex in schizophrenia

| **Cluster**  **ID** | **Talairach** | | | **ALE**  **value** | **P**  **value** | **Local maximum**  **(brain area)** |
| --- | --- | --- | --- | --- | --- | --- |
|  | **x** | **y** | **z** |  |  |  |
| 1 | 10 | 22 | 30 | 0.19263557 | 0.0 | Right Anterior Cingulate cortex (BA 32) |
|  | 2 | 10 | 46 | 0.045234267 | 8,89E-04 | Right Medial Frontal Gyrus (BA 32) |
|  | -10 | 12 | 40 | 0.04467176 | 1,28E-02 | Left Anterior Cingulate cortex (BA 32) |
|  | 20 | 10 | 40 | 0.044180356 | 1,76E-02 | Right Medial Frontal Gyrus (BA 32) |
|  | 28 | 0 | 44 | 0.027906436 | 2,84E+02 | Right Middle Frontal Gyrus (BA 6) |
|  | -6 | 0 | 60 | 0.026236162 | 6,92E+01 | Left Middle Frontal Gyrus (BA 6) |
| 2 | -32 | 16 | 6 | 0.061628003 | 9,67E-08 | Left Insula (BA 13) |
|  | -50 | 12 | 16 | 0.041779596 | 8,17E-02 | Left Inferior Frontal Gyrus (BA 44) |
|  | -42 | 4 | 32 | 0.040963173 | 1,37E-01 | Left Precentral Gyrus (BA 9) |
|  | -40 | 8 | 28 | 0.039663125 | 3,09E-01 | Left Inferior Frontal Gyrus (BA 9) |
| 3 | 32 | 20 | 2 | 0.06779873 | 9,29E-11 | Right Insula (BA 13) |
| 4 | -28 | -52 | 40 | 0.043961607 | 2,03E-02 | Left Superior Parietal Lobule (BA 7) |
|  | -44 | -46 | 42 | 0.030400276 | 7,26E+01 | Left Inferior Parietal Lobule (BA 40) |
|  | -26 | -72 | 32 | 0.029695272 | 1,07E+02 | Left Precuneus (BA 19) |
| 5 | 22 | 6 | 2 | 0.03569689 | 3,42E+00 | Right Lentiform Nucleus (Putamen) |
|  | 8 | -6 | 12 | 0.03114084 | 4,80E+00 | Right Thalamus (Anterior Nucleus) |
| 6 | 44 | 10 | 26 | 0.03754674 | 1,13E+01 | Right Inferior Frontal Gyrus (BA 9) |
|  | 42 | 22 | 30 | 0.029058801 | 1,52E+02 | Right Middle Frontal Gyrus (BA 9) |
|  | 40 | 6 | 38 | 0.027143944 | 4,30E+00 | Right Precentral Gyrus (BA 9) |
| 7 | 26 | -60 | 38 | 0.039743364 | 2,94E-01 | Right Precuneus (BA 7) |
|  | 30 | -52 | 44 | 0.028613428 | 1,95E+02 | Right Superior Parietal Lobule (BA 7) |
|  | 36 | -58 | 50 | 0.026098123 | 7,44E+01 | Right Superior Parietal Lobule (BA 7) |
| 8 | -14 | -10 | 4 | 0.027068632 | 4,46E+01 | Left Thalamus (Anterior Nucleus) |
|  | -14 | -4 | 4 | 0.024338601 | 1,85E+03 | Left Lentiform Nucleus (Lateral Globus Pallidus) |
| 9 | 24 | -86 | -4 | 0.026902352 | 4,88E+02 | Right Middle Occipital Gyrus (BA 18) |
|  | 24 | -78 | -12 | 0.025086954 | 1,26E+02 | Right Fusiform Gyrus (BA 19) |
| 10 | -42 | -54 | -14 | 0.031899877 | 3,12E+01 | Left Fusiform Gyrus (BA 37) |

ALE = activation likelihood estimation; BA = Brodmann area. Results are family-wise error-corrected (FWE-c) for multiple comparisons, with a cluster-level inference of p < .05 and a cluster-forming threshold of p < .001 on the voxel level (1,000 permutation runs).

**eTable 11.** Behavioral results of the right anterior cingulate cortex MACM

| **Category** | **Domain** | **Z** |
| --- | --- | --- |
| **(sub-category)** |  | **score** |
| Attention | Cognition | 34.29 |
| Working Memory | Cognition | 26.26 |
| Reasoning | Cognition | 25.495 |
| Semantics (Language) | Cognition | 24.778 |
| Speech (Language) | Cognition | 23.72 |
| Reward/Gain | Emotion | 18.19 |
| Pain | Perception | 17.99 |
| Inhibition | Action | 17.441 |
| Memory (Explicit) | Cognition | 17.321 |
| Phonology (Language) | Cognition | 17.017 |
| Execution | Action | 14.419 |
| Vision | Perception | 13.934 |
| Music | Cognition | 13.277 |
| Audition | Perception | 13.073 |
| Speech (Execution) | Cognition | 12.368 |
| Shape (Vision) | Perception | 12.362 |
| Orthography (Language) | Cognition | 12.065 |
| Negative (Emotion) | Emotion | 11.081 |
| Motion (Vision) | Perception | 10.466 |
| Space | Cognition | 10.142 |
| Social Cognition | Cognition | 9.804 |
| Syntax (Language) | Cognition | 9.594 |
| Color (Vision) | Perception | 9.377 |
| Somesthesis | Perception | 9.204 |
| Observation | Action | 8.62 |
| Sexuality | Interoception | 8.26 |
| Imagination | Action | 7.896 |
| Positive (Emotion) | Emotion | 7.893 |
| Thermoregulation | Interoception | 7.564 |
| Preparation | Action | 7.429 |
| Gustation | Perception | 6.419 |
| Disgust | Emotion | 5.921 |
| Fear | Emotion | 5.879 |
| Valence (Emotion) | Emotion | 5.837 |
| Language | Cognition | 5.686 |
| Anxiety | Emotion | 5.699 |
| Time | Cognition | 4.894 |
| Sadness (Emotion) | Emotion | 4.547 |
| Gastrointestinal | Interoception | 4.048 |
| Soma | Cognition | 3.749 |
| Punishment/Loss | Emotion | 3.74 |
| Intensity (Emotion) | Emotion | 3.598 |
| Sleep | Interoception | 3.574 |
| Anger | Emotion | 3.401 |
| Motor Learning | Action | 3.278 |
| Memory | Cognition | 3.248 |
| Embarrassment | Emotion | 3.247 |
| Happiness | Emotion | 3,247 |
| Olfaction | Perception | 3.233 |

Colors from red to orange represent different behavioral domains. A threshold of p < .05 with Bonferroni correction for multiple comparisons was applied, corresponding to a subdomain z-score $\geq$ 3.

**eReferences.**

Acar, F., Seurinck, R., Eickhoff, S. B., & Moerkerke, B. (2018). Assessing robustness against potential publication bias in Activation Likelihood Estimation (ALE) meta-analyses for fMRI. *PLOS ONE*, *13*(11), e0208177. doi: 10.1371/journal.pone.0208177

Cauda, F., Manuello, J., Crocetta, A., Duca, S., Costa, T., & Liloia, D. (2024). Meta-analytic connectivity perturbation analysis (MACPA): A new method for enhanced precision in fMRI connectivity analysis. *Brain Structure and Function*, *230*(1), 17. doi: 10.1007/s00429-024-02867-4

Chen, G., Wang, J., Gong, J., Qi, Z., Fu, S., Tang, G., … Wang, Y. (2022). Functional and structural brain differences in bipolar disorder: A multimodal meta-analysis of neuroimaging studies. *Psychological Medicine*, *52*(14), 2861–2873. doi: 10.1017/S0033291722002392

Costa, T., Manuello, J., Ferraro, M., Liloia, D., Nani, A., Fox, P. T., … Cauda, F. (2021). BACON: A tool for reverse inference in brain activation and alteration. *Human Brain Mapping*, *42*(11), 3343–3351. doi: 10.1002/hbm.25452

Eickhoff, S. B., Bzdok, D., Laird, A. R., Kurth, F., & Fox, P. T. (2012). Activation likelihood estimation meta-analysis revisited. *NeuroImage*, *59*(3), 2349–2361. doi: 10.1016/j.neuroimage.2011.09.017

Eickhoff, S. B., Nichols, T. E., Laird, A. R., Hoffstaedter, F., Amunts, K., Fox, P. T., … Eickhoff, C. R. (2016). Behavior, Sensitivity, and power of activation likelihood estimation characterized by massive empirical simulation. *NeuroImage*, *137*, 70–85. doi: 10.1016/j.neuroimage.2016.04.072

Fox, P. T., & Lancaster, J. L. (2002). Opinion: Mapping context and content: the BrainMap model. *Nature Reviews. Neuroscience*, *3*(4), 319–321. doi: 10.1038/nrn789

Gray, J. P., Müller, V. I., Eickhoff, S. B., & Fox, P. T. (2020). Multimodal Abnormalities of Brain Structure and Function in Major Depressive Disorder: A Meta-Analysis of Neuroimaging Studies. *The American Journal of Psychiatry*, *177*(5), 422. doi: 10.1176/appi.ajp.2019.19050560

Kass, R. E., & Raftery, A. E. (1995). Bayes Factors. *Journal of the American Statistical Association*, *90*(430), 773–795. doi: 10.1080/01621459.1995.10476572

Laird, A. R., Eickhoff, S. B., Kurth, F., Fox, P. M., Uecker, A. M., Turner, J. A., … Fox, P. T. (2009). ALE Meta-Analysis Workflows Via the Brainmap Database: Progress Towards A Probabilistic Functional Brain Atlas. *Frontiers in Neuroinformatics*, *3*, 23. doi: 10.3389/neuro.11.023.2009

Laird, A. R., Eickhoff, S. B., Rottschy, C., Bzdok, D., Ray, K. L., & Fox, P. T. (2013). Networks of task co-activations. *NeuroImage*, *80*, 505–514. doi: 10.1016/j.neuroimage.2013.04.073

Lancaster, J. L., Laird, A. R., Eickhoff, S. B., Martinez, M. J., Fox, P. M., & Fox, P. T. (2012). Automated regional behavioral analysis for human brain images. *Frontiers in Neuroinformatics*, *6*. doi: 10.3389/fninf.2012.00023

Liloia, D., Brasso, C., Cauda, F., Mancuso, L., Nani, A., Manuello, J., … Rocca, P. (2021). Updating and characterizing neuroanatomical markers in high-risk subjects, recently diagnosed and chronic patients with schizophrenia: A revised coordinate-based meta-analysis. *Neuroscience & Biobehavioral Reviews*, *123*, 83–103. doi: 10.1016/j.neubiorev.2021.01.010

Liloia, D., Cauda, F., Uddin, L. Q., Manuello, J., Mancuso, L., Keller, R., … Costa, T. (2023). Revealing the Selectivity of Neuroanatomical Alteration in Autism Spectrum Disorder via Reverse Inference. *Biological Psychiatry: Cognitive Neuroscience and Neuroimaging*, *8*(11), 1075–1083. doi: 10.1016/j.bpsc.2022.01.007

Manuello, J., Costa, T., Cauda, F., & Liloia, D. (2022). Six actions to improve detection of critical features for neuroimaging coordinate-based meta-analysis preparation. *Neuroscience & Biobehavioral Reviews*, *137*, 104659. doi: 10.1016/j.neubiorev.2022.104659

Müller, V. I., Cieslik, E. C., Laird, A. R., Fox, P. T., Radua, J., Mataix-Cols, D., … Eickhoff, S. B. (2018). Ten simple rules for neuroimaging meta-analysis. *Neuroscience and Biobehavioral Reviews*, *84*, 151–161. doi: 10.1016/j.neubiorev.2017.11.012

Orwin, R. G. (1983). A Fail-Safe N for Effect Size in Meta-Analysis. *Journal of Educational Statistics*, *8*(2), 157–159. doi: 10.2307/1164923

Robinson, J. L., Laird, A. R., Glahn, D. C., Lovallo, W. R., & Fox, P. T. (2010). Metaanalytic connectivity modeling: Delineating the functional connectivity of the human amygdala. *Human Brain Mapping*, *31*(2), 173–184. doi: 10.1002/hbm.20854

Tahmasian, M., Sepehry, A. A., Samea, F., Khodadadifar, T., Soltaninejad, Z., Javaheripour, N., … Eickhoff, C. R. (2019). Practical recommendations to conduct a neuroimaging meta-analysis for neuropsychiatric disorders. *Human Brain Mapping*, *40*(17), 5142–5154. doi: 10.1002/hbm.24746

Turkeltaub, P. E., Eickhoff, S. B., Laird, A. R., Fox, M., Wiener, M., & Fox, P. (2012). Minimizing within-experiment and within-group effects in Activation Likelihood Estimation meta-analyses. *Human Brain Mapping*, *33*(1), 1–13. doi: 10.1002/hbm.21186

Vanasse, T. J., Fox, P. M., Barron, D. S., Robertson, M., Eickhoff, S. B., Lancaster, J. L., & Fox, P. T. (2018). BrainMap VBM: An environment for structural meta-analysis. *Human Brain Mapping*, *39*(8), 3308–3325. doi: 10.1002/hbm.24078
